# Pulmonate slug evolution is reflected in the de novo genome of *Arion vulgaris* Moquin-Tandon, 1855

**DOI:** 10.1038/s41598-022-18099-7

**Published:** 2022-08-20

**Authors:** Zeyuan Chen, Özgül Doğan, Nadège Guiglielmoni, Anne Guichard, Michael Schrödl

**Affiliations:** 1 SNSB-Bavarian State Collection of Zoology, Münchhausenstr. 21, 81247 Munich, Germany; 2 Department Biology II, Ludwig-Maximilians-Universität, Planegg-Martinsried, 82152 Munich, Germany; 3 Department of Molecular Biology and Genetics, Faculty of Science, Sivas Cumhuriyet University, Sivas, Turkey; 4 Evolutionary Biology and Ecology, Université Libre de Bruxelles, 1050 Brussels, Belgium; 5 INRAE, Agrocampus Ouest, Université de Rennes, IGEPP, 35650 Le Rheu, France; 6 Univ. Rennes, CNRS, Inria, IRISA-UMR 6074, 35000 Rennes, France; 7 GeoBio-Center LMU, 80333 Munich, Germany

**Keywords:** Evolution, Genetics, Molecular biology

## Abstract

Stylommatophoran pulmonate land slugs and snails successfully completed the water-to-land transition from an aquatic ancestor and flourished on land. Of the 30,000 estimated species, very few genomes have so far been published. Here, we assembled and characterized a chromosome-level genome of the “Spanish” slug, *Arion vulgaris* Moquin-Tandon, 1855, a notorious pest land slug in Europe. Using this reference genome, we conclude that a whole-genome duplication event occurred approximately 93–109 Mya at the base of Stylommatophora and might have promoted land invasion and adaptive radiation. Comparative genomic analyses reveal that genes related to the development of kidney, blood vessels, muscle, and nervous systems had expanded in the last common ancestor of land pulmonates, likely an evolutionary response to the terrestrial challenges of gravity and water loss. Analyses of *A. vulgaris* gene families and positively selected genes show the slug has evolved a stronger ability to counteract the greater threats of external damage, radiation, and water loss lacking a protective shell. Furthermore, a recent burst of long interspersed elements in the genome of *A. vulgaris* might affect gene regulation and contribute to rapid phenotype changes in *A. vulgaris*, which might be conducive to its rapid adaptation and invasiveness.

## Introduction

Land slugs and snails (Mollusca: Gastropoda), which are often abundant in gardens, forests, fields, and orchards, are, for the most part, classified as stylommatophoran pulmonates. They have radiated into about 30,000 species, have highly successfully colonized habitats from polar regions to the tropics, and some are well-known invasive species or pests across the world^1–5^. Stylommatophoran pulmonates are among the few representatives of mollusks that have colonized the terrestrial environment. The changes in the physical and chemical properties of the environment are immense for animals moving from aquatic to terrestrial environments, and these changes could affect all possible life processes, from respiration and excretion to methods of movement, the functioning of sense organs, and reproduction^6^. Overcoming drought, for example, is one of the biggest challenges in water-land transition^7^. Compared to land snails, the lack of a protective shell in land slugs seems to have further increased the difficulty in coping with external stimuli, predators, sun exposure, and drought. Land slugs have evolved certain innovations, such as defense by chemical compounds or behavior, to counteract these challenges^1,8^. However, the lack of shell also gives advantages such as reduced weight and lower energy costs, reduced dependence on calcium uptake, better mobility, and ability to occupy small spaces. Recently, comparative genomics methods have provided key perspectives for revealing the process of water-land transition and illuminated adaptive mechanisms^9–11^. With the rapid development of genome sequencing, several land snail genomes have been published (Supplementary data 1), however, the genomic resources for land slugs are still lacking.

In recent years, the notorious “Spanish” slug, *Arion vulgaris* Moquin-Tandon, 1855, has attracted widespread attention due to its invasiveness and negative impact on the economy, ecology, health, and social system^12^. As a major defoliator of plants, *A. vulgaris* causes serious damage in orchard cultivation, gardens, and agriculture resulting in financial losses^13–15^. *Arion vulgaris* also transmits plant pathogens, contaminates silage, and might cause health problems in animals^16,17^. It also outcompetes native slug species and reduces biodiversity^18^. Delivering Alien Invasive Species Inventoried for Europe (DAISIE) has listed *A. vulgaris* as one of the 100 worst alien species in Europe^19^, and it is the only land gastropod in the list. Although recent studies disputed the origin and invasiveness of *A. vulgaris* based on the genetic diversity patterns of mitochondrial and nuclear loci^20–22^, its outstanding adaptability and mass occurrences are undeniable.

Here, we assembled and annotated the first land slug genome—*A. vulgaris*. By comparing *A. vulgaris* with two stylommatophoran land snails, and stylommatophoran species with other aquatic or marine gastropods, the well-annotated genome provides a broader perspective to decipher the water-land transition process of stylommatophoran species. The *A. vulgaris* genome also provides insights into how shell-less *A. vulgaris* adapted to terrestrial environments and the underlying molecular mechanisms (e.g., whole-genome duplication (WGD), small scale gene duplication, transposable elements explosion)*.* Moreover, the high-quality genome provides an important reference for future research on *A. vulgaris* population genetics and mollusk evolutionary trajectories, e.g., the loss and evolution of mollusk shells^23^.

## Results

### Arion* vulgaris* genome assembly and annotation

**Figure 1:**
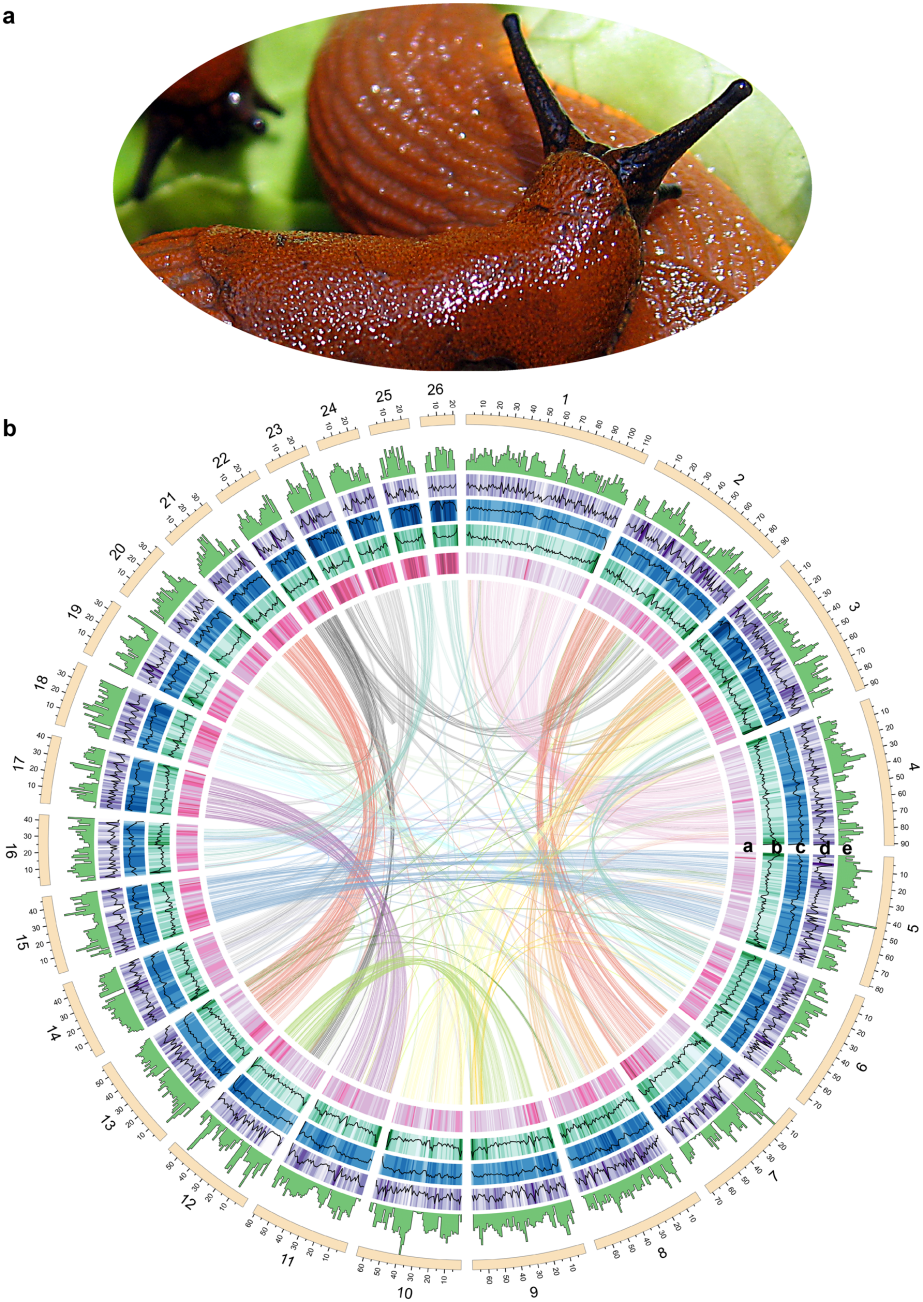
Genome features of *Arion vulgaris*. (**a**) Adult *A. vulgaris*. (**b**) General characteristics of the *A. vulgaris* genome. Tracks from inside to outside correspond to (**a**) GC content, (**b**) LTRs density, (**c**) TEs density, (**d**) genes density, and (**e**) heterozygosity in sliding windows of 1 Mb across each of the 26 pseudochromosomes. Inner lines connect syntenic genes due to ancestral whole-genome duplication events.

The genome size of *A. vulgaris* (Fig. 1a) is estimated to be around 1.45 Gb from *k*-mer analysis with short reads (Supplementary Fig. S1; Supplementary Table S1). We sequenced 75 Gb (52x) of long reads (mean length 19.39 kb, N50 length 25.80 kb) using Oxford Nanopore sequencing technology to produce a draft genome assembly. The draft assembly was polished using a combination of 57 Gb (40x) Illumina short reads and 138 Gb (95x) 10X Genomics linked reads. Next, the polished assembly was scaffolded using linked reads and then improved into a chromosome-level assembly with 135 Gb (93x) Hi-C data (Supplementary Table S1). Finally, we obtained an assembly with a total length of 1.54 Gb, a contig N50 of 8.6 Mb, and a scaffold N50 of 63.3 Mb, and with 93.8% of the sequences anchored onto 26 scaffolds (Supplementary Fig. S1–2; Supplementary Table S2). The number of chromosome-scale scaffolds is consistent with the species’ determined chromosome number based on karyotype studies^24^. We assessed the quality of the genome assembly in three aspects: (1) more than 95.99% of the Illumina short reads could be mapped to the assembly; (2) a total of 886 (90.59%) conserved genes in BUSCO’s metazoan (odb9) benchmark set^25^ were present and complete in the genome (Supplementary Table S2); (3) the *k*-mer distribution showed a relatively collapsed assembly including mostly single copies of the homozygous content and a partial representation of the heterozygous content, as is expected in a haploid assembly^26^ (Supplementary Fig. S3). These results all suggested a high-quality genomic resource of this initial genome assembly of *A. vulgaris*, which is comparable to other mollusk genomes, especially in a high level of heterozygosity and repeats content (Supplementary Fig. S4; Supplementary data 1). Gene annotation combining the evidence from transcripts, homologous proteins, and ab initio prediction revealed 32,518 predicted genes with an average length of 15,429 bp (Supplementary Table S4–5). The length distribution of transcripts, coding sequences, exons and introns, and the distribution of exon numbers per gene were comparable to that of other gastropods (Supplementary Fig. S5). Among the predicted protein-coding genes, 97.6% could be annotated through at least one of the following protein-related databases: the EggNOG^27^ database (51.64%), the Swiss-Prot^28^ protein database (97.57%), the Translated European Molecular Biology Laboratory (TrEMBL)^28^ database (96.65%), the protein families (Pfam)^29^ database (81.55%), and the Kyoto Encyclopedia of Genes and Genomes (KEGG)^30^ database (29.65%) (Supplementary Table S6).

### Phylogenetic relationships within gastropod lineages

**Figure 2:**
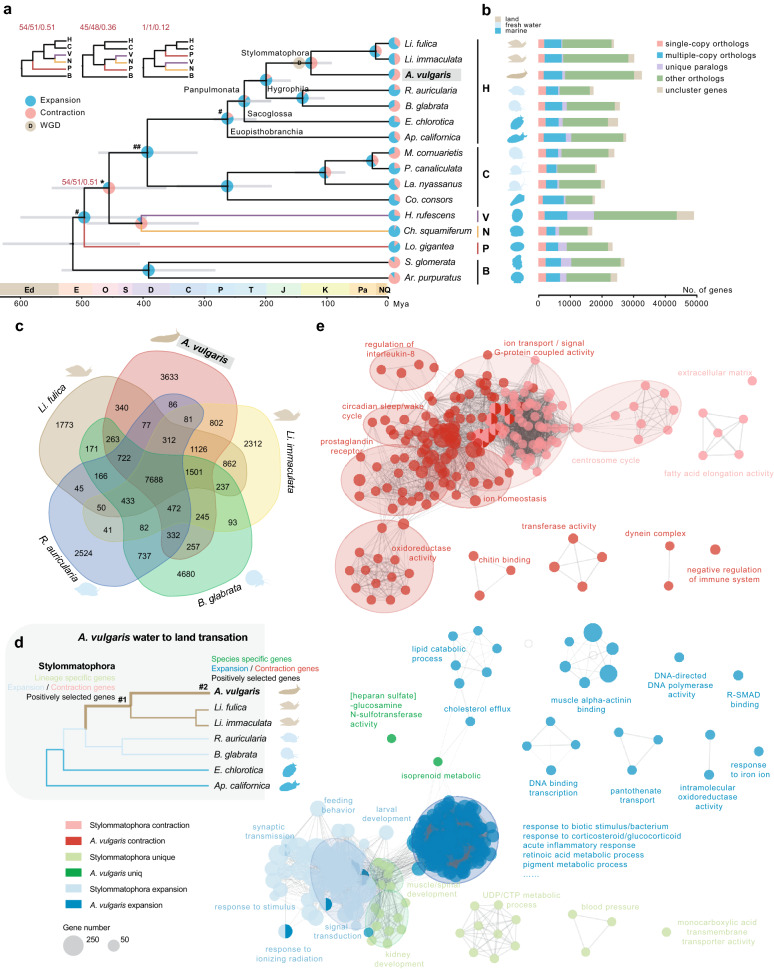
Phylogeny within gastropod lineages and gene family evolution. (**a**) Dated Maximum-likelihood phylogenetic tree among gastropod species using 223 single-copy orthologous genes. Grey lines indicate the 95% confidence intervals for the time of divergence between different clades. “#” indicates that nodes are constrained with fossil calibration and “##” indicate node is constrained with secondary calibration data. All nodes have bootstrap support values of 100 and posterior probabilities of 1 in all analyses, except the node with an asterisk. Three alternative topologies are shown on top left. Red numbers are bootstrap support percentages and posterior probabilities (from left to right, inferred by RaxML with GTR+L, IQ-TREE and ASTRAL respectively). H, Heterobranchia; C, Caenogastropoda; V, Vetigastropoda; N, Neomphalina; P, Patellogastropoda; B, Bivalve. The pie diagram on each branch of the tree represents the proportion of gene families undergoing expansion (blue) or contraction (red) events. (**b**) The distribution of single-copy, multiple-copy, unique, and other orthologs in the 16 mollusks. (**c**) Venn diagram represents the number of shared and unique gene families among five Panpulmonata species. (**d**) A simplified diagram showing the evolution of *A. vulgaris* water-land transition. (**e**) Gene Ontology (GO) enrichment map summarizing major biological networks of *Arion vulgaris* and Stylommatophora specific, expanded and contraction genes. Each node represents one GO term with adjusting P-value < 0.05. Node sizes indicate the number of genes within the corresponding GO term. The thickness of the edges represents the number of genes shared by two terms. Striking groups were manually circled and labeled.

The relationship of early gastropods has been controversial for a long time as different datasets and methodology show different topologies^31–33^. By means of comparing whole genomic data, a total of 223 single-copy orthologous genes (158,094 amino acid sites) were identified from 14 gastropod species that cover five main gastropod subclasses and 2 bivalve species (Supplementary Table S3). Both concatenated and coalescent-based methods produced an identical strongly supported topology (bootstrap value = 100, posterior probabilities = 1), except for the position of the Patellogastropoda and the Vetigastropoda + Neomphalina clades (Fig. 2a; Supplementary Fig. S6a). Our results show Patellogastropoda as sister to all other gastropods, and monophyletic Vetigastropoda + Neomphalina as sister to the clade Apogastropoda (Heterobranchia + Caenogastropoda) with relatively higher support compared with the other two topologies: Patellogastropoda as sister to Vetigastropoda + Neomphalina, and Patellogastropoda as sister to Heterobranchia + Caenogastropoda (Fig. 2a; Supplementary Fig. S6). The results thus favor the hypothesis of a clade Orthogastropoda (the united clade of Heterobranchia, Caenogastropoda, Vetigastropoda, and Neritimorpha), which is congruent with morphology-based and recently reported mitogenomic phylogenies^32,34–36^; but see Chen and Schrödl^37^.

Molecular dating suggests that *A. vulgaris* diverged from the most recent common ancestor with the land snails *Lissachatina (Achatina) fulica* and *Li. immaculata* about 126 million years ago (Mya, 95% confidence interval: 92–159 Mya) (Fig. 2a). The estimated divergence time is close to a previous estimate (132 Mya) based on mitochondrial genomes^38^. Stylommatophora split from Hygrophila around 199 Mya (95% confidence interval: 159–228 Mya), and Panpulmonata split from Sacoglossa around 235 Mya (95% confidence interval: 191–260 Mya) (Fig. 2a).

### Analysis of gene family evolution provides insights into *A. vulgaris* terrestrial adaptation

Recently, the changes of gene families have been recognized as a primary driver of phenotypic diversity and adaptive evolution^9^. Hence, we investigate the genetic basis of species adaptative evolution by defining the relationship of gene families. Based on pairwise sequence similarities, we identified 26,693 putative orthologous gene families composed of 378,381 genes among *A. vulgaris*, other gastropods, and outgroup species, of which 1610 gene clusters were shared by all gastropod species, representing ancestral gastropod gene families (Fig. 2b; Supplementary Table S7). A total of 10,311 orthologous gene families were shared by all Heterobranchia species and 7688 orthologous gene families were shared by five Panpulmonata species (Fig. 2b, c; Supplementary Table S7).

To explore the genetic basis of terrestrial adaptability shared by stylommatophoran species, we considered the properties of the 1126 gene families exclusively shared by three stylommatophoran species (Fig. 2c, d). GO enrichment analyses of these lineage-specific genes demonstrated that they were mainly assigned to kidney development, CTP/UDP metabolic processes, regulation of blood pressure, muscle growth, and spinal development (Fig. 2e; Supplementary Fig. S7; Supplementary Table S8). Molluscan kidneys are involved in the secretion of waste and the resorption of metabolites from the urinary fluid^39^. The enrichment of a series of genes related to kidney and ureteric bud development suggests the improvement of the efficiency of maintaining water balance and nutrients re-absorption in stylommatophoran species (Supplementary Table S8). In addition, the enriched biological process related to blood pressure regulation might be responsible for overcoming the gravity problem during landing^8^. Moreover, the enriched functions of muscle growth and spinal development might also improve the movement and flexibility in terrestrial life (Fig. 2e; Supplementary Fig. S7; Supplementary Table S8). There were 2140 and 1958 gene families expanded and contracted in the Stylommatophora lineage respectively (Fig. 2a). The expanded genes were functionally enriched in response to stimulus, response to radiation, signaling, larval development, and regulation of feeding/eating behavior (Fig. 2e; Supplementary Fig. S8; Supplementary Table S9). Meanwhile, genes related to transmembrane transport, fatty acid elongation, and centrosome cycle were contracted in both *A. vulgaris*, *Li. fulica* and *Li. immaculata* (Fig. 2e; Supplementary Fig. S9; Supplementary Table S10)*.* A total of 251 genes are likely positively selected in Stylommatophora, and their function mainly refers to the regulation of myelination (Supplementary Fig. S10; Supplementary Table S11).

Considering the specific adaptations of shell-less *A. vulgaris* (Fig. 2d), we identified a total of 2763 genes unique to *A. vulgaris*, of which 2629 (95.2%) have known InterPro domains (Supplementary Table S7). We found *A. vulgaris* specific genes were significantly enriched in functional categories related to isoprenoid metabolic process and organelle cell components (Fig. 2e; Supplementary Fig. S11; Supplementary Table S12). In comparison with two *Lissachatina* land snail species, *A. vulgaris* expanded genes exhibited significant enrichment in various aspects, including immune system, response to biotic/radiation stress, excretion, etc., which are very likely beneficial for its land adaptation (Fig. 2e; Supplementary Fig. S12; Supplementary Table S13). Specifically, genes related to response to corticosteroids and glucocorticoid pathways are highly increased. Corticosteroids are involved in a wide range of physiologic systems such as stress response, immune response, and regulation of inflammation^40^, glucocorticoids act primarily on carbohydrate and protein metabolism, and have anti-inflammatory effects^41,42^. Moreover, processes in acute inflammatory response and regeneration are also enriched. All of these might highly improve the ability of *A. vulgaris* to recover from damage. We found *A. vulgaris* expanded genes were also enriched in response to molecules of bacterial origin and response to lipopolysaccharide, which might improve its ability in response to biotic stress. The enrichment of pigment metabolism processes might advance the ability of shell-less *A. vulgaris* to reduce solar radiation damage. Furthermore, genes related to excretion, uronic acid metabolism, and larval development are expanded in *A. vulgaris*. Surprisingly, we also found an enrichment of genes related to pesticides, which might be the result of interaction with human agricultural activity. Similar to genes contracted in Stylommatophora, a high proportion of contracted genes were functionally related to transmembrane transport processes. In addition, contracted genes that regulate circadian rhythm and oxidase activity are also enriched (Fig. 2e; Supplementary Fig. S13; Supplementary Table S14). Strikingly, we found that genes involved in the positive regulation of interleukin-8 production were enriched in *A. vulgaris* contracted genes, and genes related to Interleukin-3,4,9,10,12,21,23,27,35 were likely positively selected in *A. vulgaris* (Supplementary Fig. S14; Supplementary Table S15). This adaptive immune response again might highly increase the ability of *A. vulgaris* in response to stress and stimuli.

### Whole-genome duplication events shared by Stylommatophora species

Whole-genome duplication (WGD) events are proposed to be a key evolutionary event driving phenotypic complexity, functional novelty, and ecological adaptation^43^. An earlier study suspected a WGD event somewhere at the base of Stylommatophora by comparison of chromosome numbers among closely related mollusks^44^, and a recent genomic study of *Li. immaculata* and *Li. fulica* proved the WGD event using genomic analysis and deduced the WGD event occurred around 70 Mya^11^. However, 70 Mya is much later than the divergence time that we estimated between *A. vulgaris* and *Lissachatina* (126 Mya, Fig. 2a). Therefore, we raised two questions: (a) whether *A. vulgaris* also experienced a WGD event, and (b) if it has happened, whether it happened independently after divergence from *Lissachatina* or it was shared by their common ancestor.

**Figure 3:**
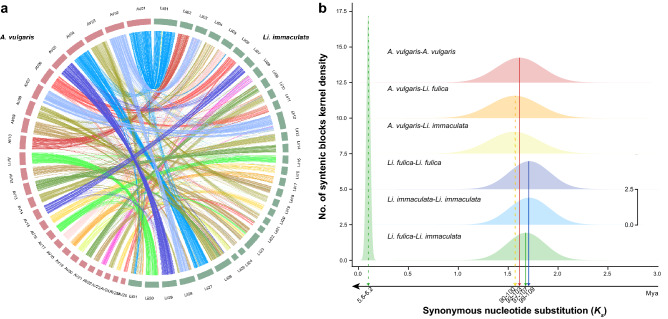
Whole-genome duplication shared by *Arion vulgaris*, *Lissachatina (Achatina) fulica,* and *Li. immaculata*. (**a**) An approximately one to one corresponding relationship in the comparison of *A. vulgaris* and *Li. immaculata* genomes. (**b**) Frequency distributions are shown of values of synonymous substitutions (*Ks*) for homologous gene pairs in comparisons of *A. vulgaris*, *Li. fulica* and *Li. immaculata*.

Our results of chromosome macrosynteny show that most of the chromosomes found a corresponding one in the *A. vulgaris* genome (Fig. 1b). In addition, we detected an approximately one-to-one corresponding relationship in the comparison of *A. vulgaris* (n = 26) and *Li. immaculata* (n = 31) chromosomes (Fig. 3a) and a one-to-two corresponding relationship in the comparison of *A. vulgaris* and *Aplysia californica* (n = 17) chromosomes (Supplementary Fig. S15). In both *Lissachatina* and *A. vulgaris*, the distribution of synonymous substitutions (*Ks*) shows a clear peak, which represents WGD events. There is also an overall slower synonymous substitution rate in *A. vulgaris* (max *Ks*: 1.61) than in *Li. fulica* (max *Ks*: 1.71) and *Li. immaculata* (max *Ks*: 1.71) (Fig. 3b; Supplementary Fig. S16). Based on our results and the previous karyotype research, we conclude that a WGD event did occur in the ancestry of *A. vulgaris*.

To figure out when the WGD event happened, we further compared the synteny gene pairs between *A. vulgaris* and two *Lissachatina* snails. First, the results showed the best BLASTP hits of homologous gene pairs are from interspecies comparisons instead of intraspecies comparisons (Supplementary Figs. S17a–19a), which implies that the WGD event seems to have occurred before the divergence of *A. vulgaris* and *Lissachatina*. Moreover, the distribution of *Ks* of *A. vulgaris*-*Li. immaculata* gene pairs and *A. vulgaris*-*Li. fulica* gene pairs show only one peak each, respectively. The *Ks* values corresponding to the peak are smaller between species (*A. vulgaris*-*Li. fulica*: 1.56; *A. vulgaris*-*Li. immaculata*: 1.57) than within *A. vulgaris* (*A. vulgaris*-*A. vulgaris*: 1.61) (Fig. 3b). This result could be explained by the species differentiation event occurring shortly after the WGD event. Such a short time is reflected in our results as the peak of species differentiation coinciding with the peak of the WGD event in the *Ks* distribution of *A. vulgaris*-*Li. fulica and A. vulgaris*-*Li. immaculata*, and the overall distribution has moved towards small *Ks* (Fig. 3b)*.* Assuming that the mutation rate of Mollusca is 1.645 × 10^–9^ per site per year^45^, we estimated the WGD event happened at approximately 93–109 Mya, and the species differentiation of *A. vulgaris* and *Lissachatina* occurred a very short time after the WGD, with molecular dating estimates for this at approximately 90–103 Mya.

After WGD, the two sets of chromosomes evolved differently with one set of chromosomes being more structurally stable and conserved compared to the other (Supplementary Figs. S17–19b), and this imbalance might provide a rich genomic resource for rapid evolution and adaptation^46^. Since the differentiation of Arionoidea and Achatinoidea is almost at the base of Stylommatophora differentiation^47^, we further speculate that all Stylommatophora species shared the common WGD event. The newly generated chromosome set provided abundant evolutionary resources in functional novelty and ecological adaptation, which may have led to the successful territorialization and diversity of Stylommatophora species.

### Evolution of gene duplication and adaptability

**Figure 4:**
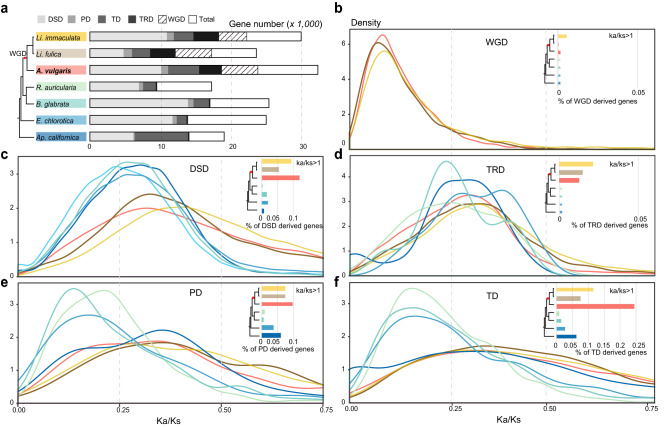
Gene duplication and evolution in Heterobranchia species. (**a**) The number of gene pairs derived from different modes of duplication. (**b–f**) The *Ka*/*Ks* ratio distributions of gene pairs derived from different modes of duplication in different species. Bar graphs show the proportion of positively selected genes (*Ka*/*Ks* > 1) that were duplicated by different mechanisms. Colors represent different species. WGD, whole-genome duplication; TD, tandem duplication; PD, proximal duplication; TRD, transposed duplication; DSD, dispersed duplication; Total, total annotated genes.

Gene duplication is another important evolutionary mechanism to provide new genetical material and opportunities to acquire new gene functions for an organism^48^. We found that Heterobranchia species have an abundance of duplicate genes. In our analysis, between 55% (*Elysia chlorotica*) and 75% (*Ap. californica*, *A. vulgaris*) of genes were identified as paralogous (Fig. 4a). Three Stylommatophora species (*A. vulgaris*, *Li. fulica,* and *Li. immaculata*) have an average of 16.8% more duplicate genes than other species. Among them, WGD events contribute 13% (*Li. immaculata*) to 22% (*Li. fulica*) to existing duplicates, and WGD- derived gene pairs are the most conserved among all types of duplicated genes (Fig. 4a, b; Supplementary Table S16). Another type of duplicate gene that has increased significantly in both Stylommatophora species is transposed duplication (TRD) gene, which is on average 48 times more frequent than in other species (Fig. 4a; Supplementary Table S16). Dispersed duplication (DSD) accounts for a high proportion (mean 56% of all duplicated genes, SD = 24%), while proximal duplication (PD) generates a small proportion (mean 5% of all duplicated genes, SD = 1.8%) of gene copies in all Heterobranchia species. Strikingly, tandem duplication (TD) gene pairs account for the highest proportion in *Ap. californica*, which is about 1.7–5.3 times that of other species (Fig. 4a; Supplementary Table S16).

The evolutionary pattern of duplicated genes is similar within three Stylommatophora species. The overall age of duplicated genes is young and under a weak purifying selection (*Ka*/*Ks* < 1) in both duplicated modes compared to other species (Fig. 4b–f; Supplementary Fig. S20). For *Ap. californica,* the TD- and PD-derived gene pairs have similar distribution of *Ka/Ks* (mode: TD-0.30; PD-0.35) between Stylommatophoran species (mean of the modes: TD-0.32; PD-0.36) when compared to other more closely related species (mean of the modes: TD-0.15; PD-0.17) suggesting that tandem and proximal duplicates happened recently and experienced relatively relaxed purifying selection (Fig. 4e, f). However, DSD- and TRD- derived gene pairs are more conserved in *Ap. californica*, which is more similar to *Radix auricularia*, *Biomphalaria glabrata*, and *E. chlorotica *(Fig. 4c, d; Supplementary Fig. S20)*.*

We further explored the roles of positive selection (*Ka*/*Ks* > 1) in the evolution of duplicated genes in seven Heterobranchia species. As expected, *A. vulgaris*, *Li. fulica* and *Li. immaculata* experienced stronger positive selection than other species, reflected by the high percentages of gene pairs showing *Ka*/*Ks* > 1 in all kinds of duplicated gene pairs (Fig. 4c, d; Supplementary Table S17). Among all duplicate genes, TD-, PD-, DSD- derived gene pairs have experienced stronger positive selection compared with genes generated by other duplication mechanisms (Fig. 4b–f; Supplementary Table S17). In *A. vulgaris*, 24% TD- derived genes were likely positively selected, which is 2–3 times that of *Li. immaculata* and *Li. fulica*, and 4–29 times that of other species (Fig. 4c, e, f; Supplementary Table S17). Interestingly, we found that the functional enrichment of genes caused by TD in *A. vulgaris* concerns response to external stress, pigment catabolism, and acute inflammatory process, which echoes the previous enrichment results of *A. vulgaris* unique and expanded genes and is related to its unique adaptation (Supplementary Fig. S21, 22). On the other hand, only 0.3% WGD—(which only exists in *A. vulgaris*, *Li. fulica,* and *Li. immaculata*), and 1.6% TRD—(which are highly expanded in *A. vulgaris*, *Li. fulica,* and *Li. immaculata*, Fig. 4a) derived gene pairs were likely positively selected in *A. vulgaris* (Fig. 4b, d; Supplementary Table S17). The TRD-derived gene pairs which were functionally enriched mostly refer to cell components (Supplementary Fig. S23), and WGD-derived genes were prone to be enriched in basic biological functions such as signal transduction, ion transport, muscle development (Supplementary Fig. S24).

### Massive expansion of transposable elements in *A. vulgaris* genome

Repeat content analysis showed that the repeat sequences occupy approximately 75.09% (1.15 Gb) of the *A. vulgaris* assembly (Supplementary Table S18), which is the highest value among all studied gastropod species^23^. We also found that species in Heterobranchia have a higher repeat content than other gastropod groups (i.e., Caenogastropoda, Vetigastropoda, Neomphalina, and Patellogastropoda) (Supplementary Fig. S25). In all types of repetitive sequences, transposable elements (TEs) account for 61.08% of the *A. vulgaris* assembly, and among them, long interspersed elements (LINEs), DNA transposons (DNAs), and short interspersed elements (SINEs) account for 36.39%, 5.44%, 1.78% of the assembly, respectively.

**Figure 5:**
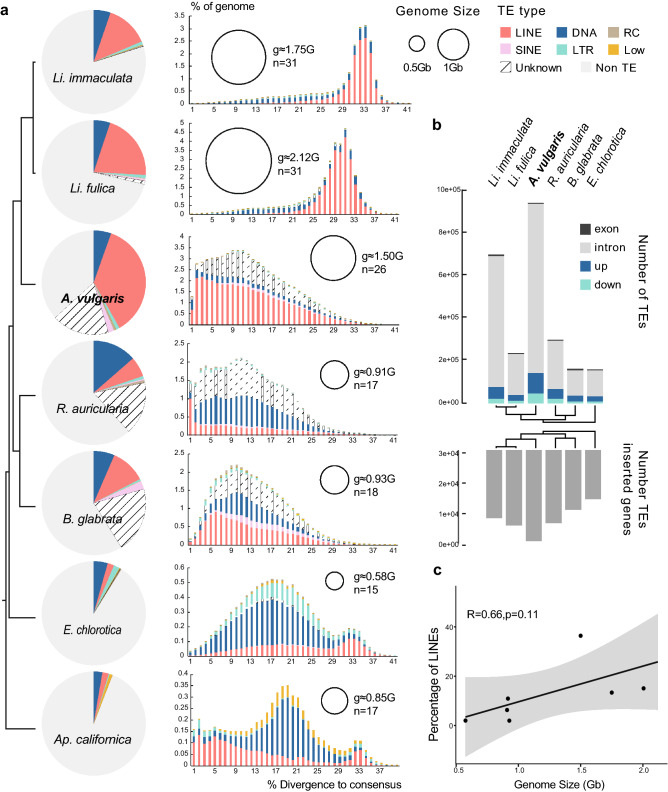
Massive expansion of transposable elements (TEs) in *Arion vulgaris* genome. (**a**) Left: TE composition by class among Heterobranchia species. Right: TE accumulation history corresponding to each species. The size of the circle indicates the estimated genome size of each species. n represents the number of haploid chromosomes; g represents the estimated genome size. (**b**) Bar graph shows the number of TEs in different genic regions and the number of TEs-inserted genes in each species. (**c**) Relationship between LINEs coverage and genome size in Heterobranchia species. Numbers on linear regressions correspond to adjusted r^2^ coefficients (Pearson’s test).

A high proportion of unclassified TEs (17.76%) was also detected in the *A. vulgaris* genome (Fig. 5a; Supplementary Table S18). Overall, the composition of TEs of *A. vulgaris* is similar to *Li. fulica* and *Li. immaculata*, in which LINEs are dominant, whereas in other Heterobranchia species DNA transposons are most abundant (except *B. glabrata,* see below). Most of the LINEs in *A. vulgaris* showed a low divergence rate, indicating a recent explosion of LINEs in the *A. vulgaris* genome (peak % divergence to consensus = 3). However, *Li. fulica* and *Li. immaculata* LINEs were not recent invaders since they exhibit a large divergence from the consensus (the distributions peak at 31% divergence for *Li. fulica* and 33% for *Li. immaculata*) (Fig. 5a). Two freshwater snails (*R. auricularia*, *B. glabrata*) and *Ap. californica* also showed recent expansion of LINEs, which even resulted in LINEs that replaced DNAs and became the dominant TE type in the *B. glabrata* genome (Fig. 5a). We found that although the total TE number of *A. vulgaris* is 1.35–6.09 times greater than in the other species considered, the insertion of TEs was very conservative. Specifically, genes with TEs distributed in putatively functional regions, i.e., 2 kb upstream, 1 kb downstream, or intron, exon regions in *A. vulgaris* were 1.21–1.87 times that of all other species. However, the number of TEs inserted into exons in *A. vulgaris* only accounts for 51% and 66% of that of *Li. fulica* and *B. glabrata*, respectively (Fig. 5b; Supplementary Table S19). Among all species, TEs were mainly inserted into introns in different degrees of divergence from consensus (Fig. 5b; Supplementary Fig. S26). The insertion of *A. vulgaris* TEs in intron regions greatly increased compared to other species (1.29–6.74 times), especially young TEs with a low divergence rate (%divergence to consensus < 16, Supplementary Fig. S26). The insertion into upstream and downstream is also increased, by 1.81–3.72 and 2.06–5.39 times that of other species, respectively. Previous reports have shown that TEs are powerful facilitators of rapid adaptation to novel environments^49–51^. The recent expansion of LINEs in *A. vulgaris* may also have played an important role in promoting potential plasticity and stress resistance correlated with its invasiveness and competitiveness.

Recent studies showed that TEs have driven massive changes in genome size^52–54^. In our results, although we found TE coverage is slightly positively correlated with genome size, the correlation is not significant (Supplementary Fig. S27a). In further analyses, we determined that these positive contributions all come from the LINEs (Fig. 5c, Supplementary Fig. S27b-d), but are still not significantly related to genome size. However, *A. vulgaris* and *Lissachatina* have larger genome sizes compared to other species, thus we assume that the changes in the Heterobranchia species genome size might be the result of the expansion of LINEs and the WGD event.

### Population dynamics of *A. vulgaris*

**Figure 6:**
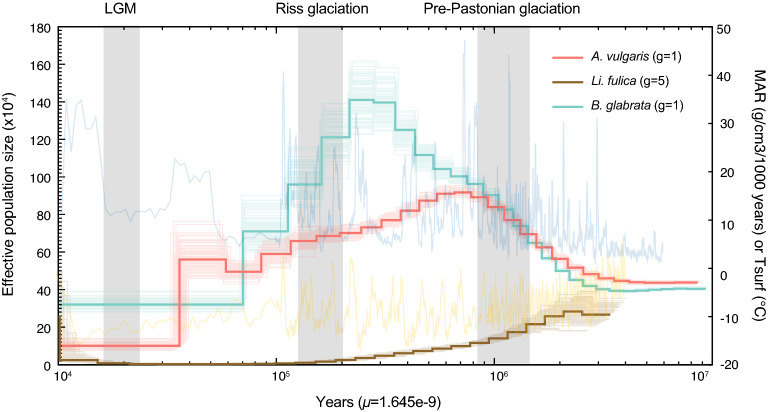
Demographic histories of *Arion vulgaris*, *Lissachatina (Achatina) fulica* and *Biomphalaria glabrata.* Solid bold lines indicate inferred ancestral effective population sizes for three species, while background blue and yellow lines represent the mass accumulation rate (MAR) and the atmospheric surface air temperature (Tsurf) relative to the present, respectively.

We observed an average genome-wide heterozygosity rate of 1.55 per hundred base pairs in *A. vulgaris*, which is about three times of the invasive land snail, *Li. fulica* (0.47 per hundred base pair)^55^, but comparable to freshwater snails *Pomacea canaliculata* (Caenogastropoda, 1.41%) and *P. maculate* (1.22%) which are also notable invaders^56^. We further compared the population dynamic history of *A. vulgaris* with relatively closely related invasive species *Li. fulica* and *B. glabrata*. We found that *A. vulgaris* and *B. glabrata* populations exhibited similar demographic histories, with a high *Ne* (4 × 10^5^) ~ 1.2 Mya and both increased between 1.2 and 0.8 Mya (Fig. 6). The *A. vulgaris* population continuously declined after the Pre-Pastonian glaciation and dramatically decreased ~ 40,000 years ago, which is consistent with the sharp temperature drop. *Li. fulica* population shows a relatively small *Ne* (3 × 10^5^) ~ 1.2Mya and a continuously prolonged decline until ~ 10,000 years ago, and then the population increased significantly to almost the initial level (Fig. 6). The very recent expansion of *Li. fulica* from a relatively small effective population size can also explain that the heterozygosity of *Li. fulica* is much smaller than that of *A. vulgaris*. On the other hand, the relatively long-term large effective population size of *A. vulgaris* may cause the complexity in its population structure, thereby increasing the difficulty of research on population expansion/invasion studies^22,57,58^.

## Discussion

Whole-genome duplication (WGD) is a common phenomenon in plants and has been shown in invertebrate species^59,60^. It plays an important role in providing evolutionary novelties and promoting speciation^43,61^. Based on chromosome-level genomic analysis of two land snails, Liu et al.^11^ first reported the WGD on the Sigmurethra-Orthurethra branch within Stylommatophora at ~ 70 Mya. However, our results indicate that the WGD is most likely an event shared by all Stylommatophora species, which we have dated back to 90–103 Mya (Fig. 3). The inconsistency in timing inference may be caused by the identification of paralogous gene pairs derived by the WGD event. In the study of Liu et al., MCScanX^62^ was used with default parameters to identify the collinearity blocks in *Li. immaculata* and *Li. fulica* and the *Ks* distribution was calculated using the gene pairs in the collinearity blocks. In our initial analysis, we used the same method as Liu et al., described. We did observe *Ks* peaks shared by *A. vulgaris* and *Li. fulica* which represents the WGD event, however, the *Ks* distribution of *Li. immaculata* has a relatively large deviation (Supplementary Fig. S28). From our results of the syntenic dot plots, *Li. immaculata* exhibits lower synteny in comparisons to *A. vulgaris* than the *Li. fulica*-*A. vulgaris* comparison (Supplementary Figs. S17, 18), implying that *Li. immaculata* has experienced more genome reconfiguration and chromosome rearrangement, and this may increase errors and difficulty in the identification of collinearity gene pairs within *Li. immaculata*. We addressed this problem by implementing WGDI^63^, a new tool which can identify collinearity more accurately and comprehensively. In the *Ks* distribution obtained by WGDI, the three species have relatively consistent *Ks* peaks (Fig. 3b). Therefore, we suppose that the estimation based on *Ks* distribution derived from WGDI can more accurately represent the older than expected time of the WGD event.

In our results, after WGD, the extra chromosome copy shows a release of selective pressure with large structural variations and increased synonymous mutation rate (Supplementary Figs. S17–19), which might serve as an abundant resource for mutations and novo functions, and may have facilitated the stylommatophoran transition from water to land. For example, expansion genes derived by WGD duplications are enriched in nerve and muscle development, which might enhance the locomotion and movement ability in terrestrial environments (Supplementary Fig. S24). Moreover, we also detected genes related to kidney development, response to stimulation, radiation, larval development, and dietary habits that were expanded in both *A. vulgaris* and shelled land pulmonates (Fig. 2d). These genes might have contributed to the stylommatophoran ancestor’s ability to overcome challenges such as gravitational pressure and water loss brought by the terrestrial environment.

The split of *A. vulgaris* and *Lissachatina* land snail lineages happened in a very short time after WGD (Fig. 3b). However, the slug *A. vulgaris* has evolved its unique adaptability in further improving water re-absorption and resistance to external stimuli (Fig. 2d). For example, a series of interleukin genes were positively selected in *A. vulgaris* genome, which might enhance the immune response; genes related to acute inflammatory processes were expanded, which might improve the innate defense; the expansion of genes related to regeneration might help to quickly recover from body/organ damage and increase the survival rate, and the expansion of genes and pathways in pigment biosynthesis might protect *A. vulgaris* from solar radiation.

In addition to WGD, small scale gene duplication also plays an important role in providing new genetic material for mutation, drift, and selection^61^. We found Heterobranchia species have an abundance of duplicated genes. Among all types of duplicated genes, the largest category is dispersed duplication (DSD) genes (Fig. 4). The proportion of tandem duplication (TD) genes has greatly increased in *A. vulgaris* as compared to other Heterobranchia species, and ~ 24% of them were positively selected (*Ka*/*Ks* > 1). Enrichment analysis showed the functions of TD derived genes largely overlapped with *A. vulgaris* expansion gene functions, e.g., response to external stress, pigment catabolism, acute inflammation, which thus implies that tandem duplication of genes might be one of the forces driving evolution, adaptation, and potential invasiveness of *A. vulgaris*.

Previous studies have shown that transposable element (TE) insertions play a critical role in rapid phenotypic variation and might help invasive species to successfully adapt to a novel environment^49^. In our results, the recent massive expansion of TEs (more precisely, LINEs) in *A. vulgaris* might act as potent insertional mutagens, greatly enhancing the adaptive success, invasiveness, and the ability to outcompete other land slugs.

All in all, our genomic analysis reveals the powerful potential of *A. vulgaris* for adaptation and evolution, which may explain why *A. vulgaris* is considered as an invasive species in central Europe. However, there is ongoing controversy about its native range and invasiveness. According to the record of first discovery in many European countries, it was believed that the slug originated on the Iberian Peninsula and expanded its range into central and eastern Europe over the last five decades^12^. However, the very similar external appearance with other closely related native large arionids as well as (potential) hybrid species between *A. vulgaris*, *A. ater,* and *A. rufus*^64–66^, might have caused the misidentification of *A. vulgaris*, obscured the specimen records, and made it difficult to trace its origin and monitoring the spread only by morphological identification^66,67^. Recent studies based on the genetic diversity patterns of mitochondrial and nuclear loci suggested that *A. vulgaris* is native in central Europe rather than alien/invasive while probably invasive in other parts of Europe^20–22^. Our *A. vulgaris* individual was collected in Munich, Southern Germany and has a relatively rich genetic diversity, which implies a large effective population size. This result seems to support the point of view that *A. vulgaris* is more likely native rather than invasive at least in south Germany (Fig. 6). However, more robust conclusions still require extensive sampling and more population data. Our high-quality *A. vulgaris* genome will promote future population studies from the use of single/multiple molecular markers to the use of whole genome-wide polymorphism and will help us to understand its origin, expansion, and potential invasiveness more comprehensively.

## Materials and methods

### Sample collection and sequencing

An adult *A. vulgaris* was collected in the garden of the Zoologische Staatssammlung München, Germany. Genomic DNA was extracted from the foot muscle tissue with MagAttract HMW DNA Kit and CTAB method^69^. Quality was checked using agarose gel electrophoresis. Four different sequencing technologies were used to obtain the genome sequence (Supplementary Table S1). First, one Illumina paired-end sequencing library was generated following the manufacturer’s standard protocol (Illumina) with an insert size of 350 bp. Also, high molecular weight DNA was separated and loaded onto the 10X Genomics Chromium microfluidics controller for barcoding and generated two 10X Genomics linked-read libraries with an insert size of 350 bp. Those reads not only provided the long-range positional information to assemble contigs into scaffolds but were also used for the genome survey analysis and final base-level genome sequence correction^70^. One Hi-C library digested with MboI and with an insert size of 350 bp was constructed for providing long-range information on the grouping and linear organization of sequences along entire chromosomes to assemble the scaffolds into chromosome-level scaffolds^71^. The Illumina paired-end sequencing library, 10X Genomics linked-read libraries, and Hi-C library were sequenced on an Illumina HiSeqX Ten platform (Illumina, San Diego, CA, USA) with 150 bp paired-end reads. The raw reads generated by Illumina HiSeqX Ten platform were all filtered with the following criteria: reads with adapters, reads with N bases more than 5%, and reads with more than 65% of low-quality bases (≤ seven) using Fastp v0.20.0^72^. Meanwhile, Nanopore libraries were prepared using SQK-LSK109 kit and sequenced in the platform Nanopore PromethION **(**Oxford Nanopore Technologies**)**. We performed a base calling of the raw Nanopore data with Guppy v2.2.3^73^.

Total RNA was extracted from the ‘head’ part of the sample which includes tentacles, mantle, inner head and anterior visceral organs, and foot and sequenced on an Illumina NovaSeq platform with paired-end 150 bp.

### Genome feature estimation and assembly

The genome size and heterozygosity were estimated by GenomeScope v1.0.0^74^ using the quality-controlled paired-end Illumina sequence data and linked reads. We combined reads generated using different sequencing platforms to generate a high-quality de novo genome assembly (Supplementary Table S2). Specifically, long reads, generated with the Nanopore PromethION platform, were assembled into contigs using the wtdbg2 v2.2 assembler^75^. The contigs were subsequently polished by ntEdit v1.3.1^76^ using Illumina short reads and linked reads. The resulting contigs were then connected into scaffolds by 10X Genomics linked-read data using Scaff10X v4.2^77^. Hi-C reads were mapped to the draft assembly and processed using the hicstuff v2.2.2 pipeline^78^ with the parameters --aligner bowtie2 --enzyme MboI --iterative --matfmt graal --quality-min 30 --size 0. We ran instaGRAAL v0.1.2^79^ on the resulting matrix and the draft assembly with parameters --level 5 --cycles 100 --coverage-std 1 --neighborhood 5 and the module instagraal-polish for refinement. After building the interaction map of the final scaffolds with hicstuff, we noticed an intra-chromosomal translocation on chromosome 9 which could have been due to a misassembly. In the subsequent analysis, we mapped all reads to the assembly ‘chromosome 9’ and identified two breakpoints (at site 21,000,000 and 27,600,000 respectively) based on the read’ depth and gene distribution. We corrected the orders manually and reconnected sequences with 10 N’s at the new junction sites.

### Gene prediction

Protein-coding genes were predicted using the following approaches: ab initio prediction, homology-based prediction, and transcriptome-based prediction. For ab initio prediction, RNA-seq reads were first aligned to the *A. vulgaris* genome sequence using STAR v2.7.2b^80^, then the read alignment information was merged and used for Braker2 v2.1.5^81^ gene prediction pipeline. For homology-based prediction, we selected six gastropods from closely to distantly related to *A. vulgaris*, namely *Li. fulica*, *B. glabrata*, *Ap. californica*, *E. chlorotica*, *P. canaliculata*, *Haliotis rufescens* (Supplementary Table S3). The protein sequences of the six species were downloaded from NCBI and aligned against the assembled genome with MMseqs v11.e1a1c^82^. These results were then combined into gene models separately with GeMoMa v1.3.1^83^ using mapped RNA-seq data for splice site identification. The resulting gene annotation sets were further filtered using the GeMoMa module GAF with default parameters. For the transcriptome-based prediction, RNA-seq data had been assembled using both de novo and genome-guided approaches with Trinity vr20140413p1^84^, and the gene predictions were carried out with PASA v2.0.2^85^. All gene annotations were combined with EVM v1.1.1^86^ (Supplementary Table S4). Partial genes and genes with a coding length of less than 150 bp were removed from further analysis.

The predicted genes were functionally annotated by aligning them to the eggNOG^27^, SWISS-PROT^28^, TrEMBL^28^, KEGG^30^, and InterPro^29^ databases using BLAST v2.2.31^87^ with a maximal e-value of 1e−5 and by aligning to the Pfam database using HMMer v3.0^88^. Gene Ontology (GO) terms (Gene Ontology, RRID:SCR 002811) were assigned to the genes using the BLAST2GO v2.5 pipeline^89^.

### Gene family cluster and terrestrial adaptation analysis

To resolve the early phylogeny of gastropods, we selected the species according to the following rules: (1) coverage of as many subclasses as possible; (2) the lineage diversity within each subclass should be covered; (3) in case of closely related species, those with high-quality genomes or better gene annotations were preferred. As a result, fourteen Gastropoda species were selected, including six Heterobranchia: *Li. fulica*, *Li. immaculata*, *B. glabrata*, *R. auricularia*, *Ap. californica*, *E. chlorotica;* four Caenogastropoda: *P. canaliculata*, *Marisa cornuarietis*, *Lanistes nyassanus*, *Conus consors*; one Vetigastropoda: *H. rufescens*; one Neomphalina: *Chrysomallon squamiferum*, and one Patellogastropoda: *Lottia gigantea*. Two bivalve species: *Argopecten purpuratus* and *Saccostrea glomerata* were selected as outgroups (Supplementary Table S3). Protein sequences were extracted from each species and an all-against-all comparison was performed using BLASTP v2.9.0^90^ with an e-value cut-off of 1e−5. OrthoFinder v2.4.0^91^ was used to cluster gene families.

Based on the clustered gene families, we explored the terrestrial adaptation of *A. vulgaris* from two aspects. One is the genetic basis of adaptability shared by stylommatophoran species relative to other aquatic or marine Heterobranchia species, another is the specific adaptations of shell-less *A. vulgaris* compared to two land snails, *Li. fulica* and *Li. immaculata*. For both cases, we tested lineage/species specific genes, expansion/contraction genes, and positively selected genes (PSGs) and performed Gene Ontology (GO) enrichment analysis.

We applied the CAFÉ v4.2.1^92^ program to examine gene family expansion and contraction across entire genomes with default parameters. To identify PSGs, OrthoFinder v2.4.0^91^ was used to cluster gene families from five Heterobranchia species: *Li. fulica*, *Li. immaculata*, *B. glabrata*, *R. auricularia*, *Ap. californica* (Supplementary Table S3). Single-copy orthologous genes were extracted based on the results of clustered gene families. MAFFT v7.455^93^ was used for multiple sequence alignments and converted to codon sequences by PAL2NAL v14^94^. Poorly aligned positions were removed with Gblocks v0.91b^95^ with parameters “-b2 = 85% alignment length -b3 = 6 -b4 = 10 -b5 = h -t = c”. The PSGs were identified by comparing the null model (fix_omega = 1) to the alternative model (fix:omega = 0) using codeml branch-site model in the PAML package^96^. The foreground branch was set to (1) the node of the most common ancestor of *A. vulgaris, A. vulgaris* and *Li. immaculata,* to identify putative PSGs shared by stylommatophoran species, and (2) *A. vulgaris,* for the detection of potential PSGs of *A. vulgaris*. Chi-square tests were performed for each pair and genes with a 5% significance level were selected as putative PSGs^96^. Cytoscape v3.8.2^97^ was used for visualizing molecular interaction networks and biological pathways.

### Phylogenetic analysis

Gene families with only one copy from each of 16 species were selected as single-copy genes and were concatenated and aligned by MUSCLE v3.8.1551^98^ with default parameters. The maximum likelihood (ML) trees were inferred using both RAxML v8.2.8^99^ with the GTR+Γ model and IQ-TREE v1.6.9^100^, which automatically selected the best-fit substitution model using ModelFinder^101^. For coalescent-based analysis, gene trees were first estimated using RAxML v8.2.8^99^ with 100 replicates from each single copy gene. The best tree was then selected as input to ASTRAL v5.6.1^102^ to infer the species tree with default parameters. Gene trees were visualized using DensiTree v2.01^103^.

Divergence time was computed using the MCMCTREE program implemented in the PAML v4.8^96^ package. For calibration, we used the soft bounds of Euopisthobranchia—Panpulmonata (divergence time between 190 and 270 MY)^33^, the fossil of Sublitoidea (418 MY) constraints on the node of Heterobranchia and Caenogastropoda^104^, and the fossil of *Fordilla troyensis* (530 MY) for the root^105,106^.

### Identification of whole-genome duplication event

For macrosynteny analysis, LASTZ v1061^107^ was used to perform whole-genome alignments between chromosome-level assemblies of *A. vulgaris*, *Li. immaculata*^11^ and *Ap. californica*^108^*.* The alignments were visualized by Circos v0.69-6^109^. For synteny analysis of homologous gene pairs, the protein sequences of *A. vulgaris*, *Li. fulica* and *Li. immaculata* were first searched against themselves and also between species using BLASTP v2.9.0^90^, then subjected to WGDI v0.5.1^63^ to determine syntenic blocks, estimate *Ks* values for each block and calculate *Ks* distributions of gene pairs in collinearity blocks. Curves were fitted using the Gaussian approximation function in the WGDI package.

### Identifying gene duplications

The different modes of gene duplication were identified using the DupGen_finder v1.07 pipeline^110^ using *P. canaliculata* as a reference^56^. Gene pairs were further filtered to remove overlaps between different duplicate modes. For each duplicated gene pair, we aligned their protein sequences using MAFFT v7.455^93^ and converted the protein alignment into a codon alignment using PAL2NAL v14^94^. Then, the resulting codon alignment was formatted into an AXT format and *Ka* (number of substitutions per nonsynonymous site) and *Ks* (number of substitutions per synonymous site) values were calculated by KaKs_Calculator v2.0^111^.

### Repeat prediction and expansions of transposable elements

TRF v4.09^112^ was used for tandem repeats identification with default parameters. Transposable elements (TEs) were annotated using a combination of ab initio and homology-based approaches. First, repeat elements were identified de novo using RepeatModeler v2.0.1^113^. The database predicted by RepeatModeler, the RepBase^114^ (RepBase-20170127) and the Dfam^115^ (Dfam_Consensus-20170127) libraries were then merged together and used as a custom library for RepeatMasker v4.0.7^113^ to identify repeats comprehensively. The repeat divergence rate was measured by the percentage of substitutions in the corresponding regions between annotated repeats and consensus sequences in the RepBase database. For species with incomplete TE annotations (e.g., *Li. fulica*, *R. auricularia*), we predicted their TEs using the same approaches as just described. We regarded genes with TEs inserted in introns, exons or with 2-kb upstream or 1 kb downstream of the terminal exons as likely to be affected by these insertions and compared the number of genes affected by TEs in different insertion regions.

### Genome heterozygosity and reconstruction of effective population size (*Ne*)

Heterozygosity was estimated by the following steps. First, the clean Illumina reads and linked reads were merged and mapped onto the *A. vulgaris* assembly by BWA-MEM v0.7.17-r1188^116^ with default parameters. The sequence alignment/map (SAM) file format was processed using SAMtools v1.9^117^, and Picard v2.23.3^118^ was used to mark duplicates. Finally, single nucleotide polymorphisms (SNPs) calling was implemented in GATK v4.1.6.0^119^ using default parameters, and several filtering steps were performed to reduce false positives, including (1) remove SNPs with more than two alleles; (2) remove SNPs with a quality score less than 30; (3) remove SNPs at or within 5 bp from any InDels; (4) remove sites with extremely low (less than one-third average depth) or extremely high (more than three-fold average depth) coverage.

We inferred the demographic history by applying the Pairwise Sequentially Markovian Coalescence model using PSMC v0.6.5-r67^120^ with the following parameters: -N25 -t15 -r5 -p ‘4 + 25 × 2 + 4 + 6’. This method reconstructs the history of changes in population size over time using the distribution of the most recent common ancestor (tMRCA) between two alleles in an individual. The generation time of *A. vulgaris* and *B. glabrata* was assumed to be 1 year^121,122^ and *Li. fulica* was assumed to be 5 years^123^.

### Ethics declarations

No specific permits were required for the described field studies, no specific permissions were required for these locations/activities, and the field studies did not involve endangered or protected species.

## Supplementary Information

Supplementary Information 1.

Supplementary Information 2.

## Data Availability

The *A. vulgaris* genome project of this study was deposited at the National Center for Biotechnology Information (NCBI) under BioProject number PRJNA680311. Genomic and transcriptome sequence reads was deposited in the SRA database with BioSample: SAMN16874494. The assembled genome had been deposited at GenBank with accession number: GCA_020796225.1. In addition, the genome annotation files had been submitted at the Figshare: https://doi.org/10.6084/m9.figshare.15022212.v1; https://doi.org/10.6084/m9.figshare.15022203.v1.
