# Supplementary material for: Pulmonate slug evolution is reflected in the de novo genome of Arion vulgaris Moquin-Tandon, 1855
Source: Sci Rep. 2022 Aug 20;12:14226. doi: 10.1038/s41598-022-18099-7 (PMC9392753; doi:10.1038/s41598-022-18099-7)
Supplement: Supplementary file 1 — Supplementary Information 1. [file 41598_2022_18099_MOESM1_ESM.docx]

**Supplementary data 1. Genome statistics for Mollusca genomes.** G: Gastropoda, B: Bivalvia, C: Cephalopoda. BUSCOs: Bench marking Universal Single-Copy Orthologs. The column of BUSCOs shows the percentage of: C: Complete BUSCOs; S: Complete Single-Copy BUSCOs, D: Complete Duplicated BUSCOs; F: Fragmented BUSCOs; M: Missing BUSCOs; n: Total BUSCO groups searched. Statistics date until December 2021.

| **Class** | **Species** | **Assembly  size (Mb)** | **Heterozy-gosity** | **Repeat content** | **Contig N50** | **Scaffold N50** | **BUSCOs** | **Reference** |
| --- | --- | --- | --- | --- | --- | --- | --- | --- |
| G | *Lissachatina* (*Achatina*) *fulica* | 1,855.89 | 0.47% | 71.00% | 721,038 | 59,589,303 | C:91.5%[S:84.6%,D:6.9%], F:2.5%,M:6.0%,n:978 | (Guo et al., 2019) |
| G | *Lissachatina* (*Achatina*) *immaculata* | 1,653.15 | 0.24% | 57.70% | 3,802,429 | 56,367,627 | C:92.0%[S:85.5%,D:6.5%], F:1.2%,M:6.8%,n:978 | (Liu et al., 2020) |
| G | *Alviniconcha marisindica* | 829.61 | - | - | 727,552 | - | - | HKUST, 2021 |
| G | *Anentome helena* | 1,720.19 | - | - | 56,088 | 2,075,175 | - | IRIDION GENOMES, 2020 |
| G | *Aplysia californica* | 927.31 | 0.33% | 30% | 9,586 | 917,541 | C:92.4%[S:91.9%,D:0.5%], F:1.9%,M:5.7%,n:978 | Broad Institute, 2013 |
| G | ***Arion vulgaris*** | 1,541.03 | 1.55% | 75.09% | 8,603,329 | 64,342,731 | C:90.6%[S:85.0%,D:5.6%], F:1.9%,M:7.5%,n:978 | This study |
| G | *Babylonia areolata* | 1,108.40 | - | - | 5,933 | 6,321 | - | Fisheries and Technical, Economic College, 2020 |
| G | *Batillaria attramentaria* | 717.57 | - | - | 1,290,776 | - | - | Ewha Womans University, 2021 |
| G | *Biomphalaria glabrata* | 916.38 | 0.22-0.69% | 44.80% | 7,298 | 48,059 | C:88.4%[S:86.5%,D:1.9%], F:4.9%,M:6.7%,n:978 | (Adema et al., 2017)  (Wethington et al., 2007) |
| G | *Candidula unifasciata* | 1,286.46 | 1.09% | 61.10% | 246,413 | 246,413 | C:92.4%[S:85.3%,D:7.1%], F:1.6%,M:6.0%,n:978 | (Chueca, Schell, & Pfenninger, 2021) |
| G | *Cepaea nemoralis* | 3,490.92 | 1.43% | 76.40% | 330,079 | 333,110 | C:87.2%[S:74.3%,D:12.9%], F:3.8%,M:9.0%,n:954 | (Saenko, Groenenberg, Davison, & Schilthuizen, 2021) |
| G | *Chrysomallon squamiferum* | 404.61 | 1.38% | 25% | 1,883,489 | 30,197,426 | C:96.6%,n:978 | (Sun et al., 2020) |
| G | *Colubraria reticulata* | 67.10 | - | - | 890 | - | - | University of Konstanz, 2016 |
| G | *Conus betulinus* | 3,430.83 | - | 38.56% | 171,480 | 232,607 | C:89.8%[S:78%;D:11.8%], F:3.2%,M:7.0%,n:978 | (Peng et al., 2021) |
| G | *Conus consors* | 2,049.32 | - | - | 749 | 1,128 | - | (Andreson et al., 2019) |
| G | *Conus tribblei* | 2,160.49 | - | - | 854 | 2,681 | - | (Barghi, Concepcion, Olivera, & Lluisma, 2016) |
| G | *Conus ventricosus* | 3,591.51 | 1.05–1.08% | 53.36% | - | 93,519,712 | C:84.9%[S:82%,D:2.9%], F:4.3%,M:10.8%,n:978 | (Pardos-Blas et al., 2021) |
| G | *Dracogyra subfuscus* | 1,160.00 | 0.50% | 50% | 4,083 | 5,907 | C:97.1%[S:96.2%,D:0.9%], F:0.6%,M:2.3%,n:5295 | (Lan et al., 2021) |
| G | *Elysia chlorotica* | 557.48 | 3.66% | 32.60% | 30,474 | 441,954 | C:94.7%[S:93.3,D:1.4], F:2.5%,M:2.8%,n:978 | (Cai et al., 2019) |
| G | *Elysia marginata* | 790.32 | - | 29% | 6,205 | 225,654 | C:91.1%[S:89.6%,D:1.5%], F:6.7%,M:2.2%,n:978 | (Maeda et al., 2021) |
| G | *Gigantopelta aegis* | 1,149.61 | 0.50% | 51% | 461,769 | 81,591,406 | C:94.0%,n:954 | (Lan et al., 2021) |
| G | *Haliotis discus* | 1,865.48 | - | 30.76% | 41,000 | 200,099 | C:73.8%[S:68.4%,D:5.4], F:14.6,M:11.6,n:891 | (Nam et al., 2017) |
| G | *Haliotis laevigata* | 1,762.66 | 0.68% | - | 3,353 | 81,233 | C:86.8%[S:84.6%,D:2.2%], F:8.7%,M:4.5%,n:978 | (Botwright et al., 2019) |
| G | *Haliotis rubra* | 1,378.27 | 1.27-1.44% | - | 1,177,711 | 1,227,833 | C:94.6%[S:91.6%;D:3%], F:1.6%,M:3.8%,n:978 | (Gan et al., 2019) |
| G | *Haliotis rufescens* | 1,779.96 | 0.95% | 33.06% | 283,651 | 1,895,871 | C:95.1%[S:88.7%;D:6.4%], F:1%,M:3.9%,n:978 | (Masonbrink et al., 2019) |
| G | *Lanistes nyassanus* | 509.78 | 0.60% | 28.87% | 25,785 | 317,839 | C:95.0%[S:93.5%;D:1.5%], F:1.2%,M:3.8%,n:978 | (Sun et al., 2019) |
| G | *Limacina bulimoides* | 2,901.93 | - | - | 893 | - | C:30.3%[S:26.8%;D:3.5%], F:29.9%,M:39.8%,n:978 | (Choo et al., 2020) |
| G | *Lottia gigantea* | 359.51 | - | 22.25% | 96,027 | 1,870,055 | C:96.0%[S:94.9%;D:1.1%], F:1.63%,M:2.35%,n:978 | (Simakov et al., 2013)  (Nam et al., 2017) |
| G | *Lymnaea stagnalis* | 833.23 | - | - | 5,751 | - | - | BANG, 2016 |
| G | *Marisa cornuarietis* | 535.45 | 0.08% | 30.82% | 4,359,112 | - | C:96.4%[S:94.8%;D:1.6%], F:0.6%,M:3.0%,n:978 | (Sun et al., 2019) |
| G | *Patella pellucida* | 750.48 | 2.14% | 49.65% | 9,229,741 | - | C:96.4%[S:93.3%;D:3.1%],  F:1.1%,M:2.5%,n:978 | Wellcome Sanger Institute, 2021(hifiasm.purging) |
| G | *Phorcus lineatus* | 996.33 | 1.06% | 29.38% | 4,947,473 | - | C:85.4%[S:84.6%;D:0.8%], F:4.5%,M:10.1%,n:5295 | Wellcome Sanger Institute, 2021(hifiasm.purging) |
| G | *Phymorhynchus buccinoides* | 2,114.59 | - | - | 336,037 | - | - | BGI, 2021 |
| G | *Physella acuta* | 764.48 | - | - | 1,333 | 1,358 | - | (Ebbs, Loker, & Brant, 2018) |
| G | *Plakobranchus ocellatus* | 927.89 | - | - | - | 1,453,842 | C:95.0%[S:93.1%;D:1.9%], F:3.6%,M:1.4%,n:978 | (Maeda et al., 2021) |
| G | *Pomacea canaliculata* | 447.67 | 1.41% | 20.53% | 81,153 | 32,644,854 | C:96.4%[S:95.1%,D:1.3%], F:0.7%,M:2.9%,n:978 | (Sun et al., 2019) |
| G | *Pomacea maculata* | 432.29 | 1.22% | 21.25% | 75,997 | 375,864 | C:96.2%[S:95.0%;D:1.2%], F:0.6%,M:3.2%,n:978 | (Sun et al., 2019) |
| G | *Radix auricularia* | 909.76 | 0.263%-0.939% | 70% | 24,354 | 578,730 | C:93.4%, F:1.2%,M:5.4%,n:843 | (Schell et al., 2017)  (Al-Waaly et al., 2018)  et al., 2018) |
| G | *Steromphala cineraria* | 1,462.73 | 3.55% | 55.73% | 4,992,915 | - | C:85.5%[S:78.3%,D:7.2%], F:5.1%,M:9.4%,n:5295 | Wellcome Sanger Institute, 2021(hifiasm.purging) |
| B | *Anadara broughtonii* | 884.57 | - | 46.10% | 1,797,717 | 44,995,656 | C:91.7%[S:87.2,D:4.5], F:1.1%,M:7.2%,n:978 | (Bai et al., 2019) |
| B | *Archivesica marissinica* | 1,545.00 | 0.41% | 42% | 79,144 | 74,312,544 | C:91.8%[S:90.1%,D:1.7%], F:1.7%,M:6.5%,n:978 | (Ip et al., 2021) |
| B | *Argopecten irradians concentricus* | 874.78 | - | 47.19% | 63,725 | 1,246,717 | C: 91% [S:87.1;D:3.9%],  F:5.5%, M:3.5%, n:843 | (Liu et al., 2020) |
| B | *Argopecten irradians irradians* | 835.60 | 0.90% | 46.63% | 78,654 | 1,533,165 | C:91% [S:86.7;D:4.3%],  F:6.6%, M:2.4%, n:843 | (Du, Song, et al., 2017) |
| B | *Bathymodiolus platifrons* | 1,658.19 | 1.24% | 47.90% | 12,602 | 343,341 | C:84.67%[S:83%,D:1.7%], F:10.4%,M:4.9%,n:978 | (Sun et al., 2017) |
| B | *Corbicula fluminea* | 1,520 | 2.41% | 69.66% | 521,060 | 70,620,000 | C:86.7%[S:73%,D:13.6%], F:1.49%,M:11.86%,n:5295 | (Zhang et al., 2021) |
| B | *Crassostrea ariakensis* | 662.90 | 1.30% | 54.24% | 5,932,265 | 66,336,742 | C:97.1%[S:96.2%;D:0.9%], F:0.6%,M:2.3%,n:5295 | (Wu et al., 2021) |
| B | *Crassostrea gigas* | 558.60 | 0.73% | 36.10% | 19,387 | 401,319 | C:84.6%[S:81.1%;D:3.5%], F:5.7%,M:9.7%,n:978 | (Zhang et al., 2012) |
| B | *Crassostrea gigas* | 647.89 | 3.20% | 43% | 1,813,842 | 58,462,999 | C:95.6%[S:94%;D:1.6%], n:978 | (Penaloza et al., 2021) |
| B | *Crassostrea hongkongensis* | 608.62 | 1.08% | 45% | 2,576,225 | 55,855,599 | C:95.8%, F:0.8%,M:3.4%,n:978 | (Peng et al., 2020) |
| B | *Crassostrea virginica* | 684.74 | - | - | 1,971,208 | 75,944,018 | C:94.5%, F:1.2%,M:4.3%,n:978 | McDonnell Genome Institute - Washington University School of Medicine, 2017 |
| B | *Cyclina sinensis* | 903.12 | 1.53% | 43.14% | 2,587,078 | 46,470,132 | C:92.7%[S:91.6%,D:1.1%], F:1.3%,M:6.0%,n:978 | (Wei et al., 2020) |
| B | *Dreissena polymorpha* | 1,798.01 | 2.13% | 47.43% | 1,111,654 | 117,515,028 | C:92.3%[S:88.5%,D:3.8%], n:978 | (McCartney et al., 2021) |
| B | *Dreissena rostriformis* | 1,241.70 | 2.40% | 31.88% | 45,905 | 131,390 | C:83.23%[S:80.2%,D:3.1%], F:11.66%,M:5.11%,n:978 | (Calcino et al., 2019) |
| B | *Gari tellinella* | 1,597.63 | - | - | 19,181,371 | 85,279,272 | - | Wellcome Sanger Institute, 2021 |
| B | *Limnoperna fortunei* | 1,673.22 | 2.30% | 33.40% | 32,203 | 309,123 | C:81.9%[S:78.6%,D:3.3%], F:7.4%,M:10.7%,n:978 | (Uliano-Silva et al., 2018) |
| B | *Lutraria rhynchaena* | 543.90 | 0.9%-1.6% | 29.40% | 2,143,760 | - | C:95.8%[S:94.3%,D:1.5%] F:0.8%,M:3.4%,n:978 | (Thai et al., 2019) |
| B | *Magallana hongkongensis* | 757.93 | - | 41.12% | 49,472 | 72,332,161 | C:94.6%,n:978 | (Li et al., 2020) |
| B | *Margaritifera margaritifera* | 2,472.07 | 0.127–0.105% | 59.07% | 16,891 | 288,726 | C:84.9%[S:83.8%,D:1.1%], F: 4.9%, M:10.2%,n:954 | (Gomes-Dos-Santos et al., 2021) |
| B | *Megalonaias nervosa* | 2,365.22 | 0.78% | 25.00% | 50,186 | 50,649 | C:83%[S:80.9%,D:2.1%], F:9%, M:8%, n:978 | (Rogers et al., 2021) |
| B | *Mercenaria mercenaria* | 1,777.63 | 1.34% | 49.11% | 1,779,571 | 91,379,220 | C:90.5% [S:88.4%,D:2.1%],  F:0.9%, M:8.6%, n:954 | (Song et al., 2021) |
| B | *Mizuhopecten yessoensis* | 987.69 | 1.04% | 38.87% | 6,859 | 803,631 | C:88.9%[S:88.2%;D:0.7%], F:1.5%,M:9.6%,n:5295 | (Wang et al., 2017) |
| B | *Modiolus philippinarum* | 2,629.65 | 2.02% | 62.00% | 19,700 | 100,161 | C:70.3%[S:67.0%;D:3.3%], F:17.3%,M:12.4%,n:978 | (Sun et al., 2019)  (Calcino et al., 2019) |
| B | *Mya arenaria* | 1,324.96 | 4.6%-6.6% | 35% | 10,552 | 14,639 | C:76%[S:63%;D:13%], F:7%,M:17%,n:978 | (Plachetzki, Pankey, MacManes, Lesser, & Walker, 2020) |
| B | *Mytilus coruscus* | 1,903.83 | 1.39% | 47.40% | 817,337 | 898,347 | C:89.4% [S:88.1%;D:1.3%], F:1.9%,M:8.7%,n:978 | (Yang et al., 2021) |
| B | *Mytilus edulis* | 1,903.83 | 1.64% | 36.35% | 664,188 | 898,347 | C:91.1%[S:77.5%;D:13.6%, F:5.35%, M:3.56%,n:954 | (Li et al., 2020) |
| B | *Mytilus galloprovincialis* | 1,282.21 | 1.73 ± 0.24% | 43.00% | 77,157 | 207,642 | C:69%[S:56%;D:13%], F:8.5%, M:22.5%,n:843 | (Wick, Judd, & Holt, 2019) |
| B | *Ostrea lurida* | 1,140.79 | - | - | 7,815 | 12,947 | - | University of Washington School of Aquatic and Fishery Sciences, 2020 |
| B | *Panopea generosa* | 942.35 | - | - | 14,495 | 57,743,597 | C:66.70% | University of Washington School of Aquatic and Fishery Sciences, 2020 |
| B | *Pecten maximus* | 918.31 | 1.71% | 26.95% | 1,258,799 | 44,824,366 | C:94.5%[S:91.2%;D:3.3%], F:1.0%,M:4.5%,n:978 | (Kenny et al., 2020) |
| B | *Perna viridis* | 731.87 | 0.63% | 19.57% | 3,015 | 4,106,954 | C:99.4%[S:98.3%;D:1.1%], F:0.4%,M:0.2%,n:978 | (Inoue et al., 2021) |
| B | *Pinctada fucata* | 815.30 | - | 43.44% | 1629 | 167,048 | C:91.10% | (Takeuchi et al., 2016) |
| B | *Pinctada fucata martensii* | 990.98 | 1.30% | 48.50% | 21,518 | 59,032,463 | C:81.7%[S:72.5%;D:9.2%], F:7.8%,M:10.5%,n:978 | (Du, Fan, et al., 2017) |
| B | *Pinna nobilis* | 586.48 | 1.00% | 36.20% | 5,822 | 7,576 | C:27%,  F:13%, M: 60%,n:978 | (Bunet et al., 2021) |
| B | *Potamilus streckersoni* | 1,776.76 | 0.57% | 51.03% | 2,032,685 | 2,051,244 | C: 94.6%[S:93.7%;D:0.9%], F:1.2%,M:4.2%,n:978 | (Smith, 2021) |
| B | *Ruditapes philippinarum* | 1,123.16 | 1.04% | 38.30% | 29,238 | 345,005 | C:92.2%[S:90.3%;D:1.9%], F:1.6%,M:6.2%,n:978 | (Yan et al., 2019) |
| B | *Saccostrea glomerata* | 788.12 | 0.51% | 45.03% | 39,800 | 804,232 | C:79.7%, F:13.5%,M:6.6%,n:843 | (Calcino et al., 2019) |
| B | *Sinonovacula constricta* | 1,220.85 | 1.53% | 53.12% | 976,936 | 65,929,677 | C:88.8%[S:85.1%;D:3.7%], F:4.0%,M:7.2%,n:978 | (Dong et al., 2020) |
| B | *Solen grandis* | 1,324.49 | - | - | 50,000 | 67,678,117 | - | Quanzhou Normal University, 2021 |
| B | *Tegillarca granosa* | 797.65 | 1.17% | 53.75% | 605,873 | 42,616,908 | C:93.3% [S:88.7%;D:4.6%], F:1.3%,M:5.4%,n:978 | (Bao et al., 2021) |
| B | *Venustaconcha ellipsiformis* | 1,590.01 | 0.60% | 37.81% | 2813 | 6657 | C:67.9%[S:66.7%;D:1.2%], F:21.2%,M:10.9%,n:978 | (Renaut et al., 2018) |
| C | *Architeuthis dux* | 3,155.39 | - | 49.10% | 9000 | 5,478,336 | C:88.5%[S:87.6%;D:0.9%], F:3.6%,M:7.9%,n:978 | (da Fonseca et al., 2020) |
| C | *Euprymna scolopes* | 5,280.01 | - | 50% | 3558 | 3,549,550 | C:72.94%,n:978 | (Belcaid et al., 2019) |
| C | *Hapalochlaena maculosa* | 4,009.60 | - | - | 1,130 | 931,835 | - | (Greve et al., 2017) |
| C | *Nautilus pompilius* | 729.02 | - | 25.26% | 1,094,646 | - | C:91.3%[S:89.3%;D:2.0%], F:1.02%,M:7.68%,n:978 | (Zhang et al., 2021) |
| C | *Octopus bimaculoides* | 2,338.19 | 0.08% | 45% | 5532 | 475,182 | C:86.50% | (Albertin et al., 2015) |
| C | *Octopus minor* | 5,090.35 | - | 44.43% | 41,584 | 196,941 | C:76.2%[S:64.2%;D:12%], F:8.4%,M:15.4%,n:978 | (Kim et al., 2018) |
| C | *Octopus sinensis* | 2,719.15 | 0.34% | 42.26% | 490,217 | 105,892,736 | C:80.2%[S:72.3%;D:7.9%]  F:9.9%,M:9.9,n:303 | (Li et al., 2020) |
| C | *Octopus vulgaris* | 1,772.96 | 1.10% | 50% | 3,040 | 265,914 | C:51.6%,  F:9%, M:39.4%,n:954 | (Zarrella et al., 2019) |
| C | *Sepia pharaonis* | 4,785.53 | 0.35% | 77.30% | 1,926,397 | - | C:79.5%[S:73.6%;D:5.9%], F:10.2%,M:10.3%,n:954 | (Song et al., 2021) |
| C | *Watasenia scintillans* | 649.18 | 4.9–5.9% | 19.20% | 1,283 | - | C:58.9% | (Yoshida et al., 2020) |

**Reference**

Adema, C. M. *et al*. Whole genome analysis of a schistosomiasis-transmitting freshwater snail. *Nat. Commun.* **8,** 15451 (2017).

Albertin, C. B. *et al.* The octopus genome and the evolution of cephalopod neural and morphological novelties. *Nature* **524,** 220-224 (2015).

Al-Waaly, A.B. *et al.* Genetic diversity in Radix species from the middle and south of Iraq based on simple sequence repeats. *Afr. j. biotechnol.* **17,** 1119-1128 (2018).

Andreson, R. *et al.* Gene content of the fish-hunting cone snail Conus consors. Preprint at https://doi.org/10.1101/590695 (2019).

Bai, C. *et al.* Chromosomal-level assembly of the blood clam, Scapharca (Anadara) broughtonii, using long sequence reads and Hi-C. *Gigascience* **8,** giz067 (2019)

Bao, Y. *et al.* Genomic insights into the origin and evolution of molluscan red-bloodedness in the blood clam Tegillarca granosa. *Mol. Biol. Evol.* **38,** 2351-2365 (2021).

Barghi, N., Concepcion, G. P., Olivera, B. M., & Lluisma, A. O. Structural features of conopeptide genes inferred from partial sequences of the Conus tribblei genome. *Mol. Genet. Genom.* **291,** 411-422 (2016).

Belcaid, M. *et al.* Symbiotic organs shaped by distinct modes of genome evolution in cephalopods. *Proc. Natl. Acad. Sci. U.S.A.* **116,** 3030-3035 (2019).

Botwright, N. A. *et al.* Greenlip abalone (Haliotis laevigata) genome and protein analysis provides insights into maturation and spawning. *G3-GENES GENOM GENET* **9,** 3067-3078 (2019).

Bunet, R. *et al.* First insight into the whole genome shotgun sequence of the endangered noble pen shell Pinna nobilis: a giant bivalve undergoing a mass mortality event. *J. Molluscan Stud.* **87,** eyaa041 (2021).

Cai, H. *et al.* A draft genome assembly of the solar-powered sea slug Elysia chlorotica. *Sci. Data* **6,** 190022 (2019).

Calcino, A. D. *et al.* The quagga mussel genome and the evolution of freshwater tolerance. *DNA Res.* **26,** 411-422 (2019).

Choo, L. Q. *et al*. Novel genomic resources for shelled pteropods: a draft genome and target capture probes for Limacina bulimoides, tested for cross-species relevance. *BMC Genomics,* **21,** 11 (2020).

Chueca, L. J., Schell, T., & Pfenninger, M. De novo genome assembly of the land snail Candidula unifasciata (Mollusca: Gastropoda). *G3-GENES GENOM GENET* **11** (2021).

da Fonseca, R. R. *et al.* A draft genome sequence of the elusive giant squid, Architeuthis dux. *Gigascience,* **9,** giz152 (2020).

Dong, Y. *et al.* The chromosome-level genome assembly and comprehensive transcriptomes of the razor clam (Sinonovacula constricta). *Front. Genet.* **11,** 664 (2020).

Du, X. *et al.* The pearl oyster Pinctada fucata martensii genome and multi-omic analyses provide insights into biomineralization. *Gigascience,* **6,** 1-12 (2017).

Du, X., Song, K., Wang, J., Cong, R., Li, L., & Zhang, G. Draft genome and SNPs associated with carotenoid accumulation in adductor muscles of bay scallop (Argopecten irradians). *J. Genom.* **5,** 83-90 (2017).

Ebbs, E. T., Loker, E. S., & Brant, S. V. Phylogeography and genetics of the globally invasive snail Physa acuta Draparnaud 1805, and its potential to serve as an intermediate host to larval digenetic trematodes. *Evol. Biol.* **18,** 103 (2018).

Gan, H. M. *et al.* Best foot forward: nanopore long reads, hybrid meta-assembly, and haplotig purging optimizes the first genome assembly for the southern hemisphere blacklip abalone (Haliotis rubra). *Front. Genet.* **10,** 889 (2019).

Gomes-Dos-Santos, A. *et al.* The crown pearl: a draft genome assembly of the European freshwater pearl mussel Margaritifera margaritifera (Linnaeus, 1758). *DNA Res.* **28** (2021).

Greve, C. *et al.* Snails in the desert: species diversification of Theba (Gastropoda: Helicidae) along the Atlantic coast of NW Africa. *Ecol. Evol.* **7,** 5524-5538 (2017).

Guo, Y. *et al.* A chromosomal-level genome assembly for the giant African snail Achatina fulica. *Gigascience* **8,** giz124 (2019).

Inoue, K. *et al.* Genomics and transcriptomics of the green mussel explain the durability of its byssus. *Sci. Rep.* **11,** 5992 (2021).

Ip, J. C. *et al.* Host-endosymbiont genome integration in a deep-sea chemosymbiotic clam. *Mol. Biol. Evol.* **38,** 502-518 (2021).

Kenny, N. J. *et al.* The gene-rich genome of the scallop Pecten maximus. *Gigascience, 9*, giaa037 (2020).

Kim, B. M. *et al.* The genome of common long-arm octopus Octopus minor. *Gigascience,* **7,** giy119 (2018).

Lan, Y. *et al.* Hologenome analysis reveals dual symbiosis in the deep-sea hydrothermal vent snail Gigantopelta aegis. *Nat. Commun.* **12,** 1165 (2021).

Li, F. *et al.* Chromosome-level genome assembly of the East Asian common octopus (Octopus sinensis) using PacBio sequencing and Hi-C technology. *Mol. Ecol. Res.* **20,** 1572-1582 (2020).

Li, R. *et al.* The whole-genome sequencing and hybrid assembly of mytilus coruscus. *Front. Genet.* **11,** 440 (2020).

Li, Y. *et al.* Reconstruction of ancient homeobox gene linkages inferred from a new high-quality assembly of the Hong Kong oyster (Magallana hongkongensis) genome. *BMC Genomics* **21,** 713 (2020).

Liu, C. *et al.* Giant African snail genomes provide insights into molluscan whole-genome duplication and aquatic-terrestrial transition. *Mol. Ecol. Res.* **21,** 478-494 (2020).

Liu, X. *et al.* Draft genomes of two Atlantic bay scallop subspecies Argopecten irradians irradians and A. i. concentricus. *Sci. Data* **7,** 99 (2020).

Maeda, T. *et al.* Chloroplast acquisition without the gene transfer in kleptoplastic sea slugs, Plakobranchus ocellatus. *Elife,* **10** (2021).

Masonbrink, R. E. *et al.* An annotated genome for Haliotis rufescens (red abalone) and resequenced green, pink, pinto, black, and white abalone species. *Genome Biol. Evol.* **11,** 431-438 (2019).

McCartney, M. A. *et al.* The Genome of the zebra mussel, Dreissena polymorpha: a resource for comparative genomics, invasion genetics, and biocontrol. *G3-GENES GENOM GENET* **12** (2021).

Nam, B. H. *et al.* Genome sequence of pacific abalone (Haliotis discus hannai): the first draft genome in family Haliotidae. *Gigascience* **6,** 1-8 (2017).

Pardos-Blas, J. R. *et al.* The genome of the venomous snail Lautoconus ventricosus sheds light on the origin of conotoxin diversity. *Gigascience* **10,** giab037 (2021).

Penaloza, C. *et al.* A chromosome-level genome assembly for the Pacific oyster Crassostrea gigas. *Gigascience* **10,** giab020 (2021).

Peng, C. *et al.* The first Conus genome assembly reveals a primary genetic central dogma of conopeptides in C. betulinus. *Cell Discov.* **7,** 11 (2021).

Peng, J. *et al.* Chromosome-level analysis of the Crassostrea hongkongensis genome reveals extensive duplication of immune-related genes in bivalves. *Mol. Ecol. Res.* **20,** 980-994 (2020).

Plachetzki, D. C. *et al.* The genome of the softshell clam Mya arenaria and the evolution of apoptosis. *Genome Biol. Evol.* **12,** 1681-1693 (2020).

Renaut, S. *et al.* Genome survey of the freshwater mussel Venustaconcha ellipsiformis (Bivalvia: Unionida) using a hybrid de novo assembly approach. *Genome Biol. Evol.* **10,** 1637-1646 (2018).

Rogers, R. L. *et al.* Gene family amplification facilitates adaptation in freshwater unionid bivalve Megalonaias nervosa. *Mol. Ecol.* **30,** 1155-1173 (2021).

Saenko, S. V., Groenenberg, D. S. J., Davison, A., & Schilthuizen, M. The draft genome sequence of the grove snail Cepaea nemoralis. *G3-GENES GENOM GENET* **11** (2021).

Schell, T. *et al.* An annotated draft genome for Radix auricularia (Gastropoda, Mollusca). *Genome Biol. Evol.* **9,** 585–592 (2017).

Simakov, O. *et al.* Insights into bilaterian evolution from three spiralian genomes. *Nature* **493,** 526-531 (2013).

Smith, C. H. A high-quality reference genome for a parasitic bivalve with doubly uniparental inheritance (Bivalvia: Unionida). *Genome Biol. Evol.* **13,** evab029 (2021).

Song, H. *et al.* The hard clam genome reveals massive expansion and diversification of inhibitors of apoptosis in Bivalvia. *BMC Biology* **19,** 15 (2021).

Song, W. *et al.* Pharaoh cuttlefish, Sepia pharaonis, genome reveals unique reflectin camouflage gene set. *Front. Mar. Sci.* **8** (2021).

Sun, J. *et al.* The Scaly-foot Snail genome and implications for the origins of biomineralised armour. *Nat. Commun.* **11,** 1657 (2020).

Sun, J. *et al.* Signatures of divergence, invasiveness, and terrestrialization revealed by four apple snail genomes. *Mol. Biol. Evol.* **36,** 1507-1520 (2019).

Sun, J. *et al.* Adaptation to deep-sea chemosynthetic environments as revealed by mussel genomes. *Nat. Ecol. Evol.* **1,** 121 (2017).

Takeuchi, T. *et al.* Bivalve-specific gene expansion in the pearl oyster genome: implications of adaptation to a sessile lifestyle. *Zoological Lett.* **2,** 3 (2016).

Thai, B. T. *et al.* Whole genome sssembly of the snout otter clam, Lutraria rhynchaena, using nanopore and illumina data, benchmarked against bivalve genome assemblies. *Front. Genet.* **10,** 1158 (2019).

Uliano-Silva, M. *et al.* A hybrid-hierarchical genome assembly strategy to sequence the invasive golden mussel, Limnoperna fortunei. *Gigascience* **7,** gix128 (2018).

Wang, S. *et al.* Scallop genome provides insights into evolution of bilaterian karyotype and development. *Nat. Ecol. Evol.* **1,** 120 (2017).

Wei, M. *et al.* Chromosome-level clam genome helps elucidate the molecular basis of adaptation to a buried lifestyle. *iScience,* **23,** 101148 (2020).

Wethington, A. R. *et al.* Population genetic structure of Biomphalaria glabrata in a schistosomiasis-endemic region in Brazil. *J. Molluscan Stud.* **73,** 45-52 (2007).

Wick, R. R., Judd, L. M., & Holt, K. E. Performance of neural network basecalling tools for Oxford Nanopore sequencing. *Genome Biol.* **20,** 129 (2019).

Wu, B. *et al.* Chromosome-level genome and population genomic analysis provide insights into the evolution and environmental adaptation of Jinjiang oyster Crassostrea ariakensis. *Mol. Ecol. Res.* **00,** 1-16 (2021).

Yan, X. *et al.* Clam genome sequence clarifies the molecular basis of its benthic adaptation and extraordinary shell color diversity. *iScience* **19,** 1225-1237 (2019).

Yang, J. L. *et al.* Chromosome-level genome assembly of the hard-shelled mussel Mytilus coruscus, a widely distributed species from the temperate areas of East Asia. *Gigascience* **10,** giab024 (2021).

Yoshida, M. A. *et al.* Genomic and transcriptomic analyses of bioluminescence genes in the enope squid Watasenia scintillans. *Mar. Biotechnol.* **22,** 760-771 (2020).

Zarrella, I. *et al.* The survey and reference assisted assembly of the Octopus vulgaris genome. *Sci. Data* **6,** 13 (2019).

Zhang, G. *et al.* The oyster genome reveals stress adaptation and complexity of shell formation. *Nature* **490,** 49-54 (2012).

Zhang, T. *et al.* Dissecting the chromosome-level genome of the Asian Clam (Corbicula fluminea). *Sci. Rep.* **11,** 15021 (2021).

Zhang, Y. *et al.* The genome of Nautilus pompilius illuminates eye evolution and biomineralization. *Nat. Ecol. Evol.* **5,** 927-938 (2021).
