# Supplementary material for: Pulmonate slug evolution is reflected in the de novo genome of Arion vulgaris Moquin-Tandon, 1855
Source: Sci Rep. 2022 Aug 20;12:14226. doi: 10.1038/s41598-022-18099-7 (PMC9392753; doi:10.1038/s41598-022-18099-7)
Supplement: Supplementary file 2 — Supplementary Information 2. [file 41598_2022_18099_MOESM2_ESM.docx]

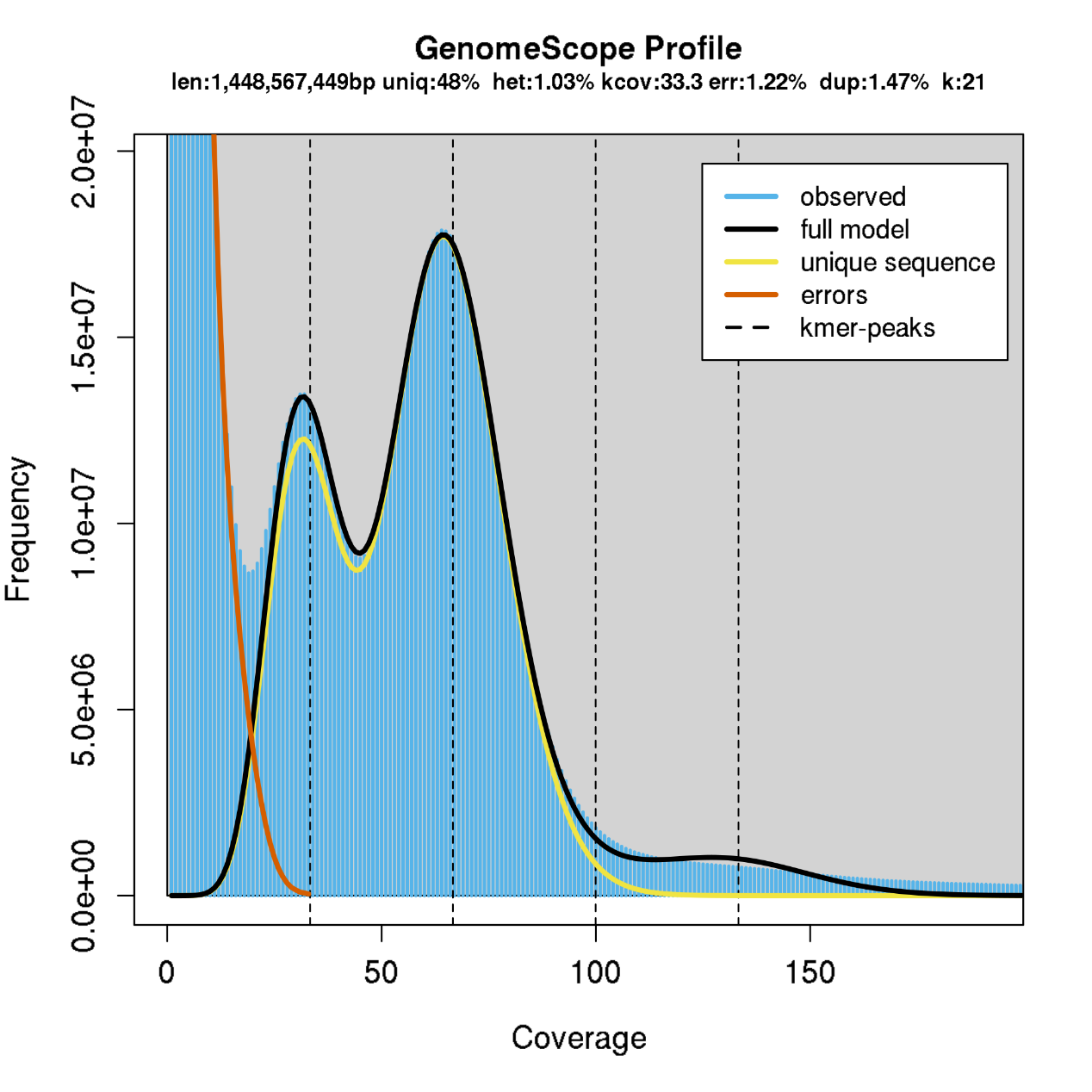


**Fig S1. Estimation of genome size of *Arion vulgari*s based on the distribution of 21-mer frequency in the combination of short reads and linked reads.** Two peaks indicate high heterozygosity.

**Fig S2. Hi-C chromosome contact maps.** Each block represents a Hi-C contact between two genomic loci within a 1 Mb window. The darker the color of a block, the higher the contact intensity. **a) An initial Hi-C contact map shows incongruous signals in chromosome 9, which might reflect a misassembly.** **b)** **Hi-C contact map after manual correction of the chromosome 9 error in the assembly.**

**Fig S3. Read *k-mer* frequency versus assembly copy number stacked histograms for final *Arion vulgaris* assembly.** Read content in black is absent from the assembly, red occurs once, purple twice, etc.


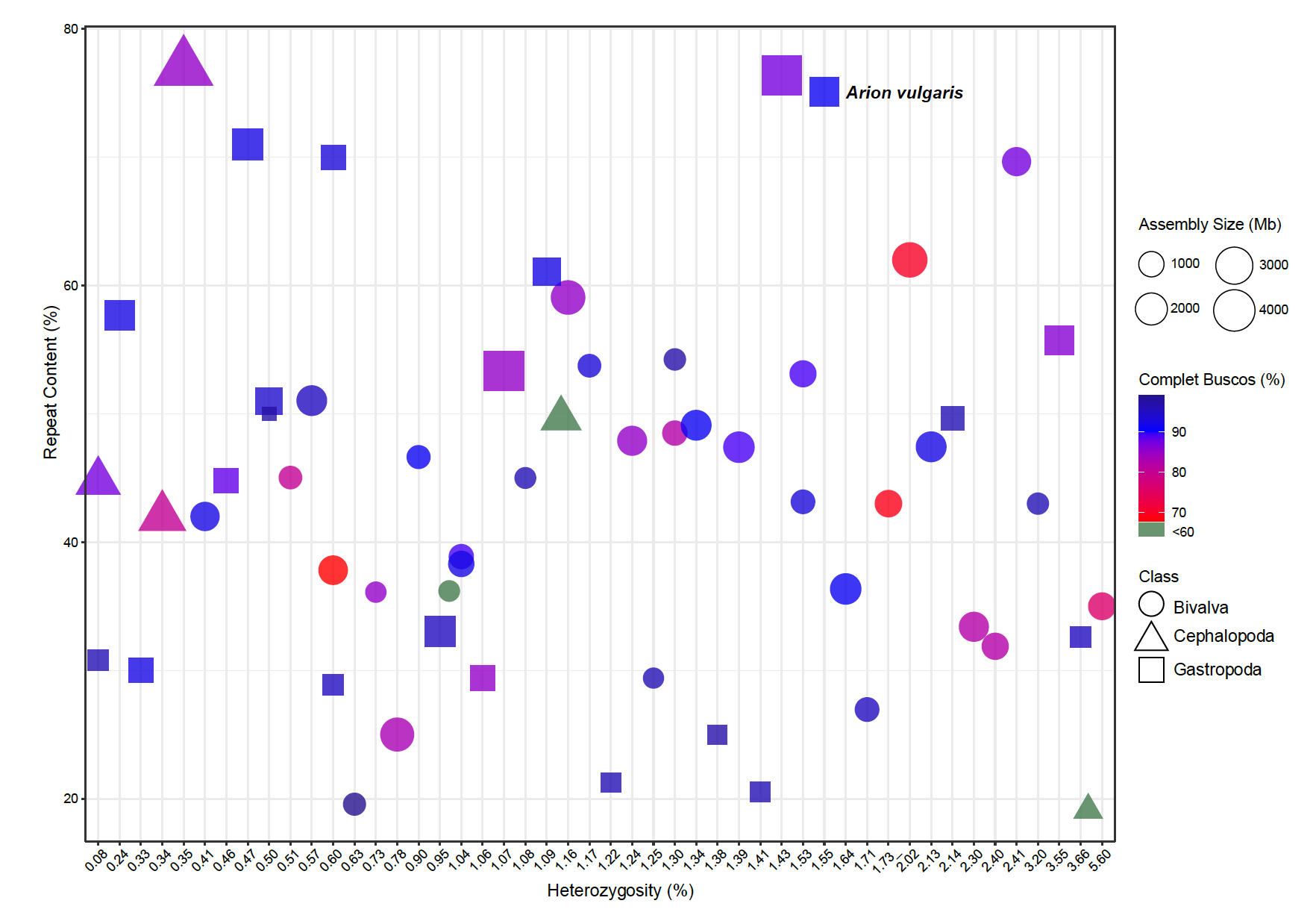


**Fig S4. Genome features and completeness of Mollusca genomes.** The figure includes all published mollusk genomes (statistics date until December 2021) with reported heterozygosity, repeat contents, assembled genome size and BUSCOs assessments. Each icon represents one genome, and the area of the icon represents the size of the assembled genome. Color shows the percentage of complete BUSCOs. Different classes are represented by different shapes. The *Arion vulgaris* genome is annotated in the Figure.


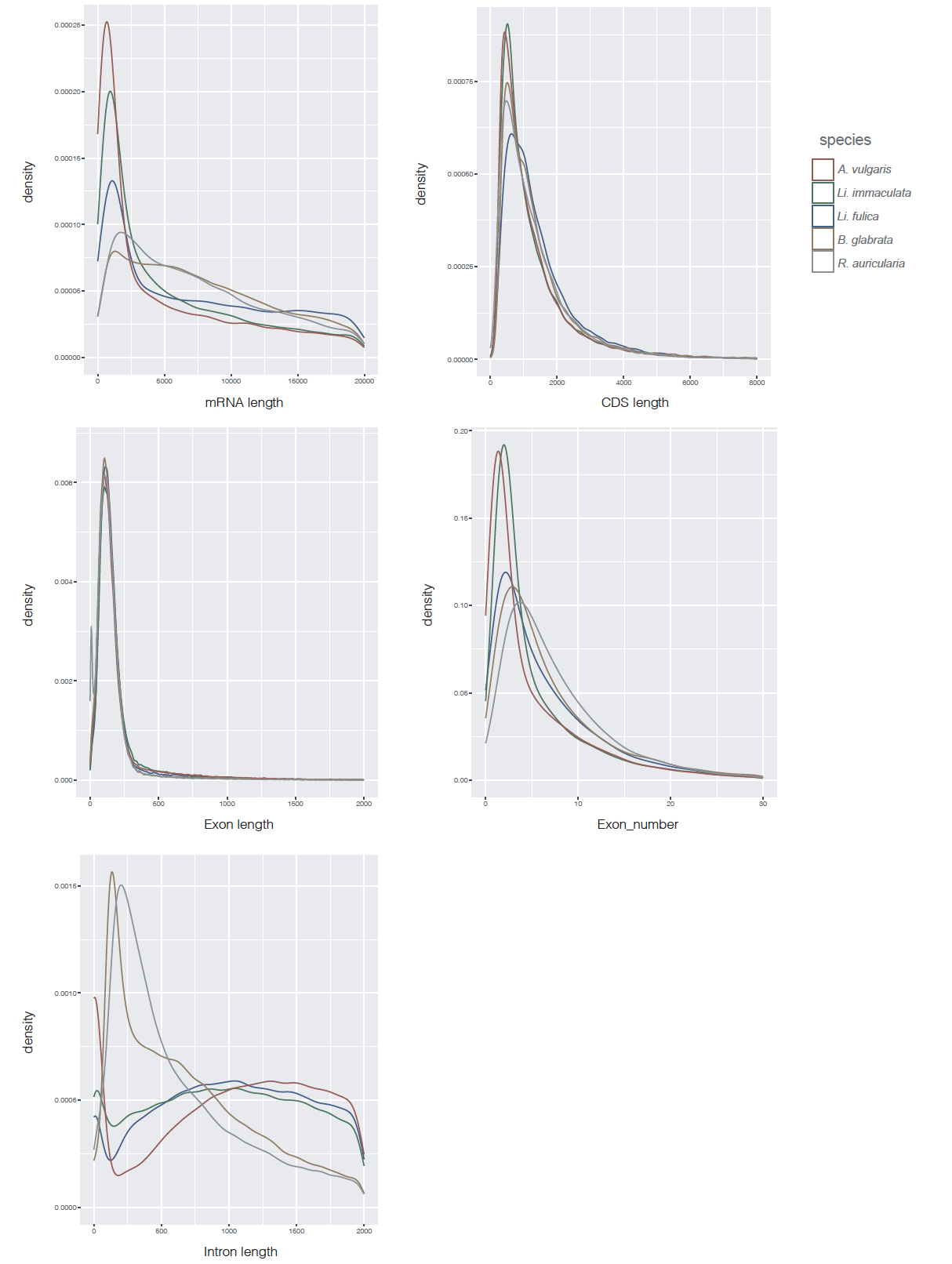


**Fig S5. Comparison of mRNA length (a), CDS length (b), exon length (c), exon number per gene (d), and intron length (e), between *Arion vulgaris*, *Lissachatina* (*Achatina) fulica, Li. immaculata, Biomphalaria glabrata,* and *Radix auricularia.***


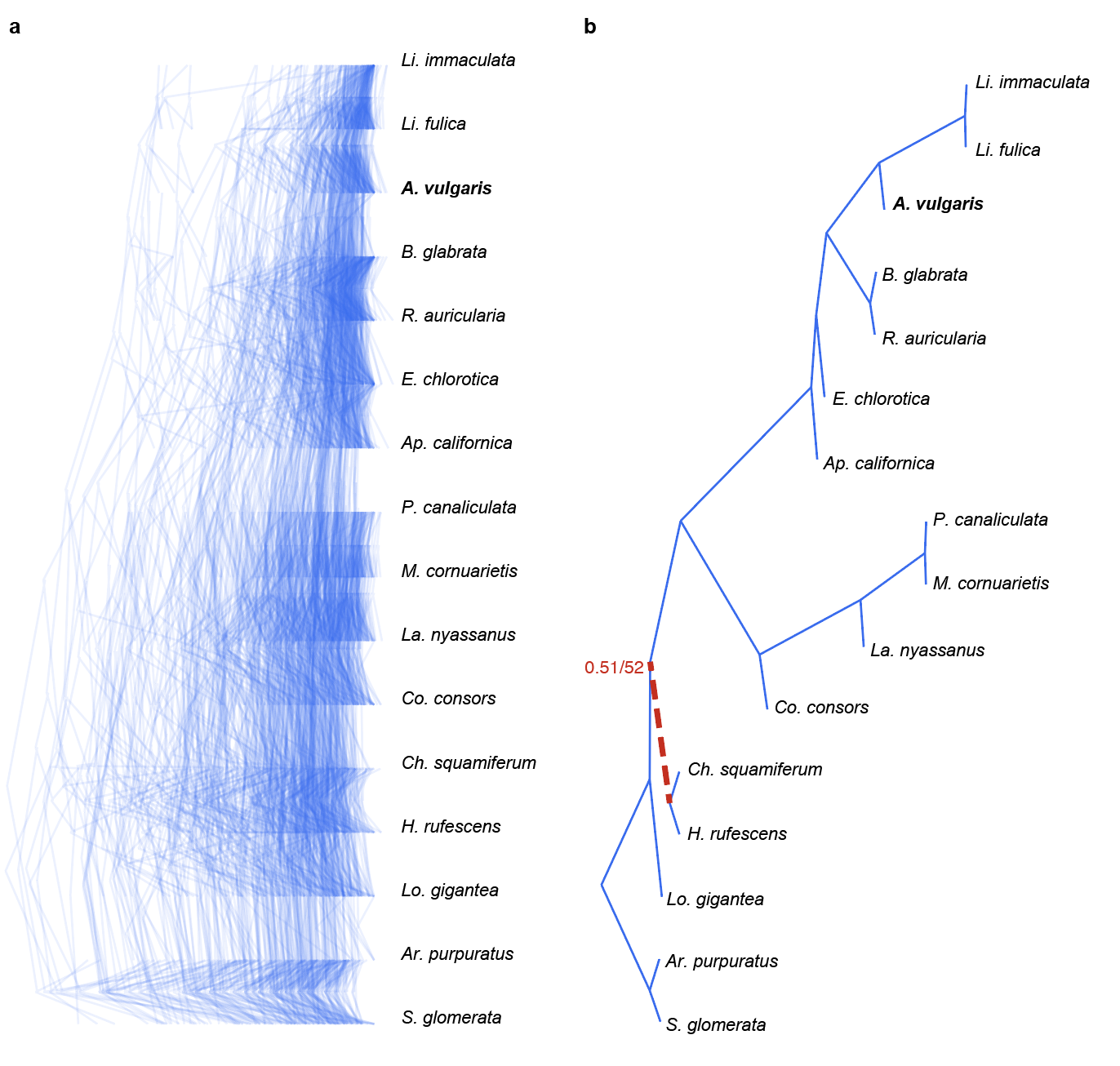


**Fig S6. Gene trees estimated using 223 orthologous genes. (a) Superimposed ultrametricity gene trees in a consensus DensiTree plot. (b) Final ASTRAL tree inferred by gene trees.** The sole branch without 100% bootstrap support is highlighted by a red dashed line and posterior probabilities and bootstrap support are indicated respectively. *Arion vulgaris* was highlighted in bold.


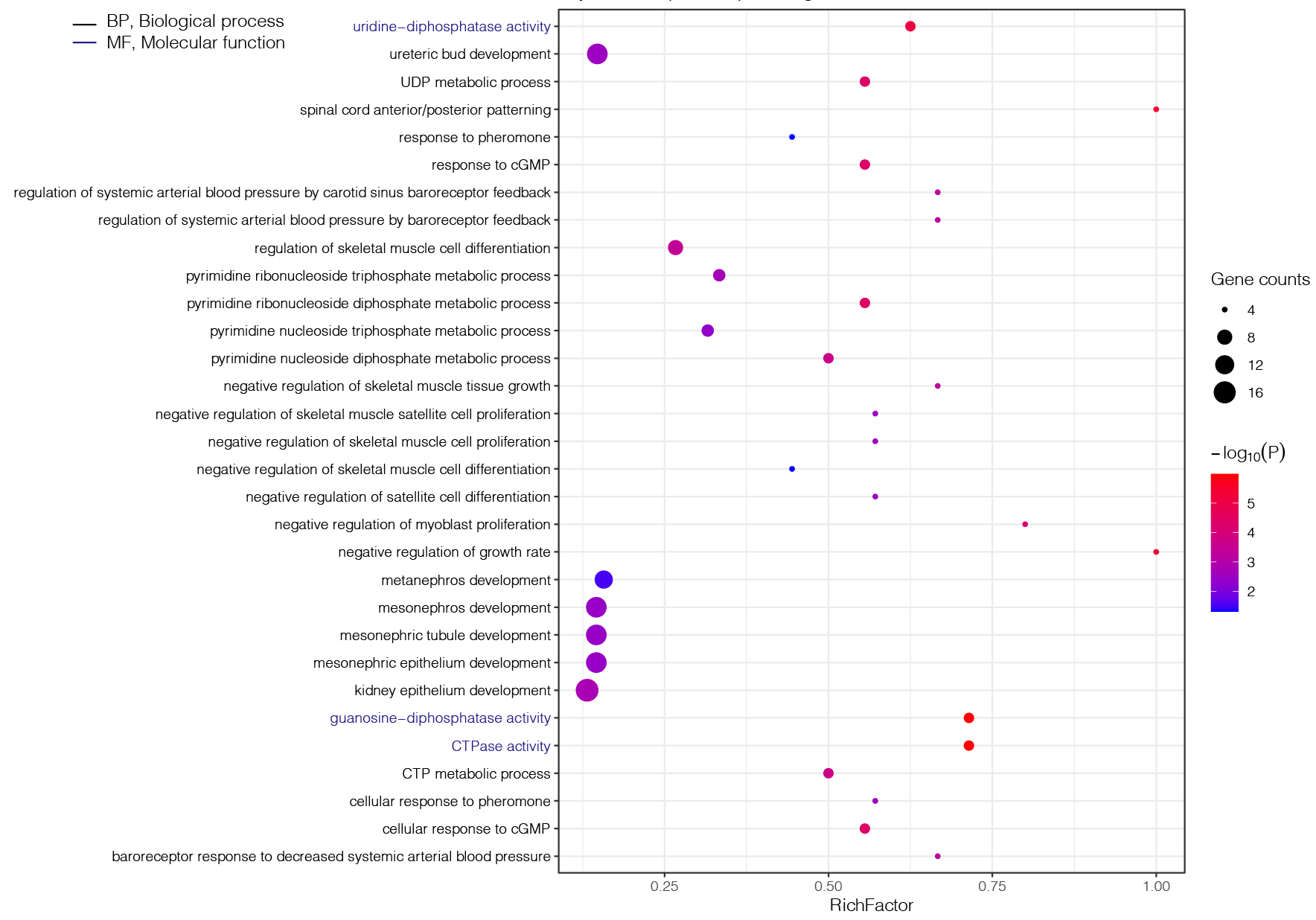


**Fig S7. Gene Ontology (GO) enrichment analysis of Stylommatophora specific genes.** Only significantly enriched terms with corrected *P* < 0.05 were indicated. The color and size of each point represented the -log_10_ (FDR) values and Gene counts. A higher -log_10_ (FDR) value and enrichment score indicated a greater degree of enrichment. Enrichment factor refers to the ratio of the number of Stylommatophora-specific genes in the pathway and the number of all annotated genes in the pathway.

**
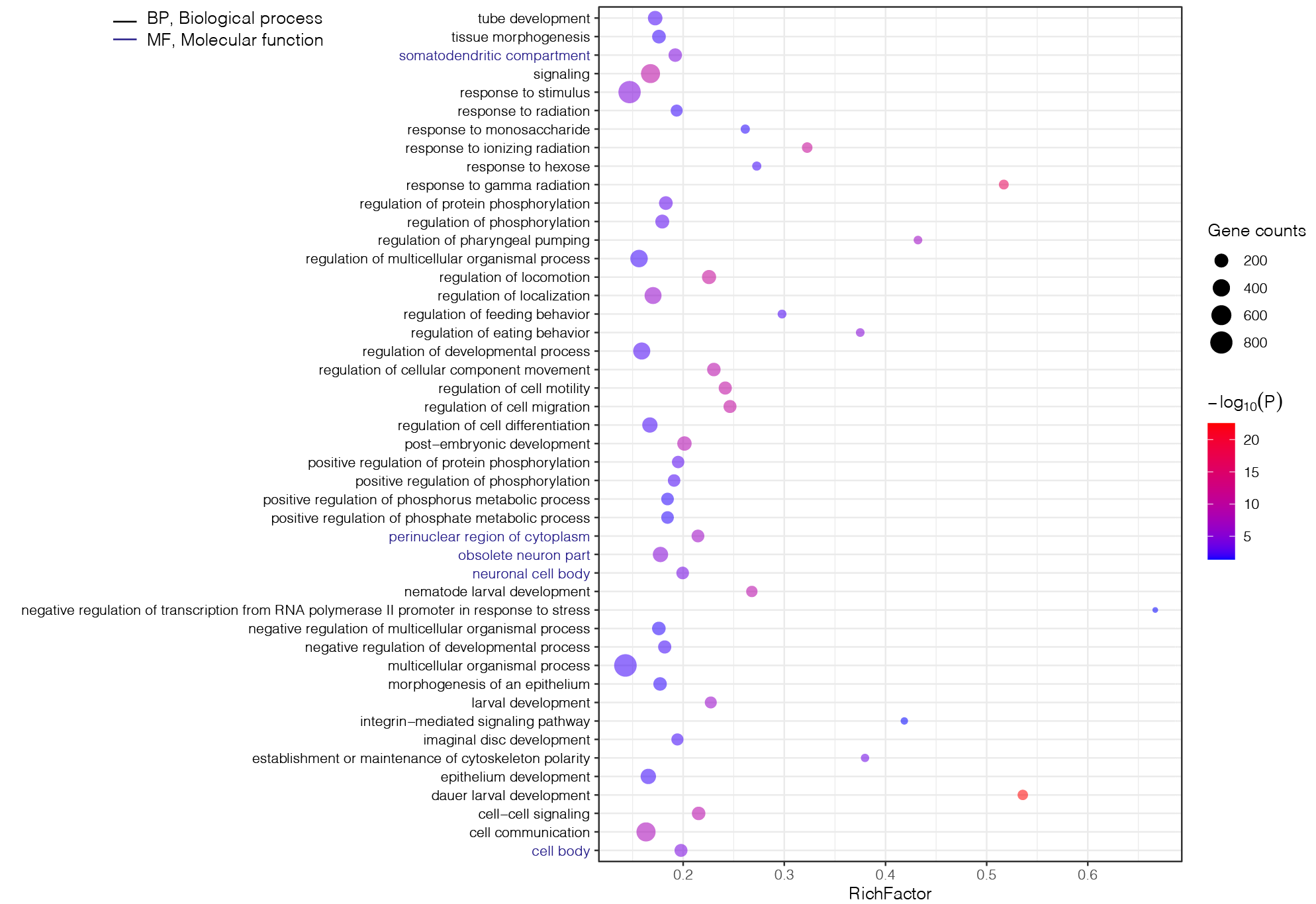
**

**Fig S8. Gene Ontology (GO) enrichment analysis of** **Stylommatophora expansion genes.** Only significantly enriched terms with corrected *P* < 0.05 were indicated. The color and size of each point represented the -log_10_ (FDR) values and gene counts. A higher -log_10_ (FDR) value and enrichment score indicated a greater degree of enrichment. Rich factor refers to the ratio of the number of Stylommatophora-expanded genes in the pathway and the number of all annotated genes in the pathway.


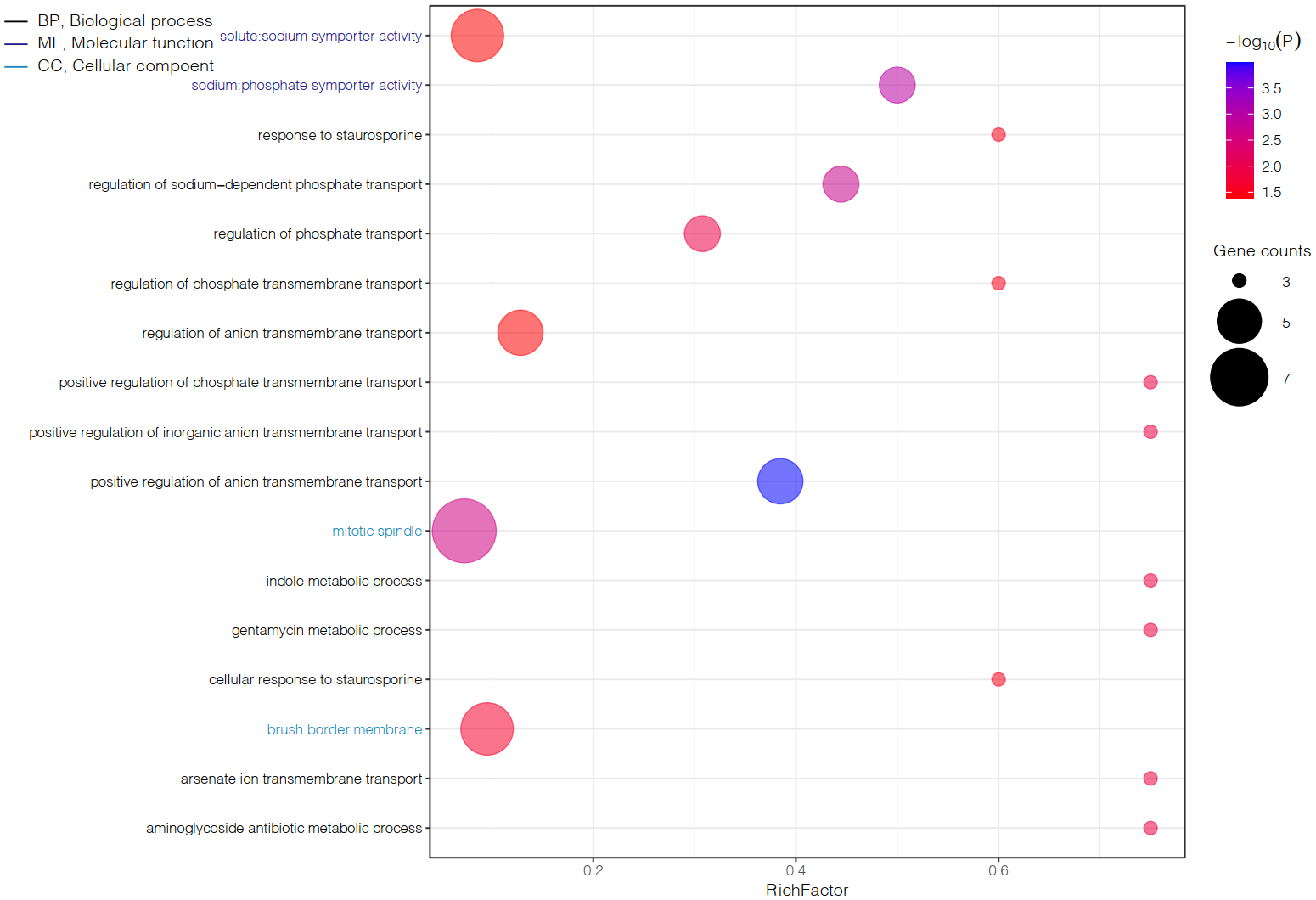


**Fig S9. Gene Ontology (GO) enrichment analysis of Stylommatophora contraction genes.** Only significantly enriched terms with corrected *P* < 0.05 were indicated. The color and size of each point represented the -log_10_ (FDR) values and Gene counts. A higher -log_10_ (FDR) value and enrichment score indicated a greater degree of enrichment. Rich factor refers to the ratio of the number of Stylommatophora-contracted genes in the pathway and the number of all annotated genes in the pathway.

**
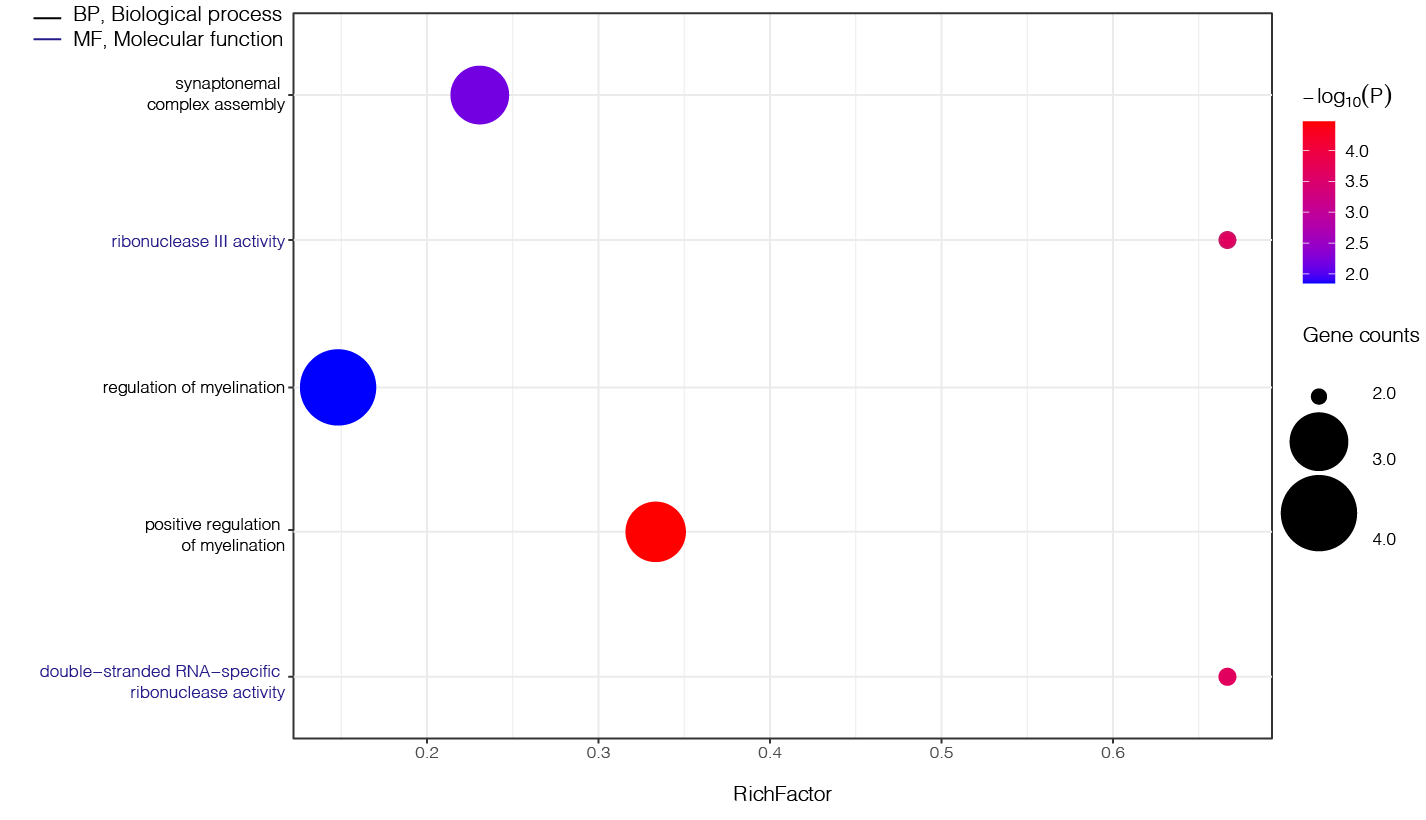
**

**Fig S10. Gene ontology (GO) Enrichment analysis of putatively positively selected genes in Stylommatophora.** Only significantly enriched terms with corrected *P* < 0.05 were indicated. The color and size of each point represented the -log_10_ (FDR) values and Gene counts. A higher -log_10_ (FDR) value and enrichment score indicated a greater degree of enrichment. Rich factor refers to the ratio of the number of Stylommatophora-positive selected genes in the pathway and the number of all genes annotated in the pathway.


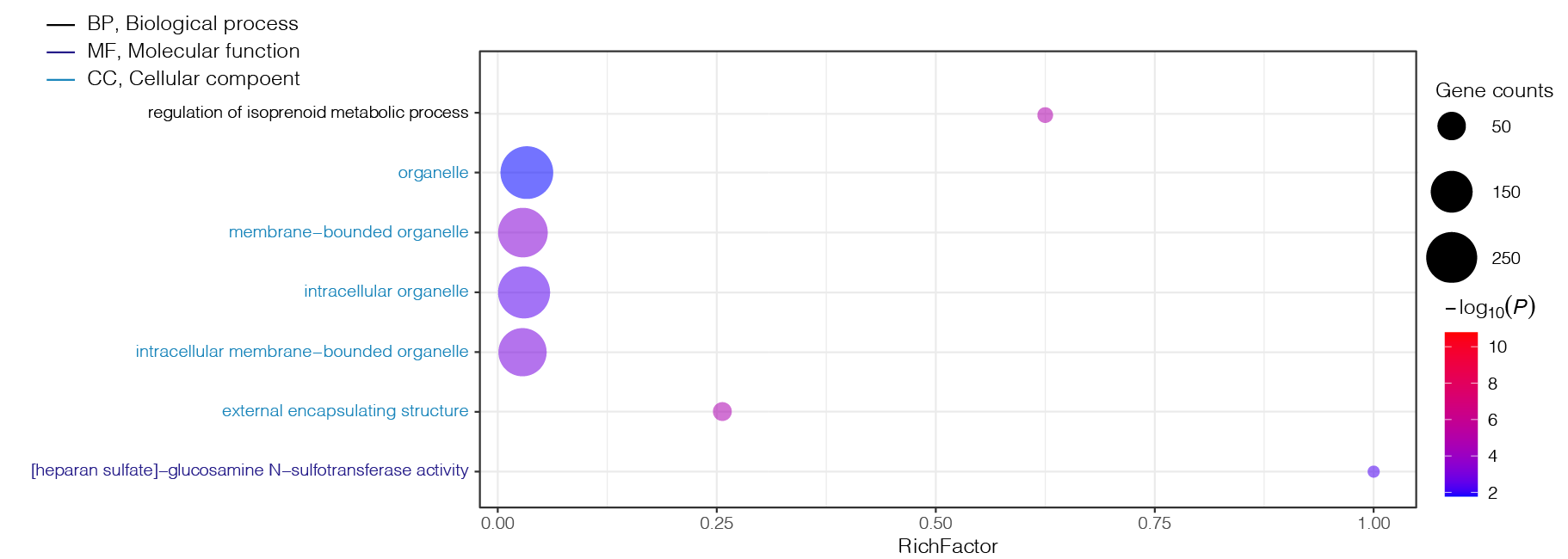


**Fig S11. Gene ontology (GO) Enrichment analysis of *Arion vulgaris* species specific genes.** Only significantly enriched terms with corrected *P* < 0.05 were indicated. The color and size of each point represented the -log_10_ (FDR) values and Gene counts. A higher -log_10_ (FDR) value and enrichment score indicated a greater degree of enrichment. Rich factor refers to the ratio of the number of *A. vulgaris* specific genes in the pathway and the number of all annotated genes in the pathway.


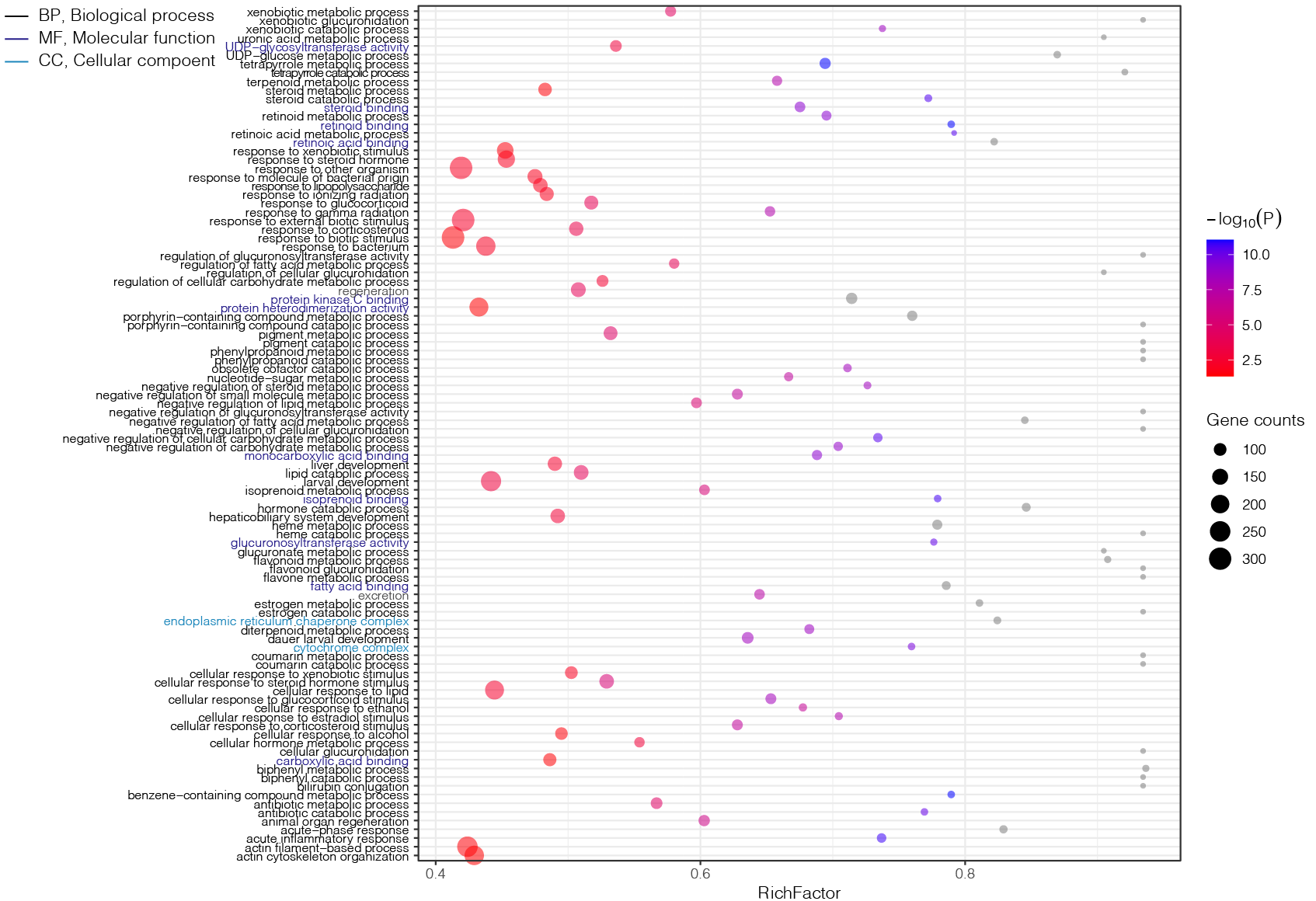


**Fig S12. Gene ontology (GO) Enrichment analysis of *Arion vulgaris* expansion genes.** Only significantly enriched terms with corrected *P* < 0.05 were indicated. The color and size of each point represented the -log_10_ (HDR) values and Gene counts. A higher -log_10_ (HDR) value and enrichment score indicated a greater degree of enrichment. Rich factor refers to the ratio of the number of *A. vulgaris* expansion genes in the pathway and the number of all annotated genes in the pathway.


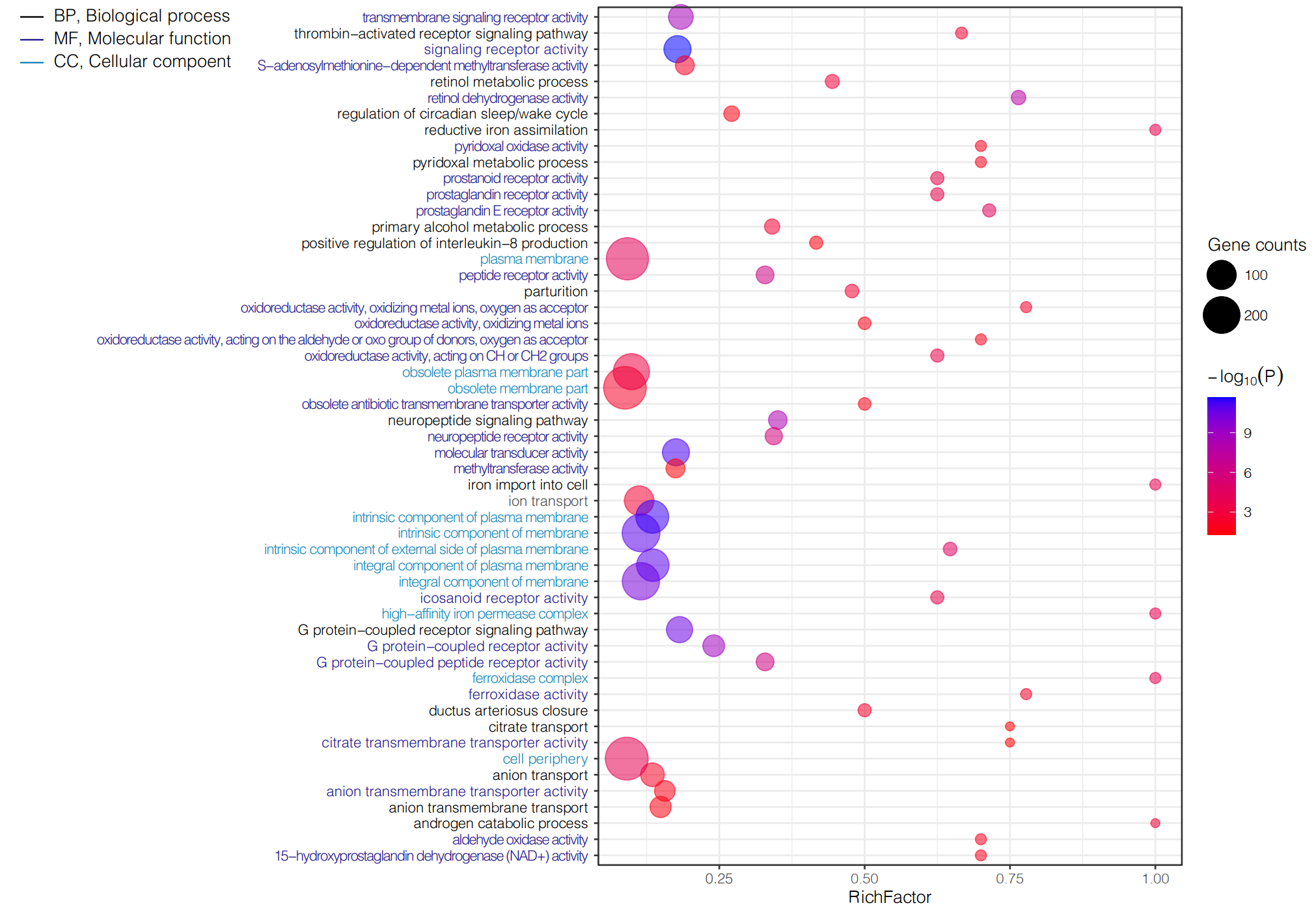


**Fig S13. Gene ontology (GO) Enrichment analysis of *Arion vulgaris* contraction genes.** Only significantly enriched terms with corrected *P* < 0.05 were indicated. The color and size of each point represented the -log_10_ (FDR) values and Gene counts. A higher -log_10_ (FDR) value and enrichment score indicated a greater degree of enrichment. Rich factor refers to the ratio of the number of *A. vulgaris* contraction genes in the pathway and the number of all annotated genes in the pathway.


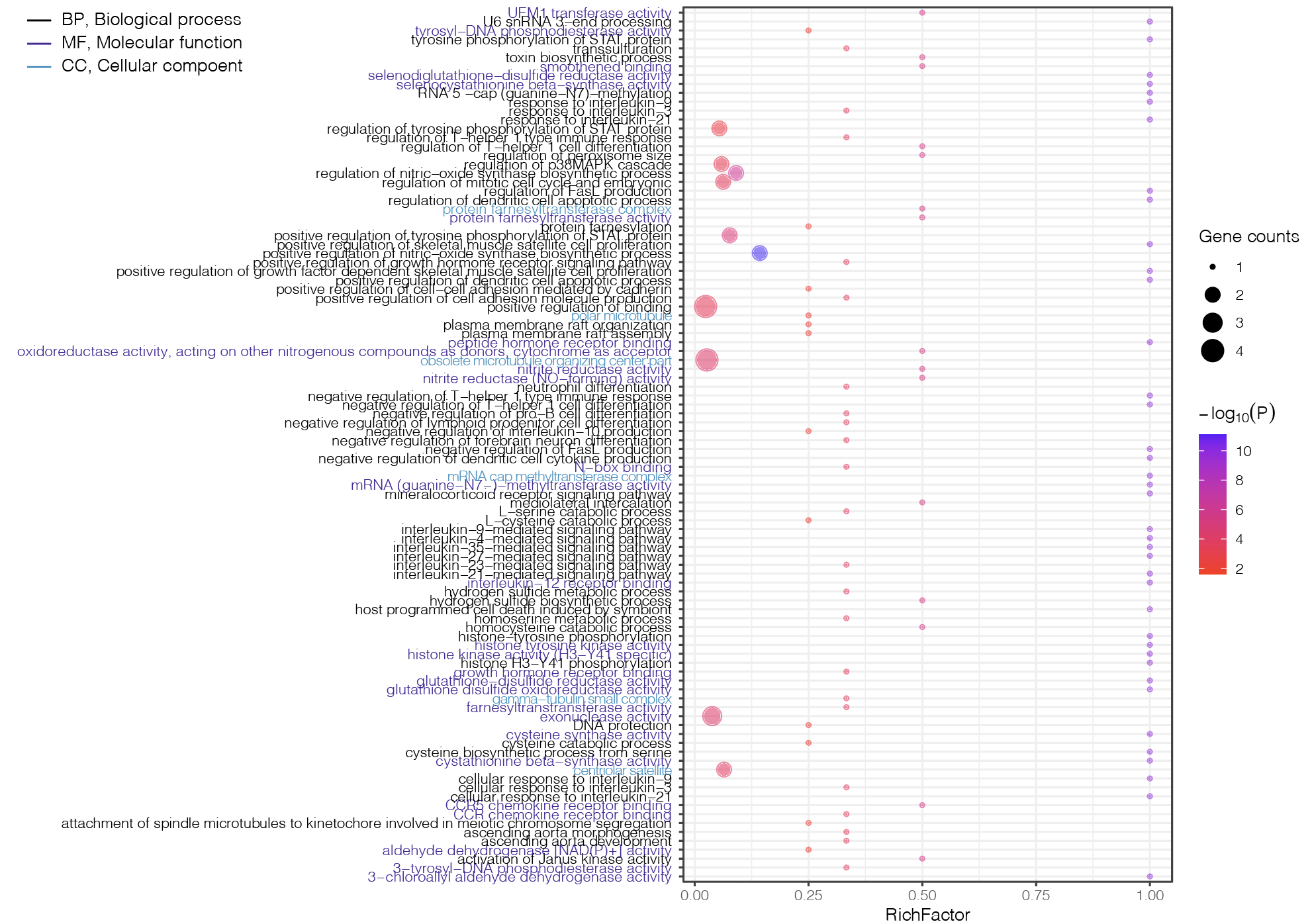


**Fig S14. Gene ontology (GO) Enrichment analysis of *Arion vulgaris* positively selected genes.** Only significantly enriched terms with corrected *P* < 0.05 were indicated. The color and size of each point represented the -log_10_ (FDR) values and Gene counts. A higher -log_10_ (FDR) value and enrichment score indicated a greater degree of enrichment. Rich factor refers to the ratio of the number of *A. vulgaris* genes in the pathway and the number of all annotated genes in the pathway.


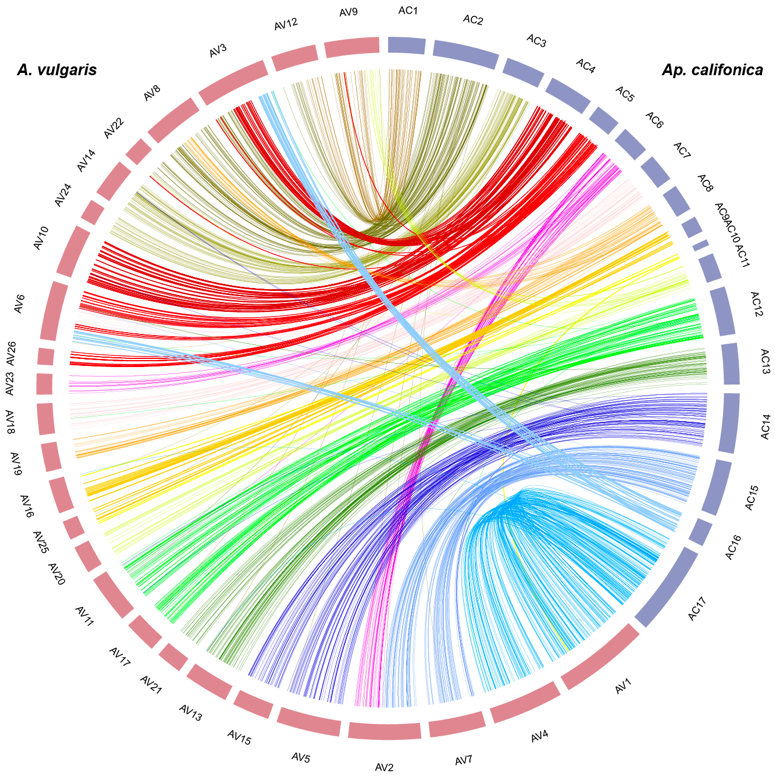


**Fig S15. A one to two corresponding relationship in the comparison of *Arion vulgaris* and *Aplysia californica* chromosomes*.***

**
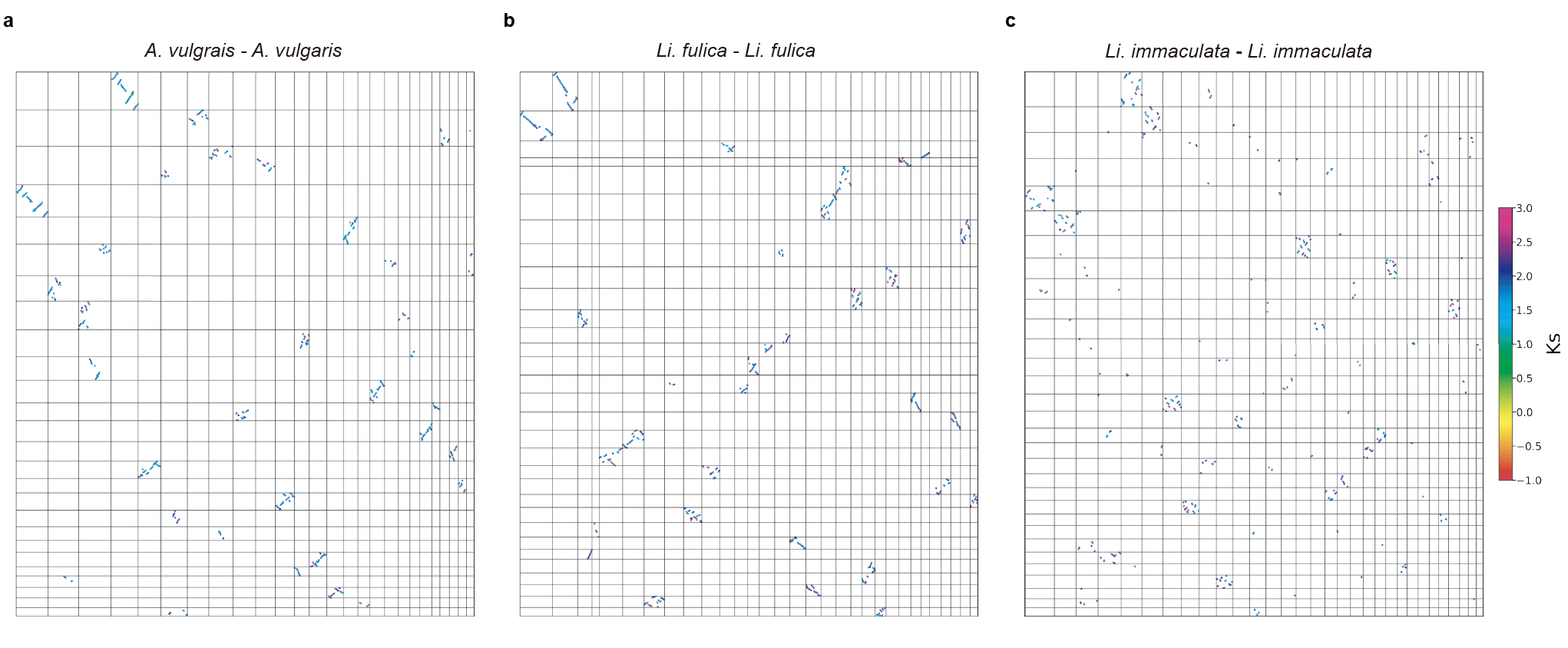
 Fig S16. *Ks* dot plot in a) *Arion vulgaris* genome, b) *Lissachatina* (*Achatina) fulica* genome, and c) *Li. immaculata* genome.** The corresponding median Ks value is shown for each syntenic block. Each block from horizontally left to right, and vertically from top to bottom in turn represents a) *A. vulgaris* chromosomes from 1-26, b) *Li. fulica* chromosomes from 1-31, and c) *Li. immaculata* chromosomes from 1-31.


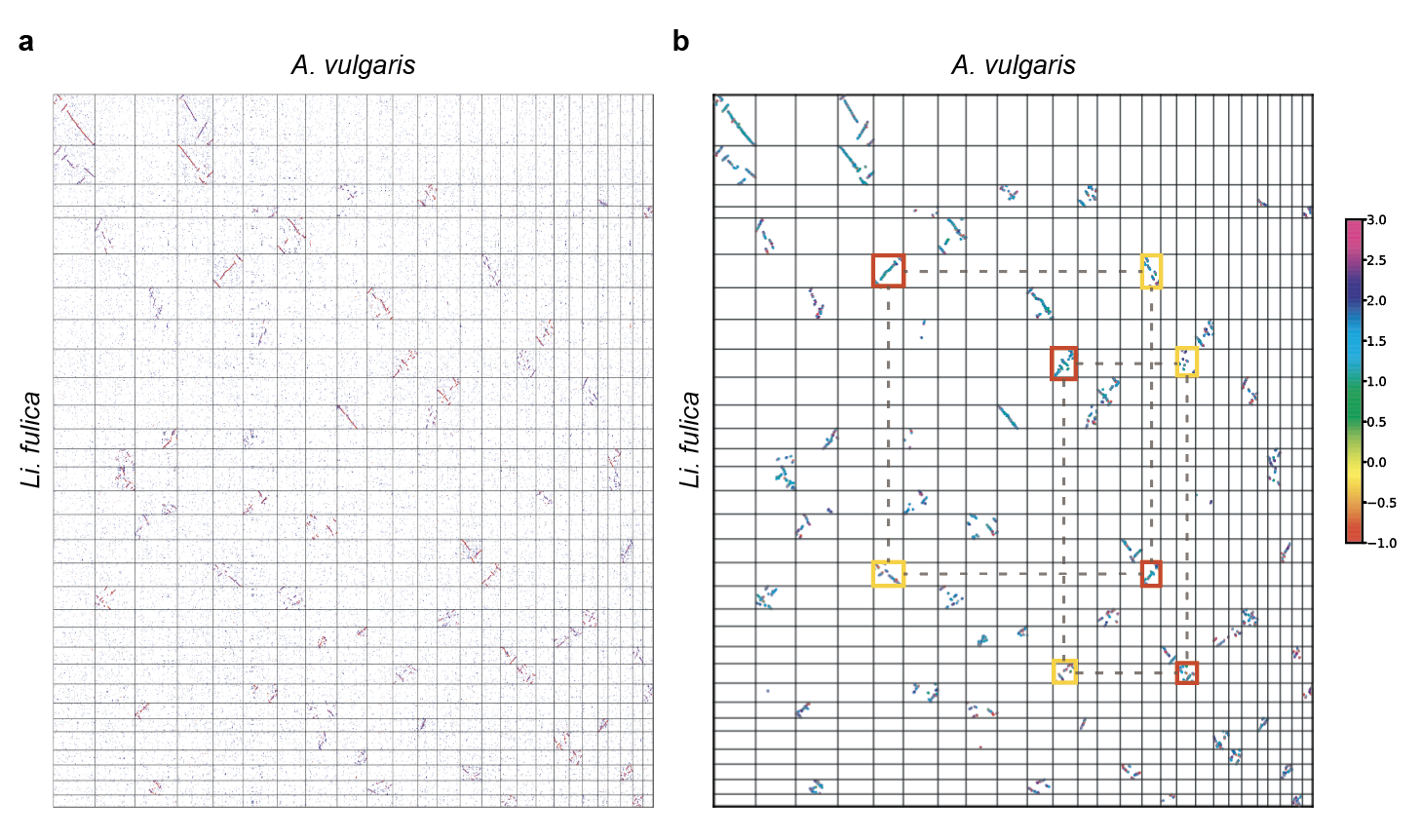


**Fig S17. (a) Homologous gene dot plot in comparison of *Arion vulgaris* and *Lissachatina* (*Achatina) fulica*.** Horizontally, from left to right, blocks, in turn represent the chromosomes 1-31 of *Li. fulica*. Vertically, from top to bottom, blocks, in turn represent chromosomes 1-26 of *A. vulgaris*. The color of red, blue, and gray dots represents the BLAST hit scores from high to low. **(b)** *Ks* dot plot of syntenic blocks in comparison of *A. vulgaris* and *Li. fulica*. Horizontally, from left to right, blocks, in turn represent the chromosomes 1-26 of *A. vulgaris*. Vertically, from top to bottom, blocks, in turn represent chromosomes 1-31 of *Li. fulica*. The chromosomes in the red box are more highly conserved than those in the yellow boxes.

*
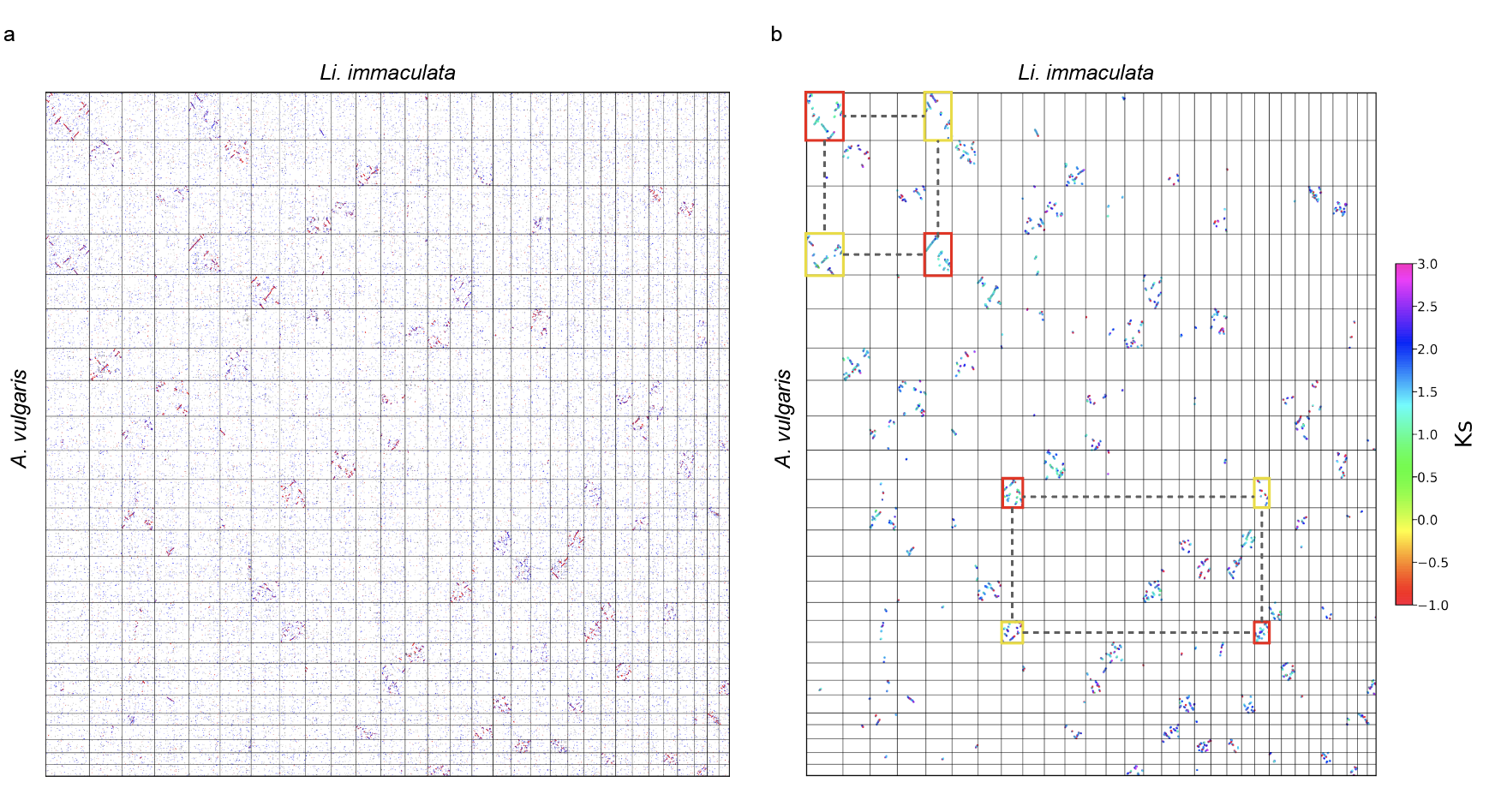
*

**Fig S18. (a) Homologous gene dot plot in comparison of *Arion vulgaris* and *Lissachatina* (*Achatina) immaculata*.** The color of red, blue, and gray dots represents the BLAST hit scores from high to low. **(b) *Ks* dot plot in comparison of *A. vulgaris* and *Li. immaculata*.** Horizontally, from left to right, blocks in turn represent the chromosomes 1-31 of *Li. immaculata*. Vertically, from top to bottom, blocks in turn represent chromosomes 1-26 of *A. vulgaris*. The chromosomes in the red box are more conservative than in the yellow box.


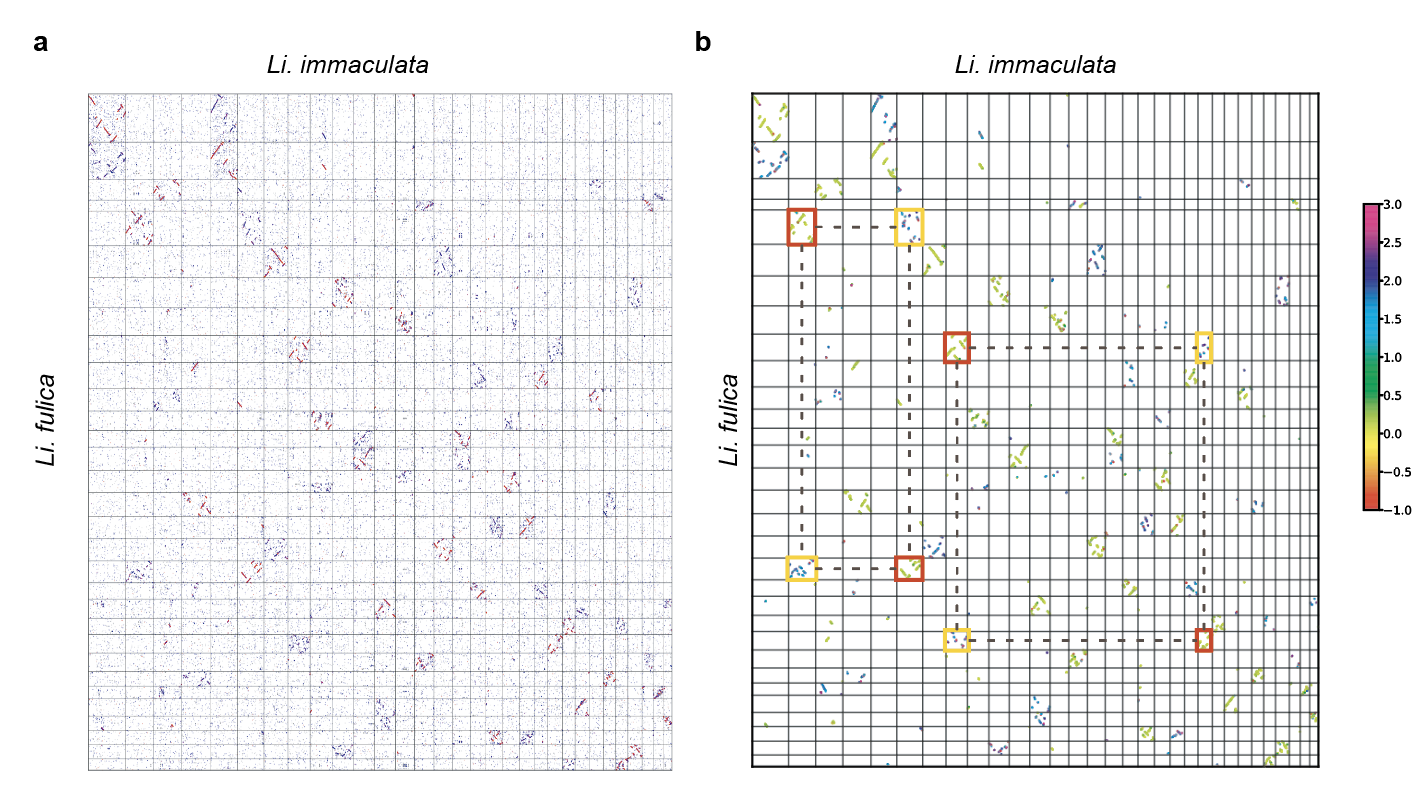


**Fig S19. (a) homologous gene dot plot in comparison of *Lissachatina* (*Achatina) fulica* and *Li. immaculata*.** The color of red, blue, and gray dots represents the BLAST hit scores from high to low. **(b) *Ks* dot plot of syntenic blocks in comparison of *Li. fulica* and *Li. immaculata*.** Horizontally, from left to right, blocks, in turn represent the chromosomes 1-31 of *Li. immaculata*. Vertically, from top to bottom, blocks, in turn represent chromosomes 1-31 of *Li. fulica*. The chromosomes in the red box are more conservative than in the yellow box.


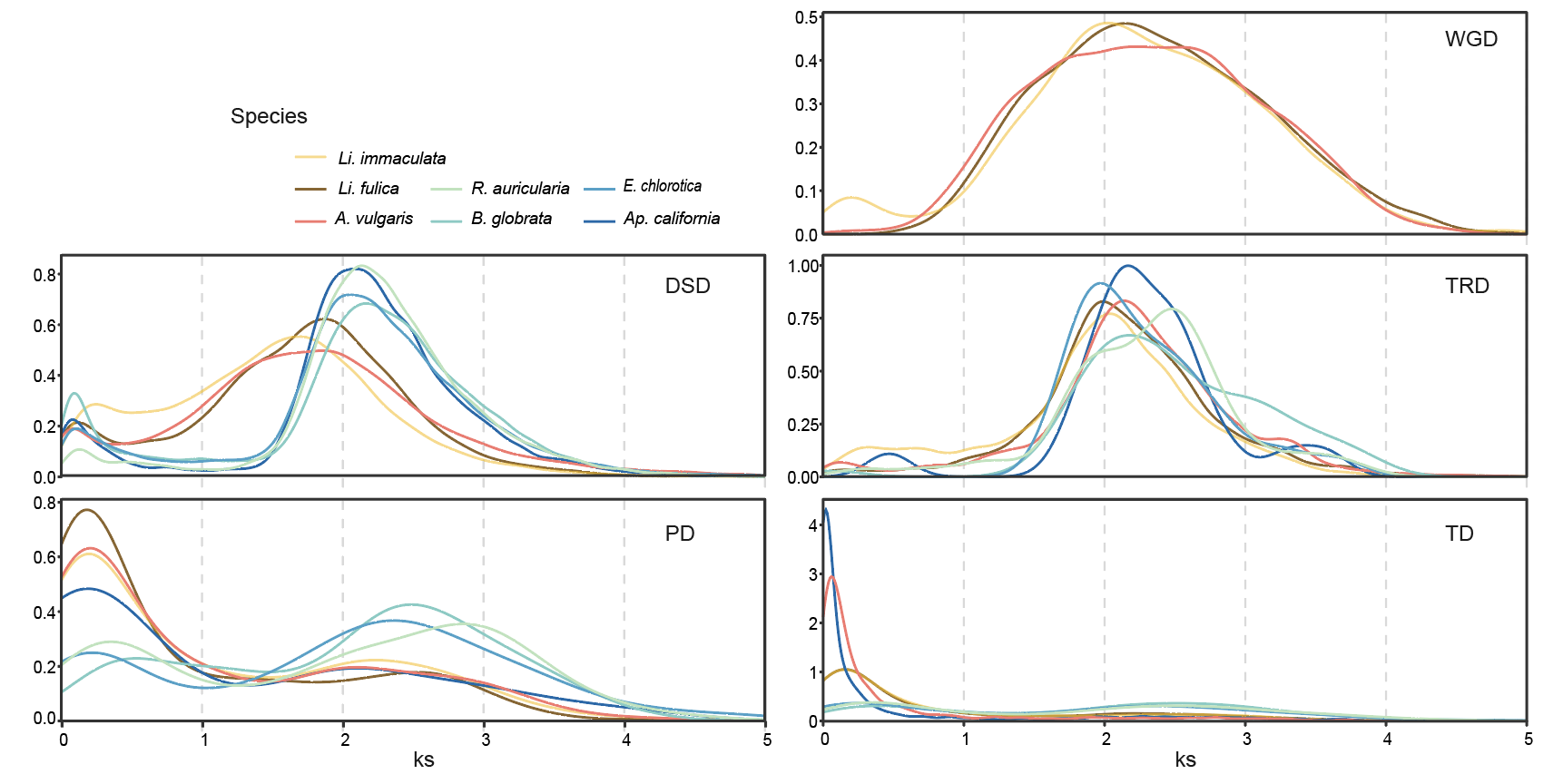


**Fig S20. *Ks* distributions of gene pairs duplicated by different modes in seven Heterobranchia species.** WGD: whole-genome duplication, DSD: dispersed duplication, TRD: transposed duplication, PD: proximal duplication, TD: tandem duplication.


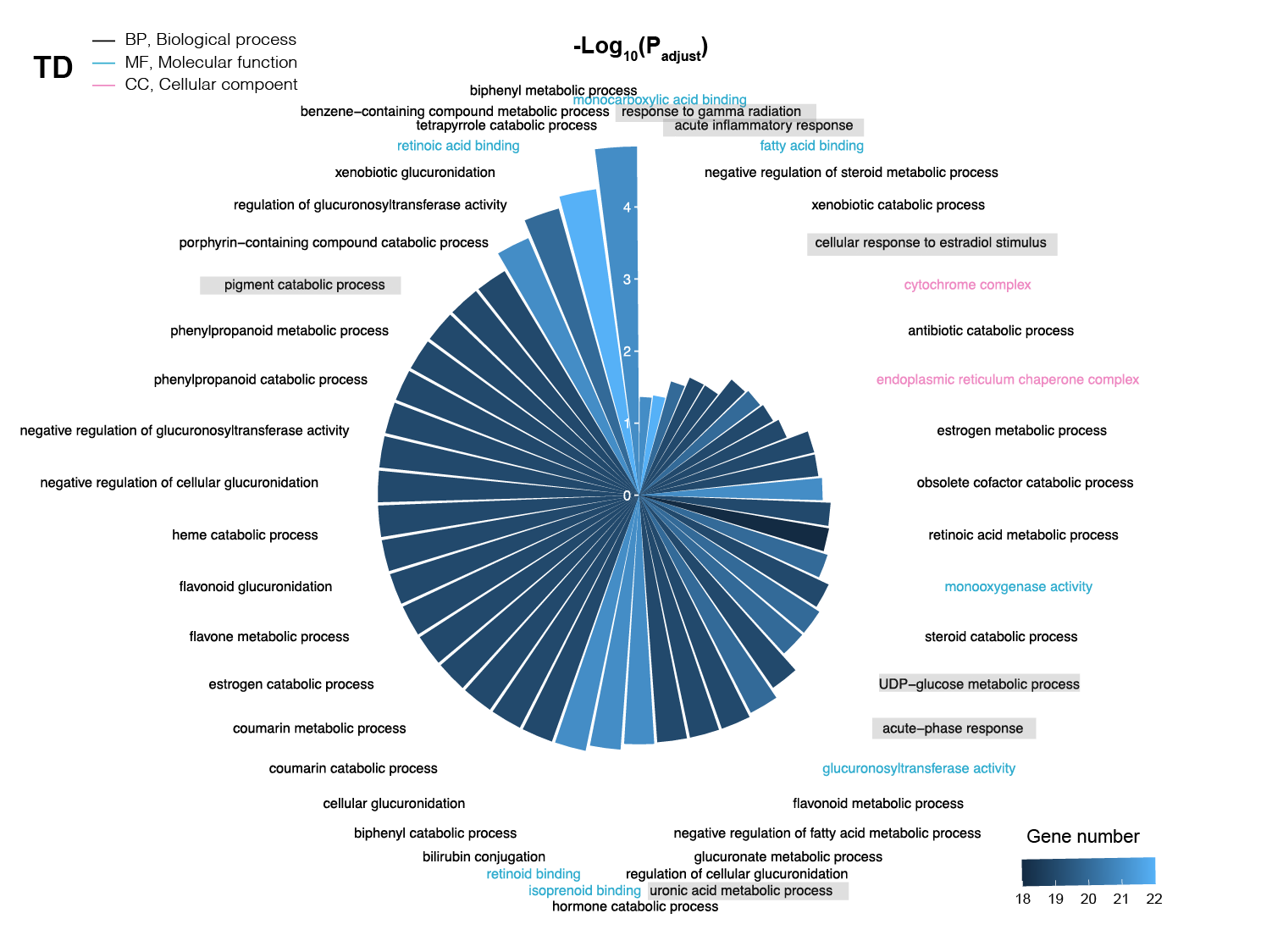


**Fig S21. Functional enrichment analysis of genes derived by tandem duplication (TD).** The enriched GO terms with corrected P value <0.005 are presented. The color represents the number of genes in a GO term. The radius represents the statistical significance of enriched GO terms. ‘P adjust’ is the Benjamini–Hochberg false discovery rate (FDR) adjusted P value. A higher -log_10_ (FDR) value and enrichment score indicated a greater degree of enrichment. Functions might be related to adaptation are marked with a gray background.

**
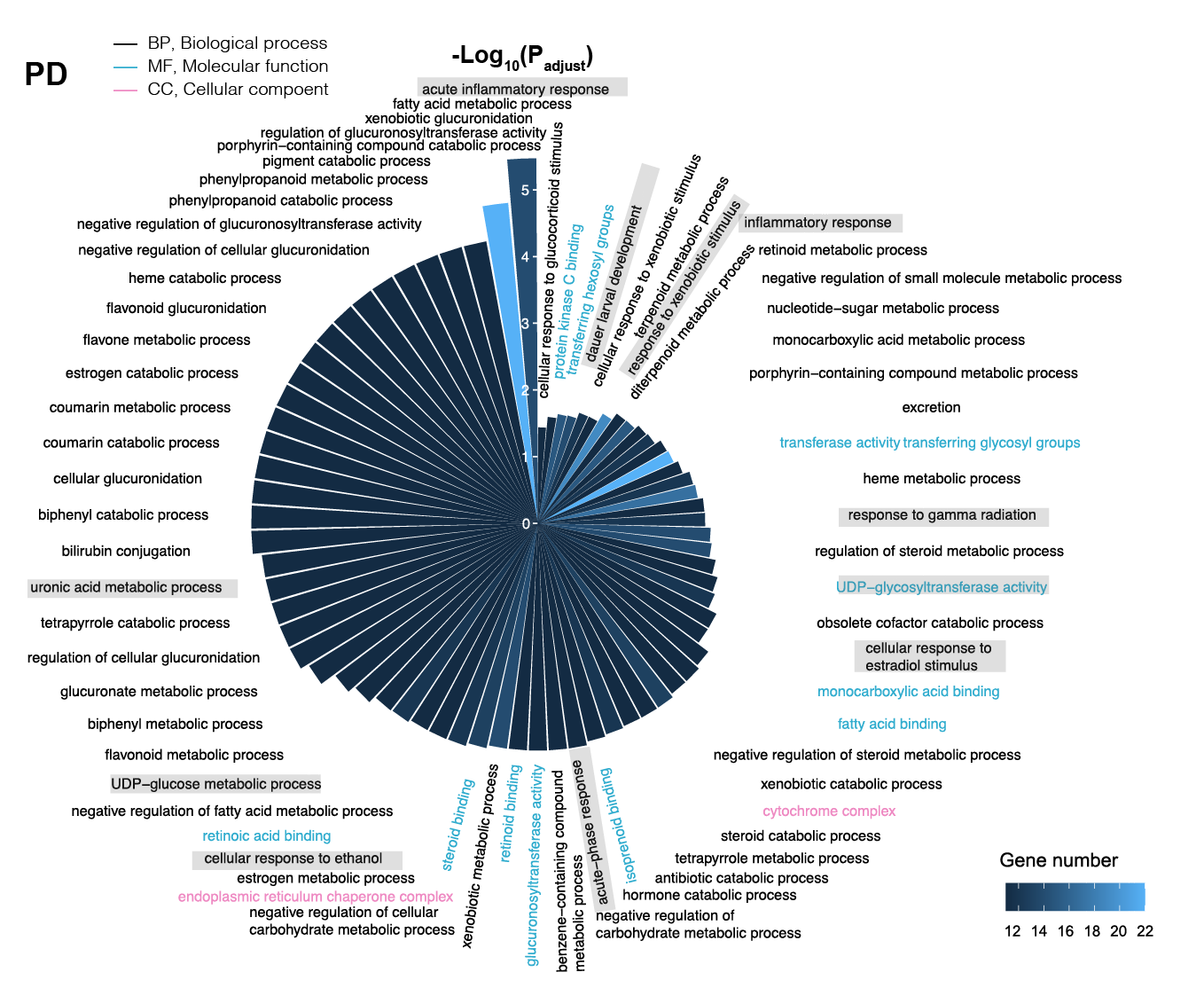
**

**Fig S22. Functional enrichment analysis of genes derived by proximal duplication (PD).** The enriched GO terms with corrected P value <0.005 are presented. The color represents the number of genes in a GO term. The radius represents the statistical significance of enriched GO terms. ‘P adjust’ is the Benjamini–Hochberg false discovery rate (FDR) adjusted P value. A higher -log_10_ (FDR) value and enrichment score indicated a greater degree of enrichment. Functions might be related to adaptation are marked with a gray background.

**
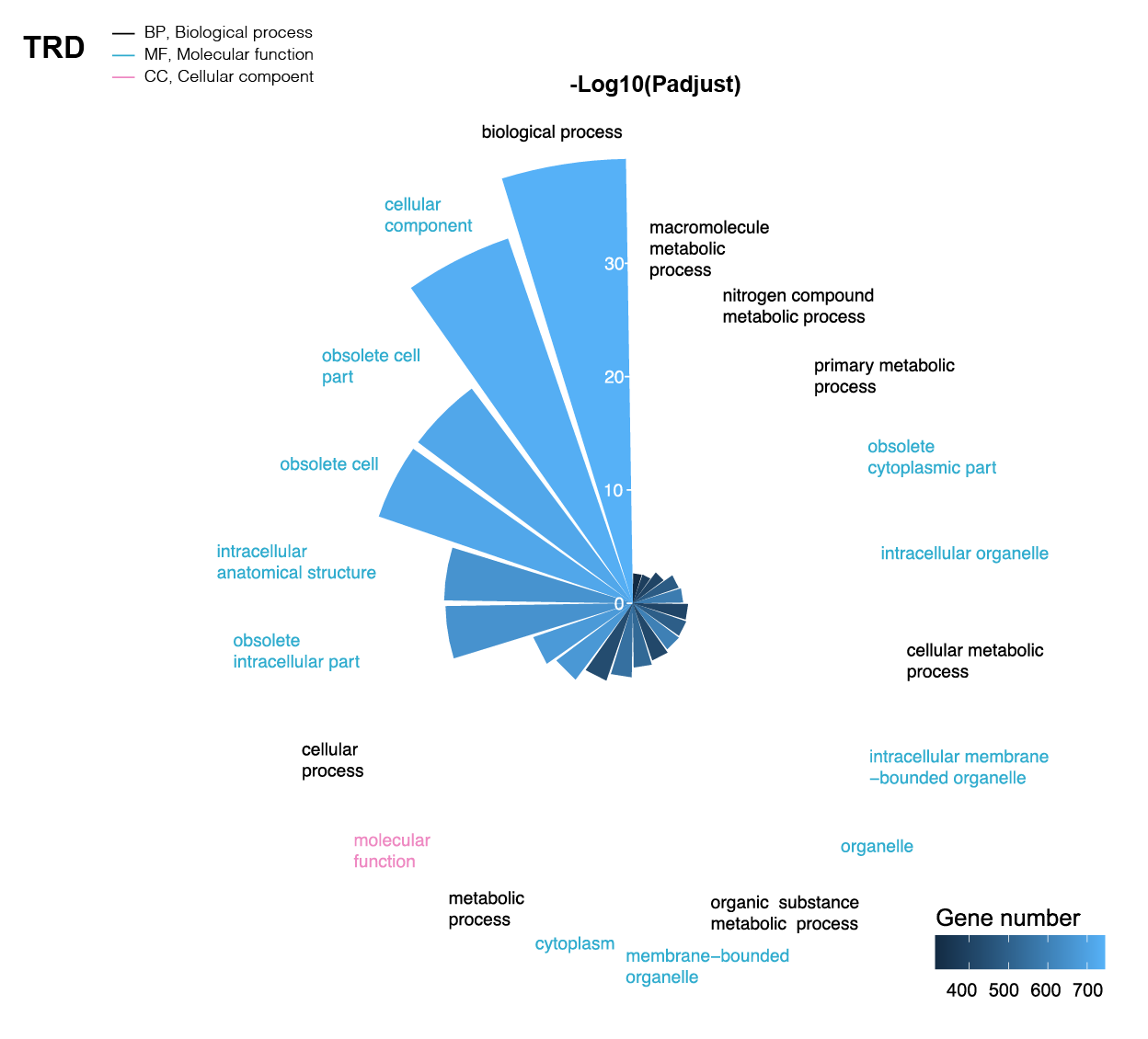
**

**Fig S23. Functional enrichment analysis of genes derived by transposed duplication (TRD).** The enriched GO terms with corrected P value <0.005 are presented. The color represents the number of genes in a GO term. The radius represents the statistical significance of enriched GO terms. ‘P adjust’ is the Benjamini–Hochberg false discovery rate (FDR) adjusted P value. A higher -log_10_ (FDR) value and enrichment score indicated a greater degree of enrichment. Functions might be related to adaptation are marked with a gray background.


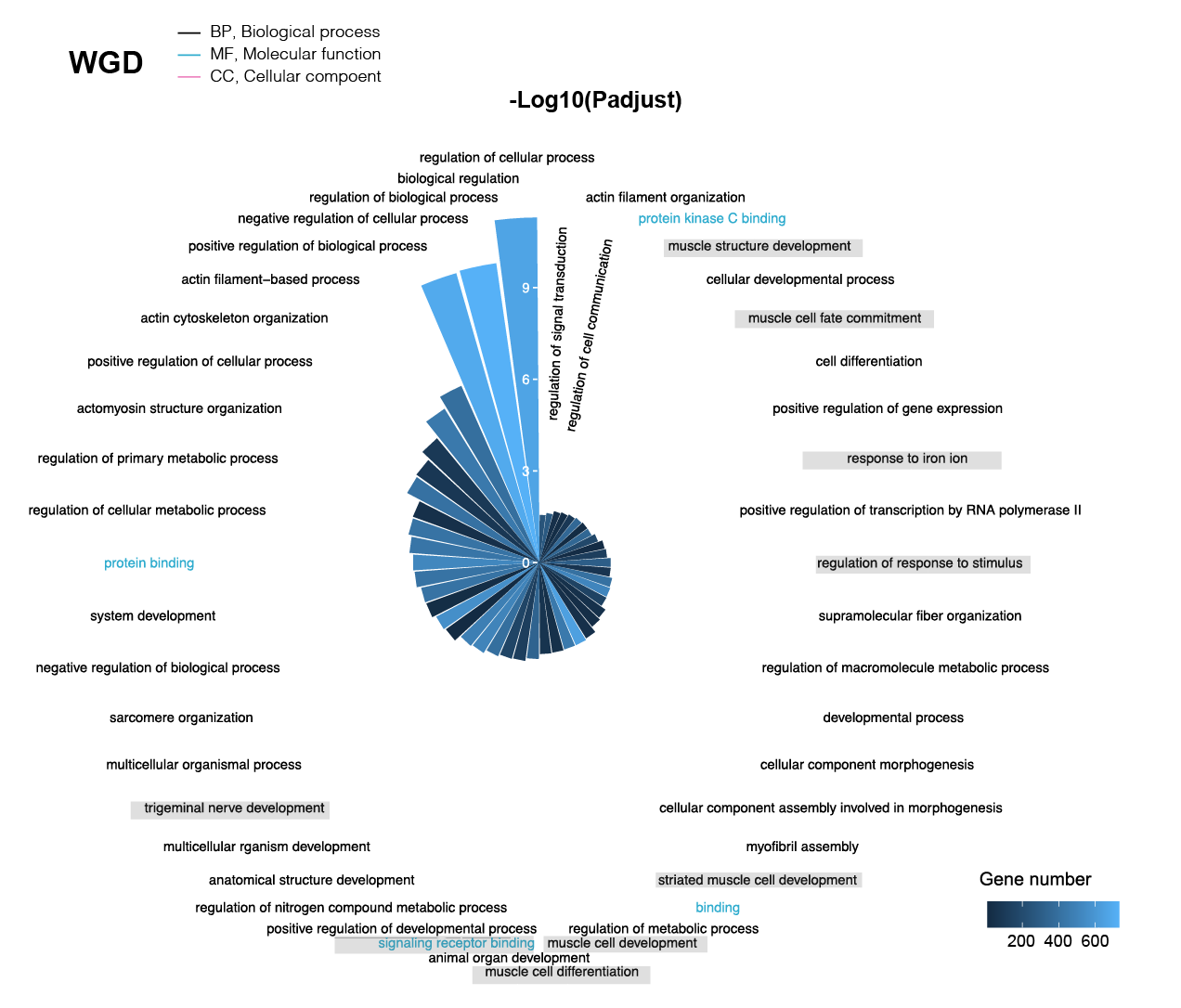


**Fig S24. Functional enrichment analysis of genes derived by whole genome duplication (WGD).** The enriched GO terms with corrected P value <0.005 are presented. The color represents the number of genes in a GO term. The radius represents the statistical significance of enriched GO terms. ‘P adjust’ is the Benjamini–Hochberg false discovery rate (FDR) adjusted P value. A higher -log_10_ (FDR) value and enrichment score indicated a greater degree of enrichment. Functions might be related to adaptation are marked with a gray background.


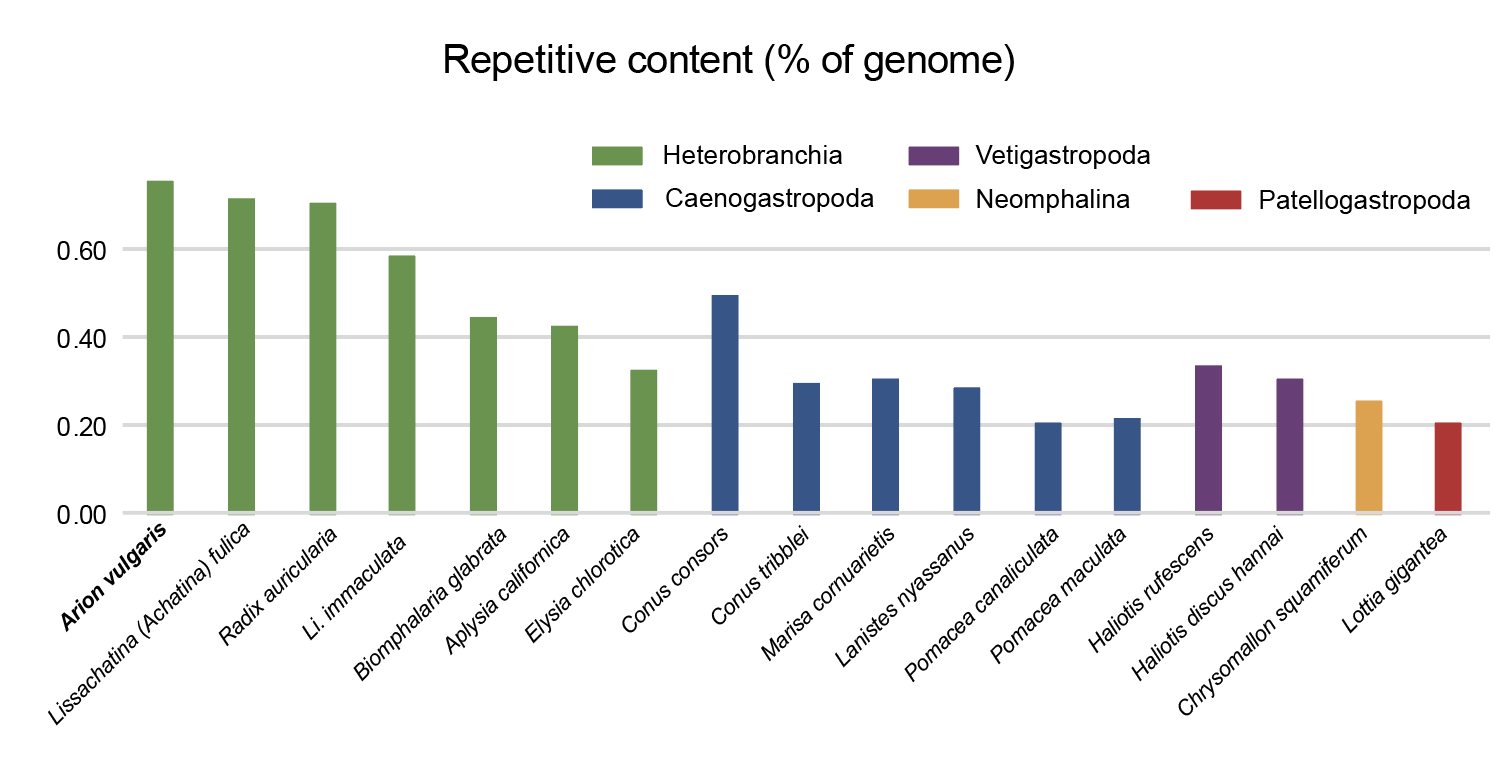


**Fig S25. Repetitive content in published gastropod genomes.**

**
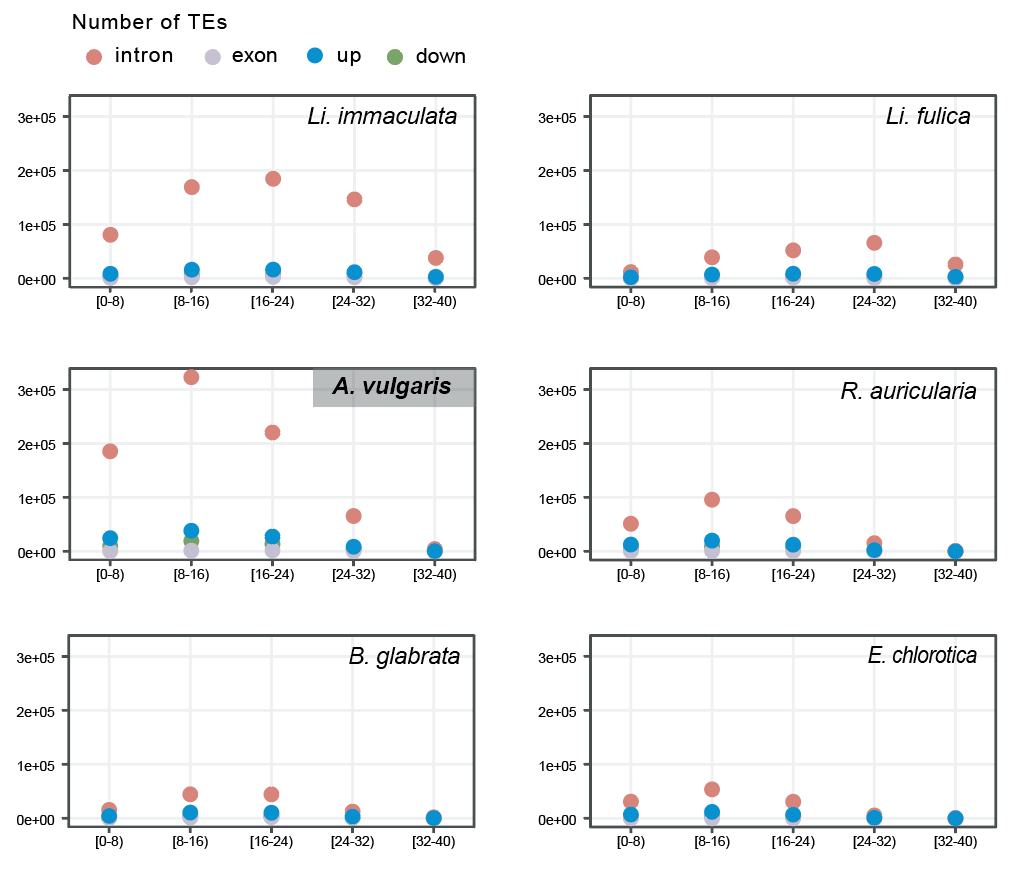
**

**Fig S26. Dot plot shows the number of TEs in different genic regions under different ages.** Percentage of divergence from consensus is used as a proxy for age: the older the invasion of the TEs, the more copies will have accumulated mutations. Note, we did not include *Ap. californica* in this analysis, as the gene annotation file and TEs annotation file of *Ap. californica* is inconsistent.

**
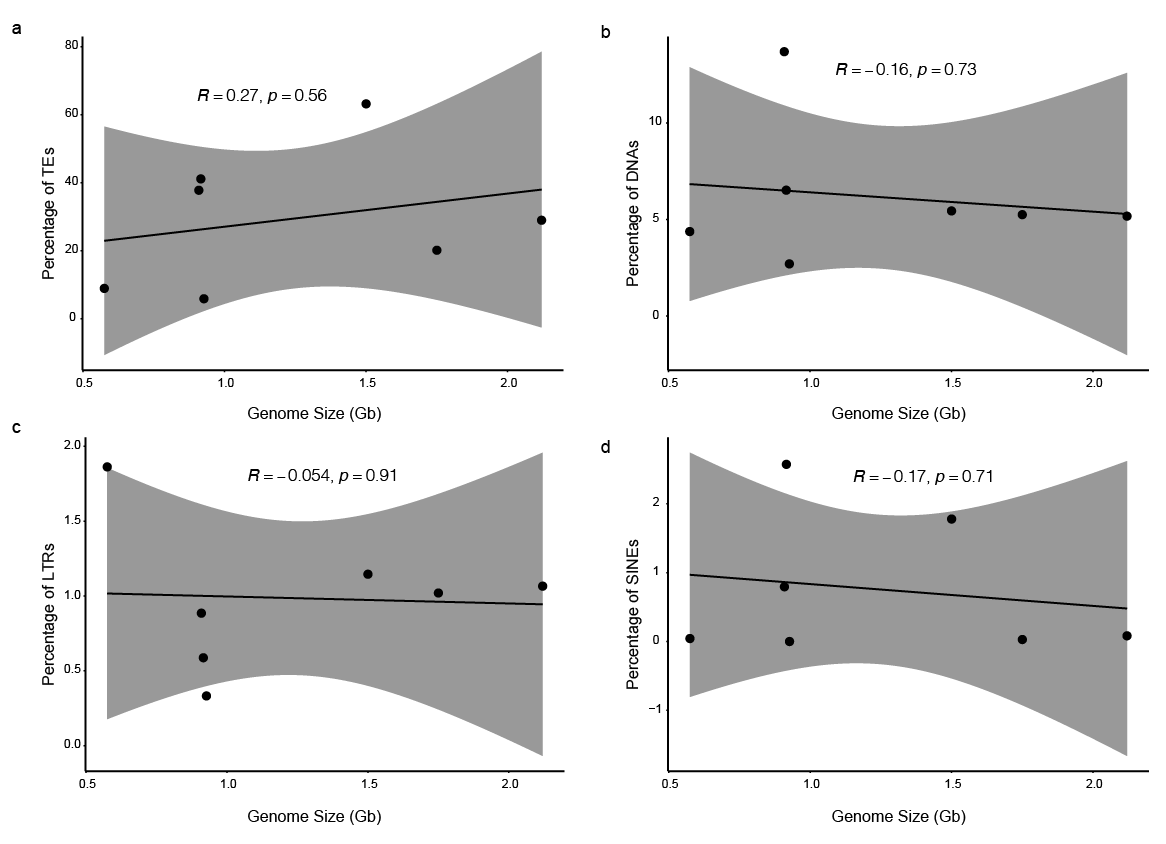
Fig S27. Relationship between total TE coverage (a), DNAs coverage (b), LTRs coverage (c), SINEs coverage (d), and genome size in Heterobranchia species.** TE: transposable element; DNAs: DNA transposons; LTR: Long Terminal Repeat; SINEs: Short Interspersed Nuclear Elements.

**
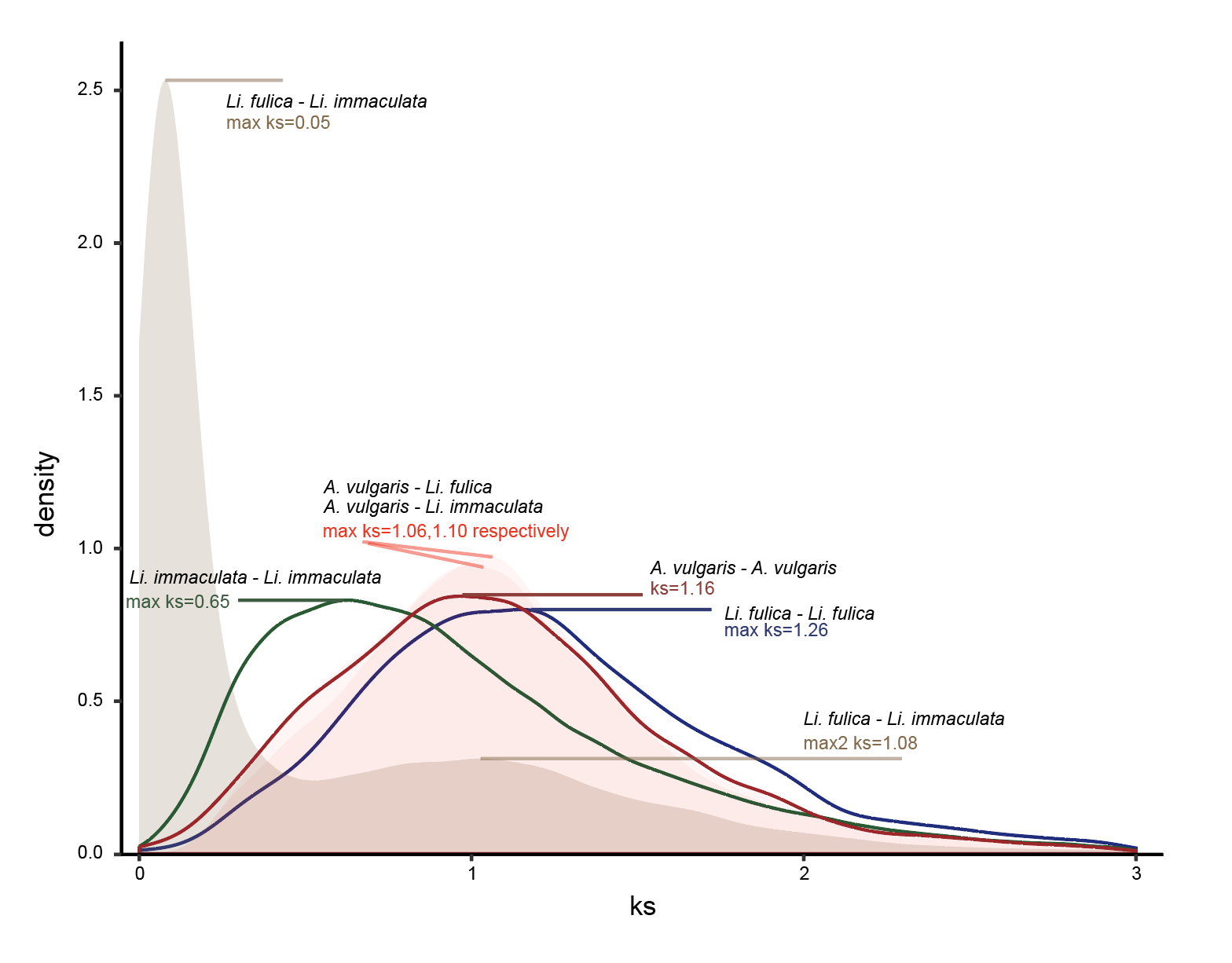
**

**Fig S28. Frequency distributions of synonymous substitutions (*Ks*) for homologous gene pairs identified using MCScanX in comparisons of *Arion vulgaris*, *Lissachatina* (*Achatina) fulica* and *Li. immaculata*.** Shaded areas represent speciation events and solid lines represent whole-genome duplication events.

**Table S1. Sequencing data generated for *Arion vulgaris* genome assembly and annotation.**

| Library type | Platform | Total bases (Gb) | Application |
| --- | --- | --- | --- |
| Short reads | HiSeq X Ten | 56.83 | Genome survey and genomic base correction |
| Linked reads | HiSeq X Ten | 138.21 | Genome survey, genomic base correction and genome assembly |
| Nanopore | Nanopore PromethION | 74.94 | Genome assembly |
| HiC | HiSeq X Ten | 135.28 | Chromosome construction |
| mRNA-Seq | NovaSeq 6000 | 6.75 | Genome annotation |

**Table S2.** **Statistics of assembly at different assemble stages.**

| Characteristic | wtdbg2 | wtdbg2-ntEdit-Scaff10X | instaGRAAL |
| --- | --- | --- | --- |
|  | Nanopore reads | Nanopore reads, short reads, linked reads | Nanopore reads, short reads, linked reads, HiC reads |
| Total Contig length (Gb) | 1.535 (6,109) | 1.543 (6,109) | 1.541 (7,076) |
| Contig N50 (Mb) | 4.466 (93) | 4.488 (93) | 8.603 (49) |
| Longest Contig (Mb) | 23.976 | 24.090 | 39.732 |
| Total scaffold length (Gb) | - | 1.543 (5,786) | 1.543 (6,751) |
| Scaffold N50(Mb) | - | 7.660 (55) | 64.342 (10) |
| Longest Scaffold (Mb) | - | 34.170 | 114.239 |
| N's per 100 kbp (bp) | - | 2.09 | 2.09 |
| Complete BUSCOs | 0.84 | 0.90 | 0.91 |
| Complete and single-copy BUSCOs | 0.82 | 0.84 | 0.85 |
| Complete and duplicated BUSCOs | 0.02 | 0.06 | 0.06 |
| Fragmented BUSCOs (F) | 0.03 | 0.02 | 0.02 |
| Missing BUSCOs (M) | 0.13 | 0.08 | 0.07 |

Note: The number in brackets indicates the number of corresponding scaffolds and contigs.

**Table S3: Species used for comparative genomics analysis and data citations in this study.**

| Clade | Species | GenBank/Database accession |
| --- | --- | --- |
| H | *Arion vulgaris* | GCA_020796225.1 |
| H | *Lissachatina* (*Achatina) immaculata*^1^ | GCA_009760885.1 |
| H | *Li. fulica*^2^ | gigadb.org/dataset/100647 |
| H | *Aplysia californica^3^* | GCA_000002075.2 (For gene family, phylogeny, repeats analysis) |
|  |  | https://www.dnazoo.org/assemblies/Aplysia_californica (For macrosynteny analysis) |
| H | *Biomphalaria glabrata^4^* | GCA_000457365.1 |
| H | *Elysia chlorotica^5^* | GCA_003991915.1 |
| H | *Radix auricularia^6^* | GCA_002072015.1 |
| C | *Pomacea canaliculata^7^* | GCA_004794335.1 |
| C | *Marisa cornuarietis^7^* | GCA_004794655.1 |
| C | *Conus consors^8^* | GCA_004193615.1 |
| C | *Lanistes nyassanus^7^* | GCA_004794575.1 |
| V | *Haliotis rufescens^9^* | GCA_003343065.1 |
| N | *Chrysomallon squamiferum^10^* | GCA_012295275.1 |
| P | *Lottia gigantea^11^* | GCA_000327385.1 |
| B | *Argopecten purpuratus^12^* | gigadb.org/dataset/100419 |
| B | *Saccostrea glomerata^13^* | GCA_003671525.1 |

**Note:** Clade H, C, V, N, P represents Heterobranchia, Caenogastropoda, Vetigastropoda, Neomphalina, and Patellogastropoda respectively. B: Bivalvia

**Table S4: Evidence weight file used for *Arion vulgaris* gene prediction.**

| Item | | weight |
| --- | --- | --- |
| Protein | |  |
|  | *Lissachatina* (*Achatina) fulica* | 4 |
|  | *Biomphalaria glabrata* | 2 |
|  | *Elysia chlorotica* | 2 |
|  | *Haliotis rufescens* | 2 |
|  | *Pomacea canaliculata* | 2 |
|  | *Aplysia californica* | 2 |
| *ab initio* prediction | | 1 |
| Transcript | | 6 |

Table S5: Statistics of predicted protein-coding genes in the *Arion vulgaris* genome.

| Gene set | | Number | Average transcript  length(bp) | Average CDs  length(bp) | Average exon  per gene | Average exon  length(bp) | Average intron  length(bp) |
| --- | --- | --- | --- | --- | --- | --- | --- |
| homolog prediction | |  |  |  |  |  |  |
|  | *Lissachatina* (*Achatina) fulica* | 22,718 | 10,568.96 | 1,202.44 | 4.60 | 261.53 | 2,603.49 |
|  | *Aplysia californica* | 12,046 | 15,833.02 | 1,412.34 | 7.39 | 191.22 | 2,258.25 |
|  | *Biomphalaria glabrata* | 16,264 | 15,628.49 | 1,325.09 | 7.06 | 187.81 | 2,362.09 |
|  | *Elysia chlorotica* | 13,069 | 13,604.99 | 1,204.99 | 6.42 | 187.82 | 2,289.63 |
|  | *Haliotis rufescens* | 21,393 | 5,791.92 | 863.26 | 2.82 | 305.68 | 2,701.96 |
|  | *Pomacea canaliculata* | 10,993 | 21,930.11 | 1,520.19 | 9.41 | 161.60 | 2,427.71 |
| *De novo* prediction | | 44,246 | 12,668.01 | 1,146.02 | 5.08 | 225.44 | 2,821.68 |
| transcriptome prediction | | 161,478 | 62,36.37 | 910.84 | 2.14 | 424.68 | 4,652.03 |
| final gene set | | 32,518 | 15,429.25 | 1,291.68 | 5.70 | 226.43 | 3,005.06 |

**Table S6: Functional annotation of the predicted genes models.**

|  | Number | Percent (%) |
| --- | --- | --- |
| eggNOG-mapper | 16,791 | 0.51636017 |
| KEGG | 9,642 | 0.296512701 |
| InterPro | 26,520 | 0.815548312 |
| SwissProt | 31,728 | 0.975705763 |
| TrEMBL | 31,429 | 0.966510856 |
| Total | **31,763** | 0.97678209 |

**Table S7:** Summary of gene family clustering of 16 mollusk species.

| Species | total genes | genes in family | unassigned genes | family number | unique family | genes in unique family | genes  per family |
| --- | --- | --- | --- | --- | --- | --- | --- |
| *Arion vulgaris* | 32,518 | 30,636 | 1,882 | 13,333 | 253 | 881 | 2.30 |
| *Lissachatina (Achatina) fulica* | 23,726 | 23,073 | 653 | 12,390 | 79 | 310 | 1.86 |
| *Li. immaculata* | 30,194 | 28,633 | 1,561 | 12,513 | 124 | 361 | 2.29 |
| *Biomphalaria glabrata* | 25,550 | 24,093 | 1,457 | 12,444 | 366 | 1,450 | 1.94 |
| *Radix auricularia* | 17,338 | 16,237 | 1,101 | 10,310 | 110 | 367 | 1.57 |
| *Aplysia californica* | 27,576 | 26,761 | 815 | 12,191 | 396 | 1,601 | 2.20 |
| *Elysia chlorotica* | 24,980 | 21,936 | 3,044 | 12,980 | 312 | 1,255 | 1.69 |
| *Pomacea canaliculata* | 18,273 | 17,728 | 545 | 12,042 | 47 | 159 | 1.47 |
| *Marisa cornuarietis* | 23,827 | 21,988 | 1,839 | 13,181 | 201 | 1,005 | 1.67 |
| *Lanistes nyassanus* | 20,938 | 19,651 | 1,287 | 12,475 | 138 | 555 | 1.58 |
| *Conus consors* | 17,715 | 17,023 | 692 | 7,654 | 224 | 611 | 2.22 |
| *Chrysomallon squamiferum* | 16,917 | 15,498 | 1,419 | 9,904 | 252 | 1,047 | 1.56 |
| *Lottia gigantea* | 23,340 | 21,940 | 1,400 | 12,165 | 408 | 2,794 | 1.80 |
| *Haliotis rufescens* | 48,956 | 44,060 | 4,896 | 14,580 | 1,622 | 8,303 | 3.02 |
| *Saccostrea glomerata* | 26,956 | 26,327 | 629 | 11,516 | 619 | 3,375 | 2.29 |
| *Argopecten purpuratus* | 24,705 | 22,797 | 1,908 | 12,568 | 501 | 2,069 | 1.81 |

**Table S8: GO enrichment analysis of Stylommatophora specific gene families.** For each GO subcategory, a 2 × 2 contingency table was constructed by recording the numbers of genes included or not included in a category of ‘genome background’ genes and Stylommatophora-specific genes. Two-tailed Fisher’s exact test was used to calculate statistical significance. BP: biology process; MF: molecular function.

| GO | Type | Function | P value | Gene number |
| --- | --- | --- | --- | --- |
| GO:0072073 | BP | kidney epithelium development | 0.001666 | 17 |
| GO:0001657 | BP | ureteric bud development | 0.002973 | 14 |
| GO:0001823 | BP | mesonephros development | 0.003725 | 14 |
| GO:0072163 | BP | mesonephric epithelium development | 0.003725 | 14 |
| GO:0072164 | BP | mesonephric tubule development | 0.003725 | 14 |
| GO:0001656 | BP | metanephros development | 0.029842 | 11 |
| GO:2001014 | BP | regulation of skeletal muscle cell differentiation | 0.000401 | 8 |
| GO:0009208 | BP | pyrimidine ribonucleoside triphosphate metabolic process | 0.002059 | 6 |
| GO:0009147 | BP | pyrimidine nucleoside triphosphate metabolic process | 0.004520 | 6 |
| GO:0009193 | BP | pyrimidine ribonucleoside diphosphate metabolic process | 4.46E-05 | 5 |
| GO:0046048 | BP | UDP metabolic process | 4.46E-05 | 5 |
| GO:0070305 | BP | response to cGMP | 4.46E-05 | 5 |
| GO:0071321 | BP | cellular response to cGMP | 4.46E-05 | 5 |
| GO:0009138 | BP | pyrimidine nucleoside diphosphate metabolic process | 0.000202 | 5 |
| GO:0046036 | BP | CTP metabolic process | 0.000202 | 5 |
| GO:0021512 | BP | spinal cord anterior/posterior patterning | 4.53E-06 | 4 |
| GO:0045967 | BP | negative regulation of growth rate | 4.53E-06 | 4 |
| GO:2000818 | BP | negative regulation of myoblast proliferation | 6.56E-05 | 4 |
| GO:0001978 | BP | regulation of systemic arterial blood pressure by carotid sinus baroreceptor feedback | 0.000559 | 4 |
| GO:0001982 | BP | baroreceptor response to decreased systemic arterial blood pressure | 0.000559 | 4 |
| GO:0003025 | BP | regulation of systemic arterial blood pressure by baroreceptor feedback | 0.000559 | 4 |
| GO:0048632 | BP | negative regulation of skeletal muscle tissue growth | 0.000559 | 4 |
| GO:0014859 | BP | negative regulation of skeletal muscle cell proliferation | 0.003230 | 4 |
| GO:0071444 | BP | cellular response to pheromone | 0.003230 | 4 |
| GO:1902723 | BP | negative regulation of skeletal muscle satellite cell proliferation | 0.003230 | 4 |
| GO:1902725 | BP | negative regulation of satellite cell differentiation | 0.003230 | 4 |
| GO:0019236 | BP | response to pheromone | 0.048324 | 4 |
| GO:2001015 | BP | negative regulation of skeletal muscle cell differentiation | 0.048324 | 4 |
| GO:0004382 | MF | guanosine-diphosphatase activity | 1.03E-06 | 5 |
| GO:0043273 | MF | CTPase activity | 1.03E-06 | 5 |
| GO:0045134 | MF | uridine-diphosphatase activity | 7.82E-06 | 5 |

**Table S9: GO enrichment analysis of Stylommatophora expansion genes.** For each GO subcategory, a 2 × 2 contingency table was constructed by recording the numbers of genes included or not included in a category of ‘genome background’ genes and Stylommatophora expansion genes. Two-tailed Fisher’s exact test was used to calculate statistical significance. BP: biology process; CC: cellular component.

| GO | Type | Function | P value | Gene number |
| --- | --- | --- | --- | --- |
| GO:0032501 | BP | multicellular organismal process | 0.007600 | 832 |
| GO:0050896 | BP | response to stimulus | 3.06E-05 | 807 |
| GO:0007154 | BP | cell communication | 1.91E-08 | 534 |
| GO:0023052 | BP | signaling | 1.88E-10 | 525 |
| GO:0051239 | BP | regulation of multicellular organismal process | 0.010047 | 413 |
| GO:0050793 | BP | regulation of developmental process | 0.004431 | 386 |
| GO:0032879 | BP | regulation of localization | 1.15E-06 | 378 |
| GO:0060429 | BP | epithelium development | 0.011049 | 282 |
| GO:0045595 | BP | regulation of cell differentiation | 0.006087 | 277 |
| GO:0035295 | BP | tube development | 0.005169 | 235 |
| GO:0009791 | BP | post-embryonic development | 2.19E-09 | 229 |
| GO:0040012 | BP | regulation of locomotion | 1.87E-11 | 219 |
| GO:0042325 | BP | regulation of phosphorylation | 0.001597 | 206 |
| GO:0048729 | BP | tissue morphogenesis | 0.012526 | 197 |
| GO:0001932 | BP | regulation of protein phosphorylation | 0.000909 | 194 |
| GO:0051241 | BP | negative regulation of multicellular organismal process | 0.021533 | 190 |
| GO:0007267 | BP | cell-cell signaling | 6.96E-10 | 189 |
| GO:0051270 | BP | regulation of cellular component movement | 5.06E-10 | 187 |
| GO:0002009 | BP | morphogenesis of an epithelium | 0.017699 | 186 |
| GO:0051093 | BP | negative regulation of developmental process | 0.008294 | 173 |
| GO:2000145 | BP | regulation of cell motility | 8.67E-11 | 172 |
| GO:0030334 | BP | regulation of cell migration | 5.97E-11 | 165 |
| GO:0010562 | BP | positive regulation of phosphorus metabolic process | 0.021489 | 150 |
| GO:0045937 | BP | positive regulation of phosphate metabolic process | 0.021489 | 150 |
| GO:0042327 | BP | positive regulation of phosphorylation | 0.004501 | 144 |
| GO:0001934 | BP | positive regulation of protein phosphorylation | 0.001870 | 140 |
| GO:0007444 | BP | imaginal disc development | 0.007684 | 129 |
| GO:0009314 | BP | response to radiation | 0.013070 | 126 |
| GO:0002164 | BP | larval development | 5.71E-07 | 125 |
| GO:0002119 | BP | nematode larval development | 5.18E-10 | 105 |
| GO:0010212 | BP | response to ionizing radiation | 1.15E-11 | 80 |
| GO:0040024 | BP | dauer larval development | 2.92E-23 | 75 |
| GO:0010332 | BP | response to gamma radiation | 2.63E-17 | 61 |
| GO:0034284 | BP | response to monosaccharide | 0.023006 | 46 |
| GO:0009746 | BP | response to hexose | 0.008245 | 45 |
| GO:0060259 | BP | regulation of feeding behavior | 0.004137 | 39 |
| GO:1903998 | BP | regulation of eating behavior | 1.29E-05 | 36 |
| GO:0043051 | BP | regulation of pharyngeal pumping | 2.01E-07 | 35 |
| GO:0030952 | BP | establishment or maintenance of cytoskeleton polarity | 0.000244 | 30 |
| GO:0007229 | BP | integrin-mediated signaling pathway | 0.039414 | 18 |
| GO:0097201 | BP | negative regulation of transcription from RNA polymerase II promoter in response to stress | 0.037923 | 10 |
| GO:0097458 | CC | obsolete neuron part | 1.29E-05 | 278 |
| GO:0036477 | CC | somatodendritic compartment | 3.64E-05 | 186 |
| GO:0044297 | CC | cell body | 5.61E-05 | 162 |
| GO:0048471 | CC | perinuclear region of cytoplasm | 2.10E-07 | 156 |
| GO:0043025 | CC | neuronal cell body | 0.000129 | 150 |

**Table S10: GO enrichment analysis of Stylommatophora contraction genes.** For each GO subcategory, a 2 × 2 contingency table was constructed by recording the numbers of genes included or not included in a category of ‘genome background’ genes and Stylommatophora expansion genes. Two-tailed Fisher’s exact test was used to calculate statistical significance. BP: biology process; CC: cellular component.

| GO | Type | Function | P value | Gene number |
| --- | --- | --- | --- | --- |
| GO:1903961 | BP | positive regulation of anion transmembrane transport | 0.0001013 | 5 |
| GO:1903959 | BP | regulation of anion transmembrane transport | 0.0406884 | 5 |
| GO:2000118 | BP | regulation of sodium-dependent phosphate transport | 0.0019846 | 4 |
| GO:0010966 | BP | regulation of phosphate transport | 0.0110806 | 4 |
| GO:0030647 | BP | aminoglycoside antibiotic metabolic process | 0.0124327 | 3 |
| GO:0042431 | BP | indole metabolic process | 0.0124327 | 3 |
| GO:1901128 | BP | gentamycin metabolic process | 0.0124327 | 3 |
| GO:1901684 | BP | arsenate ion transmembrane transport | 0.0124327 | 3 |
| GO:1903797 | BP | positive regulation of inorganic anion transmembrane transport | 0.0124327 | 3 |
| GO:2000187 | BP | positive regulation of phosphate transmembrane transport | 0.0124327 | 3 |
| GO:0072733 | BP | response to staurosporine | 0.0309616 | 3 |
| GO:0072734 | BP | cellular response to staurosporine | 0.0309616 | 3 |
| GO:2000185 | BP | regulation of phosphate transmembrane transport | 0.0309616 | 3 |
| GO:0072686 | CC | mitotic spindle | 0.0025335 | 8 |
| GO:0031526 | CC | brush border membrane | 0.0216566 | 6 |
| GO:0015370 | MF | solute:sodium symporter activity | 0.0405823 | 6 |
| GO:0005436 | MF | sodium:phosphate symporter activity | 0.0011070 | 4 |

**Table S11: GO enrichment analysis of Stylommatophora positively selected genes.** For each GO subcategory, a 2 × 2 contingency table was constructed by recording the numbers of genes included or not included in a category of ‘genome background’ genes and Stylommatophora positive selected genes. Two-tailed Fisher’s exact test was used to calculate statistical significance. BP: biology process; MF: molecular function.

| GO | Type | Function | P value | Gene number |
| --- | --- | --- | --- | --- |
| GO:0031641 | BP | regulation of myelination | 0.014147 | 4 |
| GO:0007130 | BP | synaptonemal complex assembly | 0.006699 | 3 |
| GO:0004525 | MF | ribonuclease III activity | 0.000227 | 2 |
| GO:0032296 | MF | double-stranded RNA-specific ribonuclease activity | 0.000227 | 2 |
| GO:0031643 | BP | positive regulation of myelination | 3.40E-05 | 3 |

**Table S12: GO enrichment analysis of *Arion vulgaris* species-specific and unassigned genes**. For each GO subcategory, a 2 × 2 contingency table was constructed by recording the numbers of genes included or not included in a category of ‘genome background’ genes and *A. vulgaris* species-specific genes. Two-tailed Fisher’s exact test was used to calculate statistical significance. BP: biology process; CC: cellular component; MF: molecular function.

| GO | Type | Function | P value | Gene number |
| --- | --- | --- | --- | --- |
| GO:0019747 | BP | regulation of isoprenoid metabolic process | 1.46E-05 | 5 |
| GO:0043226 | CC | Organelle | 0.016755 | 259 |
| GO:0043229 | CC | intracellular organelle | 0.004138 | 250 |
| GO:0043227 | CC | membrane-bounded organelle | 0.000500 | 221 |
| GO:0043231 | CC | intracellular membrane-bounded organelle | 0.001050 | 205 |
| GO:0030312 | CC | external encapsulating structure | 1.81E-05 | 10 |
| GO:0015016 | MF | [heparan sulfate]-glucosamine N-sulfotransferase activity | 0.005871 | 3 |

**Fig S13: GO enrichment analysis of *Arion vulgaris* expansion genes**. For each GO subcategory, a 2 × 2 contingency table was constructed by recording the numbers of genes included or not included in a category of ‘genome background’ genes and *A. vulgaris* species-specific genes. Two-tailed Fisher’s exact test was used to calculate statistical significance. BP: biology process; CC: cellular component; MF: molecular function.

| GO | Type | Function | P value | Gene number |
| --- | --- | --- | --- | --- |
| GO:0009607 | BP | response to biotic stimulus | 0.035756 | 309 |
| GO:0043207 | BP | response to external biotic stimulus | 0.00511 | 308 |
| GO:0051707 | BP | response to other organism | 0.008506 | 306 |
| GO:0030029 | BP | actin filament-based process | 0.034447 | 251 |
| GO:0002164 | BP | larval development | 0.000921 | 243 |
| GO:0030036 | BP | actin cytoskeleton organization | 0.045313 | 224 |
| GO:0009617 | BP | response to bacterium | 0.008316 | 222 |
| GO:0071396 | BP | cellular response to lipid | 0.003975 | 212 |
| GO:0048545 | BP | response to steroid hormone | 0.009836 | 175 |
| GO:0009410 | BP | response to xenobiotic stimulus | 0.030106 | 162 |
| GO:0002237 | BP | response to molecule of bacterial origin | 0.010789 | 133 |
| GO:0031099 | BP | Regeneration | 8.95E-05 | 131 |
| GO:0016042 | BP | lipid catabolic process | 8.40E-05 | 129 |
| GO:0071383 | BP | cellular response to steroid hormone stimulus | 5.19E-06 | 127 |
| GO:0032496 | BP | response to lipopolysaccharide | 0.012052 | 126 |
| GO:0061008 | BP | hepaticobiliary system development | 0.001825 | 126 |
| GO:0001889 | BP | liver development | 0.003526 | 123 |
| GO:0031960 | BP | response to corticosteroid | 0.000336 | 123 |
| GO:0010212 | BP | response to ionizing radiation | 0.011636 | 120 |
| GO:0051384 | BP | response to glucocorticoid | 0.000126 | 118 |
| GO:0042440 | BP | pigment metabolic process | 2.00E-05 | 116 |
| GO:0008202 | BP | steroid metabolic process | 0.035907 | 111 |
| GO:0071466 | BP | cellular response to xenobiotic stimulus | 0.009945 | 101 |
| GO:0097306 | BP | cellular response to alcohol | 0.031057 | 99 |
| GO:0010675 | BP | regulation of cellular carbohydrate metabolic process | 0.002573 | 91 |
| GO:0040024 | BP | dauer larval development | 4.26E-09 | 89 |
| GO:0016999 | BP | antibiotic metabolic process | 2.91E-05 | 89 |
| GO:0031100 | BP | animal organ regeneration | 9.21E-07 | 85 |
| GO:0033013 | BP | tetrapyrrole metabolic process | 9.16E-12 | 84 |
| GO:0006805 | BP | xenobiotic metabolic process | 3.87E-05 | 82 |
| GO:0071385 | BP | cellular response to glucocorticoid stimulus | 5.91E-09 | 81 |
| GO:0062014 | BP | negative regulation of small molecule metabolic process | 1.33E-07 | 81 |
| GO:0071384 | BP | cellular response to corticosteroid stimulus | 1.33E-07 | 81 |
| GO:0045833 | BP | negative regulation of lipid metabolic process | 6.69E-06 | 80 |
| GO:0006720 | BP | isoprenoid metabolic process | 4.34E-06 | 79 |
| GO:0007588 | BP | excretion | 4.56E-08 | 78 |
| GO:0010332 | BP | response to gamma radiation | 2.48E-08 | 77 |
| GO:0034754 | BP | cellular hormone metabolic process | 0.001421 | 77 |
| GO:0006778 | BP | porphyrin-containing compound metabolic process | 0 | 76 |
| GO:0019217 | BP | regulation of fatty acid metabolic process | 0.000113 | 76 |
| GO:0006721 | BP | terpenoid metabolic process | 2.62E-08 | 75 |
| GO:0042168 | BP | heme metabolic process | 0 | 74 |
| GO:0001523 | BP | retinoid metabolic process | 6.92E-10 | 73 |
| GO:0016101 | BP | diterpenoid metabolic process | 3.18E-09 | 73 |
| GO:0002526 | BP | acute inflammatory response | 1.83E-11 | 70 |
| GO:0010677 | BP | negative regulation of cellular carbohydrate metabolic process | 4.12E-11 | 69 |
| GO:0045912 | BP | negative regulation of carbohydrate metabolic process | 1.29E-09 | 69 |
| GO:0009225 | BP | nucleotide-sugar metabolic process | 1.16E-07 | 68 |
| GO:0042447 | BP | hormone catabolic process | 0 | 66 |
| GO:0051187 | BP | obsolete cofactor catabolic process | 5.02E-09 | 64 |
| GO:0006953 | BP | acute-phase response | 0 | 63 |
| GO:0071361 | BP | cellular response to ethanol | 2.40E-07 | 63 |
| GO:0071392 | BP | cellular response to estradiol stimulus | 2.33E-08 | 62 |
| GO:0006706 | BP | steroid catabolic process | 3.21E-11 | 61 |
| GO:0045939 | BP | negative regulation of steroid metabolic process | 3.95E-09 | 61 |
| GO:0006011 | BP | UDP-glucose metabolic process | 0 | 60 |
| GO:0045922 | BP | negative regulation of fatty acid metabolic process | 0 | 60 |
| GO:0008210 | BP | estrogen metabolic process | 0 | 60 |
| GO:0042537 | BP | benzene-containing compound metabolic process | 9.16E-12 | 60 |
| GO:0017001 | BP | antibiotic catabolic process | 7.33E-11 | 60 |
| GO:0018879 | BP | biphenyl metabolic process | 0 | 59 |
| GO:0009812 | BP | flavonoid metabolic process | 0 | 59 |
| GO:0042178 | BP | xenobiotic catabolic process | 3.18E-09 | 59 |
| GO:0033015 | BP | tetrapyrrole catabolic process | 0 | 58 |
| GO:0006711 | BP | estrogen catabolic process | 0 | 57 |
| GO:0006787 | BP | porphyrin-containing compound catabolic process | 0 | 57 |
| GO:0006789 | BP | bilirubin conjugation | 0 | 57 |
| GO:0009698 | BP | phenylpropanoid metabolic process | 0 | 57 |
| GO:0009804 | BP | coumarin metabolic process | 0 | 57 |
| GO:0042167 | BP | heme catabolic process | 0 | 57 |
| GO:0046149 | BP | pigment catabolic process | 0 | 57 |
| GO:0046226 | BP | coumarin catabolic process | 0 | 57 |
| GO:0046271 | BP | phenylpropanoid catabolic process | 0 | 57 |
| GO:0051552 | BP | flavone metabolic process | 0 | 57 |
| GO:0052695 | BP | cellular glucuronidation | 0 | 57 |
| GO:0052696 | BP | flavonoid glucuronidation | 0 | 57 |
| GO:0052697 | BP | xenobiotic glucuronidation | 0 | 57 |
| GO:0070980 | BP | biphenyl catabolic process | 0 | 57 |
| GO:1904223 | BP | regulation of glucuronosyltransferase activity | 0 | 57 |
| GO:1904224 | BP | negative regulation of glucuronosyltransferase activity | 0 | 57 |
| GO:2001030 | BP | negative regulation of cellular glucuronidation | 0 | 57 |
| GO:0006063 | BP | uronic acid metabolic process | 0 | 57 |
| GO:0019585 | BP | glucuronate metabolic process | 0 | 57 |
| GO:2001029 | BP | regulation of cellular glucuronidation | 0 | 57 |
| GO:0042573 | BP | retinoic acid metabolic process | 3.67E-11 | 57 |
| GO:0034663 | CC | endoplasmic reticulum chaperone complex | 0 | 61 |
| GO:0070069 | CC | cytochrome complex | 2.02E-10 | 60 |
| GO:0046982 | MF | protein heterodimerization activity | 0.042895 | 212 |
| GO:0031406 | MF | carboxylic acid binding | 0.039347 | 106 |
| GO:0008194 | MF | UDP-glycosyltransferase activity | 0.001112 | 89 |
| GO:0005080 | MF | protein kinase C binding | 0 | 85 |
| GO:0005496 | MF | steroid binding | 7.70E-10 | 79 |
| GO:0033293 | MF | monocarboxylic acid binding | 7.33E-10 | 75 |
| GO:0005504 | MF | fatty acid binding | 0 | 66 |
| GO:0001972 | MF | retinoic acid binding | 0 | 60 |
| GO:0005501 | MF | retinoid binding | 9.16E-12 | 60 |
| GO:0019840 | MF | isoprenoid binding | 2.29E-11 | 60 |
| GO:0015020 | MF | glucuronosyltransferase activity | 5.96E-11 | 59 |

**Table S14: GO enrichment analysis of *Arion vulgaris* contraction genes.** For each GO subcategory, a 2 × 2 contingency table was constructed by recording the numbers of genes included or not included in a category of ‘genome background’ genes and Stylommatophora expansion genes. Two-tailed Fisher’s exact test was used to calculate statistical significance. BP: biology process; CC: cellular component.

| GO | Type | Function | P value | Gene number |
| --- | --- | --- | --- | --- |
| GO:0006811 | BP | ion transport | 0.002125 | 105 |
| GO:0007186 | BP | G protein-coupled receptor signaling pathway | 3.21E-11 | 75 |
| GO:0006820 | BP | anion transport | 0.007451 | 57 |
| GO:0098656 | BP | anion transmembrane transport | 0.027645 | 41 |
| GO:0007218 | BP | neuropeptide signaling pathway | 6.11E-09 | 27 |
| GO:0042749 | BP | regulation of circadian sleep/wake cycle | 0.024221 | 16 |
| GO:0034308 | BP | primary alcohol metabolic process | 0.001884 | 15 |
| GO:0042572 | BP | retinol metabolic process | 0.001194 | 12 |
| GO:0007567 | BP | parturition | 0.001688 | 11 |
| GO:0097070 | BP | ductus arteriosus closure | 0.003929 | 10 |
| GO:0032757 | BP | positive regulation of interleukin-8 production | 0.032495 | 10 |
| GO:0070493 | BP | thrombin-activated receptor signaling pathway | 0.003427 | 8 |
| GO:0033212 | BP | iron import into cell | 0.000132 | 7 |
| GO:0033215 | BP | reductive iron assimilation | 0.000132 | 7 |
| GO:0042817 | BP | pyridoxal metabolic process | 0.013186 | 7 |
| GO:0006710 | BP | androgen catabolic process | 0.001959 | 6 |
| GO:0015746 | BP | citrate transport | 0.048716 | 6 |
| GO:0071944 | CC | cell periphery | 0.000104 | 276 |
| GO:0044425 | CC | obsolete membrane part | 0.005055 | 272 |
| GO:0005886 | CC | plasma membrane | 6.04E-05 | 266 |
| GO:0031224 | CC | intrinsic component of membrane | 1.37E-11 | 201 |
| GO:0016021 | CC | integral component of membrane | 6.87E-11 | 194 |
| GO:0044459 | CC | obsolete plasma membrane part | 0.000562 | 179 |
| GO:0031226 | CC | intrinsic component of plasma membrane | 4.58E-12 | 140 |
| GO:0005887 | CC | integral component of plasma membrane | 1.37E-11 | 135 |
| GO:0031233 | CC | intrinsic component of external side of plasma membrane | 2.26E-05 | 11 |
| GO:0033573 | CC | high-affinity iron permease complex | 0.000132 | 7 |
| GO:1905862 | CC | ferroxidase complex | 0.000132 | 7 |
| GO:0038023 | MF | signaling receptor activity | 2.29E-12 | 83 |
| GO:0060089 | MF | molecular transducer activity | 6.87E-12 | 83 |
| GO:0004888 | MF | transmembrane signaling receptor activity | 2.29E-09 | 64 |
| GO:0004930 | MF | G protein-coupled receptor activity | 1.31E-09 | 44 |
| GO:0008509 | MF | anion transmembrane transporter activity | 0.020526 | 38 |
| GO:0008757 | MF | S-adenosylmethionine-dependent methyltransferase activity | 0.006238 | 29 |
| GO:0008168 | MF | methyltransferase activity | 0.041717 | 29 |
| GO:0001653 | MF | peptide receptor activity | 6.24E-07 | 24 |
| GO:0008528 | MF | G protein-coupled peptide receptor activity | 6.24E-07 | 24 |
| GO:0008188 | MF | neuropeptide receptor activity | 1.55E-06 | 22 |
| GO:0004745 | MF | retinol dehydrogenase activity | 2.19E-08 | 13 |
| GO:0004957 | MF | prostaglandin E receptor activity | 3.10E-05 | 10 |
| GO:0004953 | MF | icosanoid receptor activity | 0.000219 | 10 |
| GO:0004954 | MF | prostanoid receptor activity | 0.000219 | 10 |
| GO:0004955 | MF | prostaglandin receptor activity | 0.000219 | 10 |
| GO:0016725 | MF | oxidoreductase activity, acting on CH or CH2 groups | 0.000219 | 10 |
| GO:0016722 | MF | oxidoreductase activity, oxidizing metal ions | 0.016515 | 9 |
| GO:0042895 | MF | obsolete antibiotic transmembrane transporter activity | 0.016515 | 9 |
| GO:0004322 | MF | ferroxidase activity | 0.004202 | 7 |
| GO:0016724 | MF | oxidoreductase activity, oxidizing metal ions, oxygen as acceptor | 0.004202 | 7 |
| GO:0004031 | MF | aldehyde oxidase activity | 0.013186 | 7 |
| GO:0004732 | MF | pyridoxal oxidase activity | 0.013186 | 7 |
| GO:0016404 | MF | 15-hydroxyprostaglandin dehydrogenase (NAD+) activity | 0.013186 | 7 |
| GO:0016623 | MF | oxidoreductase activity, acting on the aldehyde or oxo group of donors, oxygen as acceptor | 0.013186 | 7 |
| GO:0015137 | MF | citrate transmembrane transporter activity | 0.048716 | 6 |

**Fig S15: GO enrichment analysis of *Arion vulgaris* positive selected genes**. For each GO subcategory, a 2 × 2 contingency table was constructed by recording the numbers of genes included or not included in a category of ‘genome background’ genes and *A. vulgaris* species-specific genes. Two-tailed Fisher’s exact test was used to calculate statistical significance. BP: biology process; CC: cellular component; MF: molecular function.

| GO | Type | Function | P value | Gene number |
| --- | --- | --- | --- | --- |
| GO:0051099 | BP | positive regulation of binding | 0.001346 | 4 |
| GO:0009794 | BP | regulation of mitotic cell cycle, embryonic | 0.001047 | 2 |
| GO:0042509 | BP | regulation of tyrosine phosphorylation of STAT protein | 0.009004 | 2 |
| GO:0042531 | BP | positive regulation of tyrosine phosphorylation of STAT protein | 2.92E-05 | 2 |
| GO:0051769 | BP | regulation of nitric-oxide synthase biosynthetic process | 1.01E-06 | 2 |
| GO:0051770 | BP | positive regulation of nitric-oxide synthase biosynthetic process | 8.16E-12 | 2 |
| GO:1900744 | BP | regulation of p38MAPK cascade | 0.002657 | 2 |
| GO:0002731 | BP | negative regulation of dendritic cell cytokine production | 1.54E-10 | 1 |
| GO:0002825 | BP | regulation of T-helper 1 type immune response | 0.001017 | 1 |
| GO:0002826 | BP | negative regulation of T-helper 1 type immune response | 1.54E-10 | 1 |
| GO:0006535 | BP | cysteine biosynthetic process from serine | 1.54E-10 | 1 |
| GO:0006565 | BP | L-serine catabolic process | 0.001017 | 1 |
| GO:0007260 | BP | tyrosine phosphorylation of STAT protein | 1.54E-10 | 1 |
| GO:0009092 | BP | homoserine metabolic process | 0.001017 | 1 |
| GO:0009093 | BP | cysteine catabolic process | 0.024452 | 1 |
| GO:0009403 | BP | toxin biosynthetic process | 5.28E-06 | 1 |
| GO:0018343 | BP | protein farnesylation | 0.024452 | 1 |
| GO:0019346 | BP | transsulfuration | 0.001017 | 1 |
| GO:0019448 | BP | L-cysteine catabolic process | 0.024452 | 1 |
| GO:0030223 | BP | neutrophil differentiation | 0.001017 | 1 |
| GO:0031959 | BP | mineralocorticoid receptor signaling pathway | 1.54E-10 | 1 |
| GO:0032693 | BP | negative regulation of interleukin-10 production | 0.024452 | 1 |
| GO:0034050 | BP | host programmed cell death induced by symbiont | 1.54E-10 | 1 |
| GO:0034477 | BP | U6 snRNA 3'-end processing | 1.54E-10 | 1 |
| GO:0035406 | BP | histone-tyrosine phosphorylation | 1.54E-10 | 1 |
| GO:0035409 | BP | histone H3-Y41 phosphorylation | 1.54E-10 | 1 |
| GO:0035771 | BP | interleukin-4-mediated signaling pathway | 1.54E-10 | 1 |
| GO:0035905 | BP | ascending aorta development | 0.001017 | 1 |
| GO:0035910 | BP | ascending aorta morphogenesis | 0.001017 | 1 |
| GO:0036015 | BP | response to interleukin-3 | 0.001017 | 1 |
| GO:0036016 | BP | cellular response to interleukin-3 | 0.001017 | 1 |
| GO:0038113 | BP | interleukin-9-mediated signaling pathway | 1.54E-10 | 1 |
| GO:0038114 | BP | interleukin-21-mediated signaling pathway | 1.54E-10 | 1 |
| GO:0038155 | BP | interleukin-23-mediated signaling pathway | 0.001017 | 1 |
| GO:0042262 | BP | DNA protection | 0.024452 | 1 |
| GO:0042976 | BP | activation of Janus kinase activity | 5.28E-06 | 1 |
| GO:0043418 | BP | homocysteine catabolic process | 5.28E-06 | 1 |
| GO:0044375 | BP | regulation of peroxisome size | 5.28E-06 | 1 |
| GO:0044854 | BP | plasma membrane raft assembly | 0.024452 | 1 |
| GO:0044857 | BP | plasma membrane raft organization | 0.024452 | 1 |
| GO:0045219 | BP | regulation of FasL production | 1.54E-10 | 1 |
| GO:0045221 | BP | negative regulation of FasL production | 1.54E-10 | 1 |
| GO:0045625 | BP | regulation of T-helper 1 cell differentiation | 5.28E-06 | 1 |
| GO:0045626 | BP | negative regulation of T-helper 1 cell differentiation | 1.54E-10 | 1 |
| GO:0051316 | BP | attachment of spindle microtubules to kinetochore involved in meiotic chromosome segregation | 0.024452 | 1 |
| GO:0060031 | BP | mediolateral intercalation | 5.28E-06 | 1 |
| GO:0060355 | BP | positive regulation of cell adhesion molecule production | 0.001017 | 1 |
| GO:0060399 | BP | positive regulation of growth hormone receptor signaling pathway | 0.001017 | 1 |
| GO:0070106 | BP | interleukin-27-mediated signaling pathway | 1.54E-10 | 1 |
| GO:0070757 | BP | interleukin-35-mediated signaling pathway | 1.54E-10 | 1 |
| GO:0070813 | BP | hydrogen sulfide metabolic process | 0.001017 | 1 |
| GO:0070814 | BP | hydrogen sulfide biosynthetic process | 5.28E-06 | 1 |
| GO:0071104 | BP | response to interleukin-9 | 1.54E-10 | 1 |
| GO:0071355 | BP | cellular response to interleukin-9 | 1.54E-10 | 1 |
| GO:0098756 | BP | response to interleukin-21 | 1.54E-10 | 1 |
| GO:0098757 | BP | cellular response to interleukin-21 | 1.54E-10 | 1 |
| GO:0106005 | BP | RNA 5'-cap (guanine-N7)-methylation | 1.54E-10 | 1 |
| GO:1902724 | BP | positive regulation of skeletal muscle satellite cell proliferation | 1.54E-10 | 1 |
| GO:1902728 | BP | positive regulation of growth factor dependent skeletal muscle satellite cell proliferation | 1.54E-10 | 1 |
| GO:1905457 | BP | negative regulation of lymphoid progenitor cell differentiation | 0.001017 | 1 |
| GO:2000049 | BP | positive regulation of cell-cell adhesion mediated by cadherin | 0.024452 | 1 |
| GO:2000668 | BP | regulation of dendritic cell apoptotic process | 1.54E-10 | 1 |
| GO:2000670 | BP | positive regulation of dendritic cell apoptotic process | 1.54E-10 | 1 |
| GO:2000974 | BP | negative regulation of pro-B cell differentiation | 0.001017 | 1 |
| GO:2000978 | BP | negative regulation of forebrain neuron differentiation | 0.001017 | 1 |
| GO:0044450 | CC | obsolete microtubule organizing center part | 0.000180 | 4 |
| GO:0034451 | CC | centriolar satellite | 0.000630 | 2 |
| GO:0005827 | CC | polar microtubule | 0.024452 | 1 |
| GO:0005965 | CC | protein farnesyltransferase complex | 5.28E-06 | 1 |
| GO:0008275 | CC | gamma-tubulin small complex | 0.001017 | 1 |
| GO:0031533 | CC | mRNA cap methyltransferase complex | 1.54E-10 | 1 |
| GO:0004527 | MF | exonuclease activity | 0.000160 | 3 |
| GO:0004028 | MF | 3-chloroallyl aldehyde dehydrogenase activity | 1.54E-10 | 1 |
| GO:0004030 | MF | aldehyde dehydrogenase [NAD(P)+] activity | 0.024452 | 1 |
| GO:0004122 | MF | cystathionine beta-synthase activity | 1.54E-10 | 1 |
| GO:0004124 | MF | cysteine synthase activity | 1.54E-10 | 1 |
| GO:0004311 | MF | farnesyltranstransferase activity | 0.001017 | 1 |
| GO:0004362 | MF | glutathione-disulfide reductase activity | 1.54E-10 | 1 |
| GO:0004482 | MF | mRNA (guanine-N7-)-methyltransferase activity | 1.54E-10 | 1 |
| GO:0004660 | MF | protein farnesyltransferase activity | 5.28E-06 | 1 |
| GO:0005119 | MF | smoothened binding | 5.28E-06 | 1 |
| GO:0005131 | MF | growth hormone receptor binding | 0.001017 | 1 |
| GO:0005143 | MF | interleukin-12 receptor binding | 1.54E-10 | 1 |
| GO:0015038 | MF | glutathione disulfide oxidoreductase activity | 1.54E-10 | 1 |
| GO:0016662 | MF | oxidoreductase activity, acting on other nitrogenous compounds as donors, cytochrome as acceptor | 5.28E-06 | 1 |
| GO:0017005 | MF | 3'-tyrosyl-DNA phosphodiesterase activity | 0.001017 | 1 |
| GO:0031730 | MF | CCR5 chemokine receptor binding | 5.28E-06 | 1 |
| GO:0035400 | MF | histone tyrosine kinase activity | 1.54E-10 | 1 |
| GO:0035401 | MF | histone kinase activity (H3-Y41 specific) | 1.54E-10 | 1 |
| GO:0048020 | MF | CCR chemokine receptor binding | 0.001017 | 1 |
| GO:0050421 | MF | nitrite reductase (NO-forming) activity | 5.28E-06 | 1 |
| GO:0051428 | MF | peptide hormone receptor binding | 1.54E-10 | 1 |
| GO:0070259 | MF | tyrosyl-DNA phosphodiesterase activity | 0.024452 | 1 |
| GO:0071568 | MF | UFM1 transferase activity | 5.28E-06 | 1 |
| GO:0071820 | MF | N-box binding | 0.001017 | 1 |
| GO:0098605 | MF | selenocystathionine beta-synthase activity | 1.54E-10 | 1 |
| GO:0098622 | MF | selenodiglutathione-disulfide reductase activity | 1.54E-10 | 1 |
| GO:0098809 | MF | nitrite reductase activity | 5.28E-06 | 1 |

**Table S16. Number of gene pairs derived from different modes of duplication in seven Heterobranchia species.** WGD: whole-genome duplication, DSD: dispersed duplication, TRD: transposed duplication, PD: proximal duplication, TD: tandem duplication.

| Species | DSD | PD | TD | TRD | WGD |
| --- | --- | --- | --- | --- | --- |
| *Lissachatina* (*Achatina*) *fulica* | 4,685 | 1,246 | 2,660 | 3,492 | 5,251 |
| *Li. immaculata* | 10,905 | 1,056 | 2,653 | 3,585 | 4,021 |
| ***Arion vulgaris*** | 10,035 | 1,030 | 4,432 | 3,268 | 4,999 |
| *Radix auricularia* | 6,940 | 601 | 1,776 | 56 | 0 |
| *Biomphalaria glabrata* | 13,954 | 663 | 2,281 | 128 | 0 |
| *Elysia chlorotica* | 11,655 | 573 | 1,410 | 78 | 0 |
| *Aplysia californica* | 6,111 | 270 | 7,488 | 24 | 0 |

**Table S17. Number of positive selected gene pairs (*Ka*/*Ks*>1) derived from different modes in seven Heterobranchia species.** The brackets show the percentages of gene pairs with Ka/Ks >1 in the corresponding duplicated gene pairs. WGD: whole-genome duplication, DSD: dispersed duplication, TRD: transposed duplication, PD: proximal duplication, TD: tandem duplication.

| Species | DSD | PD | TD | TRD | WGD |
| --- | --- | --- | --- | --- | --- |
| *Lissachatina* (*Achatina*) *fulica* | 369  (7.8%) | 47  (3.77%) | 119  (4.47%) | 51  (1.46%) | 2  (0.04%) |
| *Li. immaculata* | 1,777  (16.3%) | 39  (3.69%) | 168  (6.33%) | 88  (2.45%) | 12  (0.30%) |
| ***Arion vulgaris*** | 2,041  (20.3%) | 47  (4.56%) | 648  (14.62%) | 46  (1.41%) | 4  (0.08%) |
| *Radix auricularia* | 38  (0.55%) | 3  (0.50%) | 9  (0.51%) | 0 | 0 |
| *Biomphalaria glabrata* | 391  (2.80%) | 2  (0.30%) | 19  (0.83%) | 0 | 0 |
| *Elysia chlorotica* | 400  (3.43%) | 11  (1.92%) | 20  (1.42%) | 0 | 0 |
| *Aplysia californica* | 61  (1.00%) | 8  (2.96%) | 289  (3.86%) | 0 | 0 |

**Table S18. Identified repeat classes in the *Arion vulgaris* genome.** TEs: transposable elements; DNAs: DNA transposons; LTR: Long Terminal Repeat; LINEs: Long Interspersed Nuclear Elements; RC: Rolling Circle; SINEs: Short Interspersed Nuclear Elements;

| Repeat type | | Repeat size (bp) | % Of genome |
| --- | --- | --- | --- |
| **TEs** | | 941,228,221 | 61.08 |
|  | LINE | 561,000,000 | 36.39 |
|  | DNA | 83,828,750 | 5.44 |
|  | SINE | 27,404,787 | 1.78 |
|  | LTR | 17,654,456 | 1.15 |
|  | RC | 10,002,034 | 0.65 |
|  | Satellite | 8,990,082 | 0.58 |
|  | low | 864,980 | 0.06 |
| Unknown | | 274,000,000 | 17.76 |
| **Tandem Repeats** | | 326,134,855 | 21.14 |
| **Total** | | 1,158,337,369 | 75.09 |

**Table S19. Number of different TEs types in different genic regions in six** **Heterobranchia species.** Down: 1kb down-3’UTR, up: 2kb up-5’UTR. %Total shows the percentage of different type of TEs in all types of TEs in correspond genic region. DNAs: DNA transposons; LTR: Long Terminal Repeat; LINEs: Long Interspersed Nuclear Elements; SINEs: Short Interspersed Nuclear Elements;

|  | Region | DNA | % Total | LINE | % Total | LTR | % Total | SINE | % Total | Unknown | % Total | Total |
| --- | --- | --- | --- | --- | --- | --- | --- | --- | --- | --- | --- | --- |
| *Lissachatina* (*Achatina*) f*ulica* | down | 4,504 | 0.20 | 12,863 | 0.57 | 4,231 | 0.19 | 9 | 0.0004 | 43 | 0.002 | 22,615 |
|  | exon | 1,449 | 0.19 | 4,270 | 0.56 | 1,572 | 0.20 | 3 | 0.0004 | 43 | 0.006 | 7,686 |
|  | intron | 119,603 | 0.19 | 363,202 | 0.59 | 109,786 | 0.18 | 236 | 0.0004 | 1,442 | 0.002 | 618,462 |
|  | up | 11,519 | 0.21 | 30,769 | 0.56 | 9,749 | 0.18 | 18 | 0.0003 | 157 | 0.003 | 54,531 |
| *Li. immaculata* | down | 5,076 | 0.44 | 3,734 | 0.32 | 1,307 | 0.11 | 76 | 0.0066 | 75 | 0.006 | 11,539 |
|  | exon | 902 | 0.33 | 533 | 0.20 | 942 | 0.35 | 33 | 0.0121 | 42 | 0.015 | 2,728 |
|  | intron | 76,437 | 0.39 | 80,549 | 0.41 | 18,104 | 0.09 | 1,151 | 0.0059 | 1,210 | 0.006 | 194,095 |
|  | up | 12,532 | 0.43 | 9,851 | 0.34 | 3,131 | 0.11 | 221 | 0.0076 | 207 | 0.007 | 28,914 |
| ***Arion vulgaris*** | down | 4,004 | 0.09 | 23,634 | 0.51 | 1,156 | 0.02 | 1,501 | 0.0322 | 15,716 | 0.337 | 46,632 |
|  | exon | 1,046 | 0.27 | 1,411 | 0.36 | 774 | 0.20 | 70 | 0.0180 | 468 | 0.120 | 3,888 |
|  | intron | 55,905 | 0.07 | 421,028 | 0.53 | 14,180 | 0.02 | 25,272 | 0.0317 | 272,362 | 0.341 | 797,679 |
|  | up | 8,980 | 0.09 | 51,112 | 0.52 | 2,287 | 0.02 | 2,839 | 0.0288 | 32,124 | 0.325 | 98,719 |
| *Biomphalaria glabrata* | down | 2,823 | 0.13 | 5,571 | 0.26 | 381 | 0.02 | 1,857 | 0.0878 | 4,455 | 0.211 | 21,156 |
|  | exon | 262 | 0.12 | 249 | 0.11 | 168 | 0.08 | 55 | 0.0249 | 120 | 0.054 | 2,212 |
|  | intron | 29,924 | 0.13 | 58,573 | 0.26 | 3,700 | 0.02 | 20,958 | 0.0920 | 48,195 | 0.212 | 227,804 |
|  | up | 6,936 | 0.15 | 13,318 | 0.28 | 870 | 0.02 | 3,154 | 0.0671 | 10,181 | 0.217 | 47,012 |
| *Elysia chlorotica* | down | 4,616 | 0.45 | 1,629 | 0.16 | 1,525 | 0.15 | 45 | 0.0043 | 209 | 0.020 | 10,365 |
|  | exon | 1,980 | 0.33 | 1,765 | 0.30 | 1,113 | 0.19 | 5 | 0.0008 | 87 | 0.015 | 5,919 |
|  | intron | 51,275 | 0.43 | 16,579 | 0.14 | 16,836 | 0.14 | 659 | 0.0056 | 2,896 | 0.024 | 118,330 |
|  | up | 12,641 | 0.44 | 4,555 | 0.16 | 4,164 | 0.14 | 119 | 0.0041 | 601 | 0.021 | 28,753 |
| *Radix auricularia* | down | 3,101 | 0.36 | 1,438 | 0.17 | 46 | 0.01 | 92 | 0.0106 | 3,725 | 0.431 | 8,645 |
|  | exon | 4 | 0.22 | 6 | 0.33 | 1 | 0.06 | 1 | 0.0556 | 6 | 0.333 | 18 |
|  | intron | 41,113 | 0.34 | 15,372 | 0.13 | 1,051 | 0.01 | 1,126 | 0.0094 | 58,573 | 0.487 | 120,394 |
|  | up | 9,837 | 0.37 | 3,762 | 0.14 | 171 | 0.01 | 206 | 0.0078 | 11,845 | 0.446 | 26,535 |

**References**

1 Liu, C. *et al.* Giant African snail genomes provide insights into molluscan whole-genome duplication and aquatic-terrestrial transition. *Mol. Ecol. Resour.* **21,** 478-494 (2020).

2 Guo, Y. *et al.* A chromosomal-level genome assembly for the giant African snail Achatina fulica. *Gigascience* **8,** giz124 (2019).

3 Di Palma, F. *et al.* *The Draft Genome of Aplysia californica*, https://www.ncbi.nlm.nih.gov/nuccore/AASC00000000.3 (2014).

4 Adema, C. M. *et al.* Whole genome analysis of a schistosomiasis-transmitting freshwater snail. *Nat. Commun.* **8,** 15451 (2017).

5 Cai, H. *et al.* A draft genome assembly of the solar-powered sea slug Elysia chlorotica. *Sci. Data* **6,** 190022 (2019).

6 Schell, T. *et al.* An annotated draft genome for Radix auricularia (Gastropoda, Mollusca). *Genome Biol. Evol.* **9,** 585–592 (2017).

7 Sun, J. *et al.* Signatures of divergence, invasiveness, and terrestrialization revealed by four apple snail genomes. *Mol. Biol. Evol.* **36,** 1507-1520 (2019).

8 Andreson, R. *et al.* Gene content of the fish-hunting cone snail Conus consors. Preprint at https://doi.org/10.1101/590695 (2019).

9 Masonbrink, R. E. *et al.* An annotated genome for Haliotis rufescens (red abalone) and resequenced green, pink, pinto, black, and white abalone species. *Genome Biol. Evol.* **11,** 431-438 (2019).

10 Sun, J. *et al.* The Scaly-foot snail genome and implications for the origins of biomineralised armour. *Nat. Commun.* **11,** 1657 (2020).

11 Simakov, O. *et al.* Insights into bilaterian evolution from three spiralian genomes. *Nature* **493**, 526-531 (2013).

12 Li, C. *et al.* Draft genome of the Peruvian scallop Argopecten purpuratus. *Gigascience* **7,** giy031 (2018).

13 Powell, D. *et al.* The genome of the oyster Saccostrea offers insight into the environmental resilience of bivalves. *DNA Res.* **25,** 655-665 (2018).
